# Supplementary material for: Genetic Code Expansion for Site-Specific Encoding of a Switchable, Intrinsic Fluorophore-Quencher Pair to Monitor Protein Dynamics
Source: bioRxiv. 2026 Jun 29:2026.06.26.734876. Preprint. [Version 1] doi: 10.64898/2026.06.26.734876 (PMC13345037; doi:10.64898/2026.06.26.734876)
Supplement: Supplement 1 [file media-1.pdf]

Supporting Information

Genetic Code Expansion for Site-Specific Encoding of a Switchable, Intrinsic Fluorophore-Quencher Pair to Monitor Protein Dynamics

Priyanda Giri,[a],# Yarra Venkatesh,[a],# Moriah H. Mathis,[b] Christina M. Hurley,[c] Chloe M. Jones,[c] Olivier Ndogo Eteme,[a],[d] Zachary M. Hostetler,[e] Richard B. Cooley,[b] Rahul M. Kohli,[e],[f] Ryan A. Mehl,[b] E. James Petersson*[a][f]

[a] Department of Chemistry, School of Arts and Sciences
University of Pennsylvania
231 South 34th Street, Philadelphia, PA 19104, USA
E-mail: ejpetersson@sas.upenn.edu

[b] Department of Biochemistry & Biophysics
Oregon State University
Corvallis, OR 97331, USA.

[c] Graduate Group in Biochemistry and Molecular Biophysics, Perelman School of Medicine
University of Pennsylvania
3620 Hamilton Walk, Philadelphia, PA 19104, USA

[d] Department of Organic Chemistry, Faculty of Science
University of Yaoundé I
Yaoundé, Cameroon

[e] Department of Medicine, Perelman School of Medicine
University of Pennsylvania
3400 Civic Center Boulevard, Philadelphia, PA 19104, USA

[f] Department of Biochemistry and Biophysics, Perelman School of Medicine
University of Pennsylvania
421 Curie Boulevard, Philadelphia, PA 19104, USA

#These authors contributed equally to this work.

*E-mail: E.J.P: ejpetersson@sas.upenn.edu

Table of Contents

General Information	S4
Stern-Volmer Analysis of Tet* Quenching Acd	S5
Fluorescence Lifetime Measurement of Acd in the Presence of Tet*	S6
Synthesis, Purification, and Characterization of Proline Ruler with Tet* and Acd	S6
Proline Ruler FRET Measurements	S12
Dequenching of Proline Ruler Peptides	S15
Cloning of Dual Label Plasmids: CaM, LexA, and RecA	S17
Cloning of Synthetase Plasmids, Sequence Maps, and Primers Used	S19
Reagents and Buffers for Bacterial Culture	S23
Overexpression of CaM, RecA and LexA with Tet and Acd	S26
Overexpression of WT-RecA and WT-LexA	S28
Purification and Characterization of CaM$\tau_{93}\delta_{113}$	S29
Purification and Characterization of RecA$\tau_{33}\delta_{102}$	S31
Purification and Characterization of LexA$\tau_{81}\delta_{86}$	S32
Purification and Characterization of LexA$\delta_{81}\tau_{86}$	S34
Purification and Characterization of LexA$\tau_{86}\delta_{175}$ (K156A)	S35
Purification and Characterization of LexA$\tau_{86}\delta_{175}$ (QM-L89P, Q92W, E152A, and K156A)	S36
Purification and Characterization of LexAδ_{175}(K156A) and LexAd$_{175}$(QM)	S37
Oxidation of DihydroTet to Tet by Horseradish Peroxidase (HRP)	S38
Protein Yields and MALDI-MS Analysis of *E. coli* Tet and Acd Expression Systems	S39
Conformational Changes in CaM Upon Ca^{2+} Sensing and Tet Photolysis	S41
LexA Cleavage	S43
RecA Activation Reactions	S45
Fluorescence Anisotropy Measurements	S45
Fluorescence Anisotropy Measurements for LexA$\tau_{86}\delta_{175}$ (QM)	S48
Fluorescence Quenching Measurements (LexA conformational change)	S50
Alternate Models Tested for Conformational Change Step	S53
LexA Cleavage Kinetics Using Fluorescence Intensity Measurement	S58
Control Experiment to Identify the Effect of Surrounding Amino Acids on Acd Fluorescence Quenching	S61

RecA*-Mediated LexA$\delta_{81}\tau_{86}$ FRET Biosensor Assay	S62
IC_{50} curve for Compound #2	S63
Chemical Structure of small molecules screened	S63
NMR Spectra of #1 and #2	S65
Crystal Structure of #1	S68
Crystal Structure of #2	S74
Synthetase Sequences	S80
References	S83

General Information

Materials. Fluorenylmethoxycarbonyl-Pro-OH was purchased from Novabiochem (currently EMD Millipore, MilliporeSigma; Burlington, MA, USA). Benzotriazol-1-yl-oxy-tris(dimethylamino) phosphonium hexafluorophosphate (BOP) was purchased from Chem-Impex (Wood Dale, IL, USA). Piperidine, 4-(1,2,4,5-Tetrazin-3-yl)benzeneacetic acid (Tet*) and *N,N*-diisopropylethylamine (DIPEA) were purchased from Sigma-Aldrich (St. Louis, MO, USA). All other reagents were purchased from Fisher Scientific (Pittsburg, PA, USA) unless specified otherwise. Milli-Q filtered (18 MΩ) water was used for all solutions (EMD Millipore). *E. coli* BL21 DE3 electrocompetent cells were purchased from MilliporeSigma (CMC0016, 20 x 50 μL). Amicon Ultra centrifugal filter units (3 kDa or 10 kDa MWCO) were purchased from EMD Millipore. The noncanonical amino acids (ncAAs) acridon-2-ylalanine (Acd) and 2-amino-3-(3-(6-methyl-1,2,4,5-tetrazin-3-yl)phenyl)propanoic acid hydrochloride (previously referred to as Tet3.0Me, here simply Tet) were synthesized using previously reported methods.[1-2] DNA sequencing was performed at the University of Pennsylvania sequencing facility unless otherwise noted. The DC protein assay kit was purchased from BioRad (Hercules, CA, USA). Primers were purchased from Integrated DNA Technologies (Coralville, IA, USA). dNTPs and Agarose were purchased from Invitrogen (ThermoFisher, Waltham, MA, USA). Q5 High-Fidelity 2X Master Mix, Gel Green DNA gel stain was purchased from Biotium (Freemont, CA, USA). DNA Clean & Concentrator Kit and Plasmid Miniprep Kit were purchased from Zymo Research (Irvine, CA, USA). Pierce™ Horseradish Peroxidase (Catalog number: 31490) was purchased from Fisher Scientific, USA. BL21(DE3) Electrocompetent Cells (Catalog number: CMC0016) were purchased from MilliporeSigma, USA.

Instruments. Proteins were purified with an Agilent 1260 Infinity II Preparative HPLC system and an Agilent 1260 Infinity II Analytical HPLC system (Santa Clara, CA, USA). Protein mass spectrometry data collected Matrix assisted laser desorption ionization (MALDI) mass spectrometry (MS) data were acquired on a Bruker Rapiflex instrument with a standard sinapic acid matrix. Peptide mass spectrometry data were collected with a Bruker MicrofleX (Billerica, MA, USA) MALDI MS using a standard α-cyano-4-hydroxycinnamic acid (CHCA) matrix. Absorbance readings for the DC assay, fluorescence intensity, fluorescence spectral, and fluorescence polarization measurements were made on a Tecan Spark plate reader (Mannedorf, Switzerland). Photolysis of Tet was performed with 7.2 watts USHIO G8T5 Low-Pressure Mercury-Arc lamps that emit radiation peaking at 253.7 nm (UV-C) (Japan). Acd fluorescence was visualized by illuminating the gels using G:Box mini (Syngene), exciting at 365 nm.

Stern-Volmer Analysis of Tet* Quenching Acd

The experimental procedure for the Stern-Volmer experiments was followed as previously described, with minor modifications.[3] Stocks of 4 mM Acd and 1 M Tet* in 100 mM phosphate buffer, pH 7.0, were used to prepare samples that were uniform in Acd concentration of 100 µM and variable in Tet* concentration (0, 0.1, 0.5, 1, 2, 5, 8, 10, 12, 15, 20 mM). For sTCO-OH modification of Tet*, 50 mM sTCO-OH was added to the above Tet* concentrations and incubated for 15 minutes with shaking (500 rpm) at 37 °C. Fluorescence spectra for each sample were collected at 450 nm with an excitation wavelength of 385 nm in triplicate using Tecan Spark plate reader (5 nm bandwidth, 40 µs integration time, 31000 µm Z-position, 4 sec linear shaking) with a nonsterile Greiner black, flat µClear, 384 well low volume microplate. The data fitted to Equation S1 using GraphPad Prism (version 10.0.0, GraphPad Software, Boston, Massachusetts USA)

$$\frac{F_0}{F} - 1 = K_{SV}[Q] \quad \text{(S1)}$$

F_0 is the average fluorescence intensity at 450 nm in the absence of quencher; F is the fluorescence intensity at 450 nm at each Tet* concentration step; K_{SV} is the Stern-Volmer constant in M^{-1}; and [Q] is the concentration of the quencher (Tet*) in M (Figure S1).

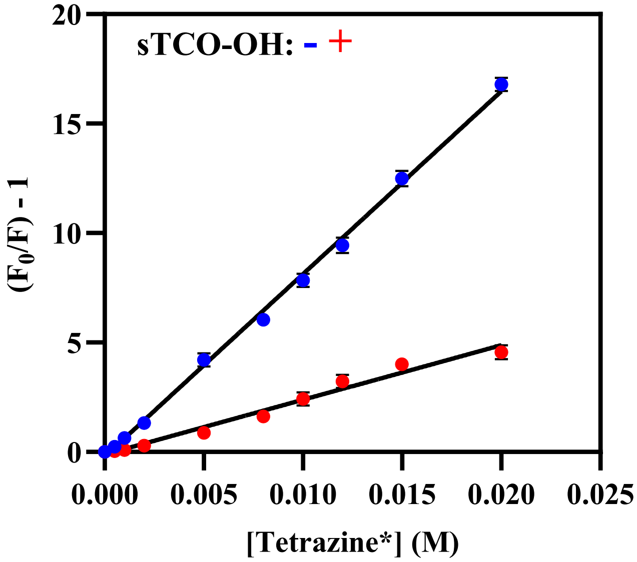

sTCO-OH	K_{SV} (M^{-1})	R^2
–	834.3 ± 8.56	0.9967
+	249.5 ± 7.22	0.9859

Figure S1. Left: Stern-Volmer plots of Tet* quenching Acd. Error bars are calculated from standard error. Right: Stern-Volmer constants and goodness-of-fit values for the fits of Equation S1.

Fluorescence Lifetime Measurement of Acd in the Presence of Tet*

TCSPC measurements of fluorescence lifetime decays for 5 μM of Acd (in water) in the presence of 0 mM, 2.5 mM, and 5 mM Tet (in water) were collected with Horiba Fluoromax spectrometer using a 340 nm excitation and emission was collected at 485 nm with 20 nm slit widths with a 408 nm long pass filter placed between the sample and the emission monochromator. The instrument response function (IRF) was collected under identical conditions. Data analysis was performed with FluoFit software (PicoQuant GmbH; Berlin, Germany) using an exponential decay model.

Table S1. Fluorescence lifetime of Acd at different Tet concentrations.

Conc.of Tet (mM)	Acd Lifetime (ns) in H_2O	χ^2
0	14.56 ± 0.154	1.611
2.5	12.49 ± 0.254	1.151
5	10.08 ±0.185	1.097

Synthesis, Purification, and Characterization of Proline Ruler with Tet* and Acd

Peptide Synthesis and Purification. Tet*-Pro_n-Acd, n= 2-14, and Leu-Pro_4-Acd were synthesized on a 25 μmol scale on 2-chlorotrityl resin. Tet*-Pro_n-Acd, n= 2-8, were synthesized from a common pot of resin (175 μmol); Tet*-Pro_n-Acd, n= 9-14, and Leu-Pro_4-Acd were synthesized from another common pot of resin (175 μmol). After coupling the appropriate number of proline residues, a 25 μmol portion of resin was removed from the reaction vessel (RV) and transferred to a separate RV for Tet* or Leu coupling. For each synthesis, 2-chlorotrityl chloride resin (100-200 mesh; 1.48 mmol/g; 175 mol) was added to a 6 mL polypropylene syringe (RV). The resin was swollen by incubating it for 45 mins in a (9:1 v/v) mixture of dichloromethane (DCM) and dimethylformamide (DMF) and magnetic stirring. After swelling, the DCM/DMF mixture was removed with vacuum suction, and Fmoc-Acd-OH was coupled to the resin. The amino acid in DMF (2 mL) and DIPEA (4 equiv; 121.8 μL) was added to the RV, and the mixture was allowed to react for two successive 30 min incubations with magnetic stirring. The spent solution was removed with vacuum suction, and the resin beads were cleaned thoroughly with successive DMF and DCM washes. The resin beads were then deprotected by treatment with 20% piperidine in DMF (3 mL) twice for 10 mins each with magnetic stirring. The deprotection solution was drained from the RV, and the beads were rinsed extensively with successive DMF and DCM washes. Subsequent amino acid couplings and deprotections proceeded as described above, with the exception that the Fmoc-Pro-OH and Tet* acid were activated with HBTU (4.8 equiv.) and BOP (1.9 equiv.), respectively, before addition to each reaction. The N-terminal Fmoc group in Leu was removed and acetylated with acetic anhydride (500 μL) and *N*-methyl morpholine (300 μL) in DMF (4.2 mL). The mixture was allowed to react twice for 10 mins each. After the beads were washed extensively with DMF and dried with DCM, peptides were cleaved by 60 mins incubation on a rotisserie with 2 mL of a fresh cleavage cocktail of trifluoracetic acid (TFA) and water (1:1 v/v). The resultant solution was reduced to less than 1 mL by blowing off TFA with argon gas. This solution was then treated with 20 mL of cold ethyl ether to precipitate the peptide. This precipitate

was flash frozen with liquid nitrogen and evaporated using a lyophilizer (Labconco; Kansas City, MO, USA).

The crude peptide was diluted in CH_3CN/H_2O (10:90 v/v) and then purified on a Luna®Omega 5 µm PS C18 100 Å, LC semi-preparative column (Phenomenex; Torrance, CA, USA) by HPLC using Gradient 1 for Tet*-Pro_n-Acd, n= 2-5 and Leu-Pro_4-Acd; Gradient 2 for Tet*-Pro_n-Acd, n= 6-8; and Gradient 3 for Tet*-Pro_n-Acd, n= 9-14 (Table S2 and S3). MALDI MS and LC-MS were used to confirm peptide identity (Table S4 and Figure S3). Purified peptides were dried on a lyophilizer.

Scheme S1. Solid-phase peptide synthesis of Tet*-Pro$_n$-Acd (n = 2-14) on 2-chlorotrityl resin

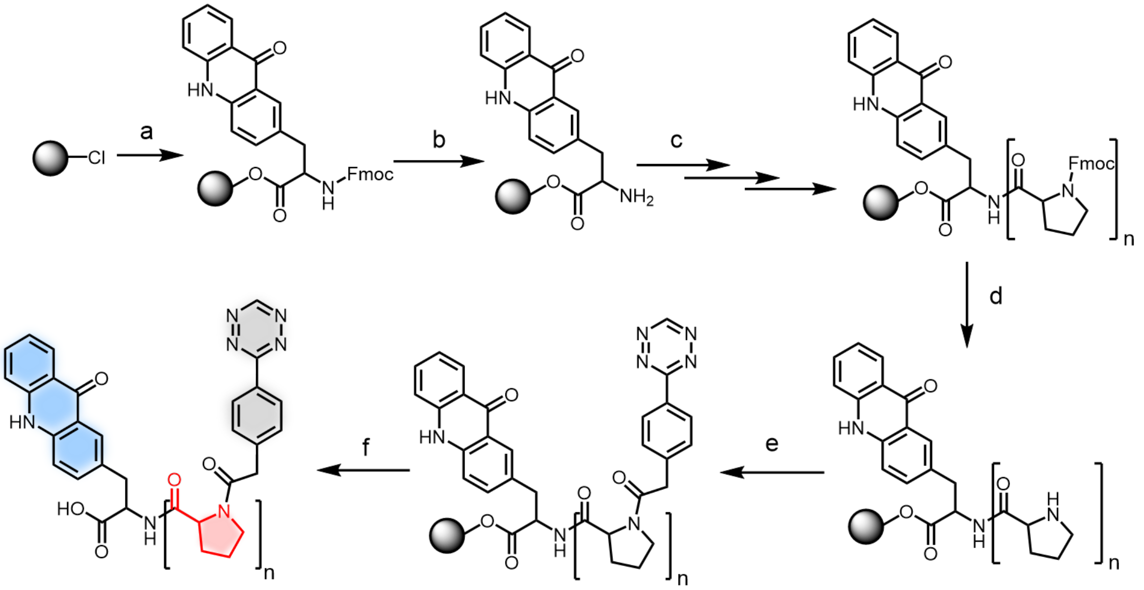

Reagents and conditions: (a) Coupling: Fmoc-Acd-OH, HBTU, and DIPEA in dry DMF; (b) Deprotection: 20 % piperidine in DMF; (c) Coupling: Fmoc-Pro-OH, HBTU, and DIPEA in dry DMF; (d) Deprotection: 20 % piperidine in DMF; (e) Coupling: Tet*-COOH or Fmoc-Leu-OH (control), BOP, and DIPEA in dry DMF (f) Cleavage: 50 % Trifluoroacetic acid in water.

Table S2. Peptide Purification Methods and Retention Times

Peptide	Gradient	Retention Time
Tet*-(Pro)$_2$-Acd	1	34.66
Tet*-(Pro)$_3$-Acd	1	35.92
Tet*-(Pro)$_4$-Acd	1	25.11
Tet*-(Pro)$_5$-Acd	1	26.03
Tet*-(Pro)$_6$-Acd	2	30.74
Tet*-(Pro)$_7$-Acd	2	30.75
Tet*-(Pro)$_8$-Acd	2	31.85
Tet*-(Pro)$_9$-Acd	3	24.10
Tet*-(Pro)$_{10}$-Acd	3	25.33
Tet*-(Pro)$_{11}$-Acd	3	26.79
Tet*-(Pro)$_{12}$-Acd	3	28.12
Tet*-(Pro)$_{13}$-Acd	3	29.69
Tet*-(Pro)$_{14}$-Acd	3	31.23
Leu-(Pro)$_4$-Acd	1	32.19

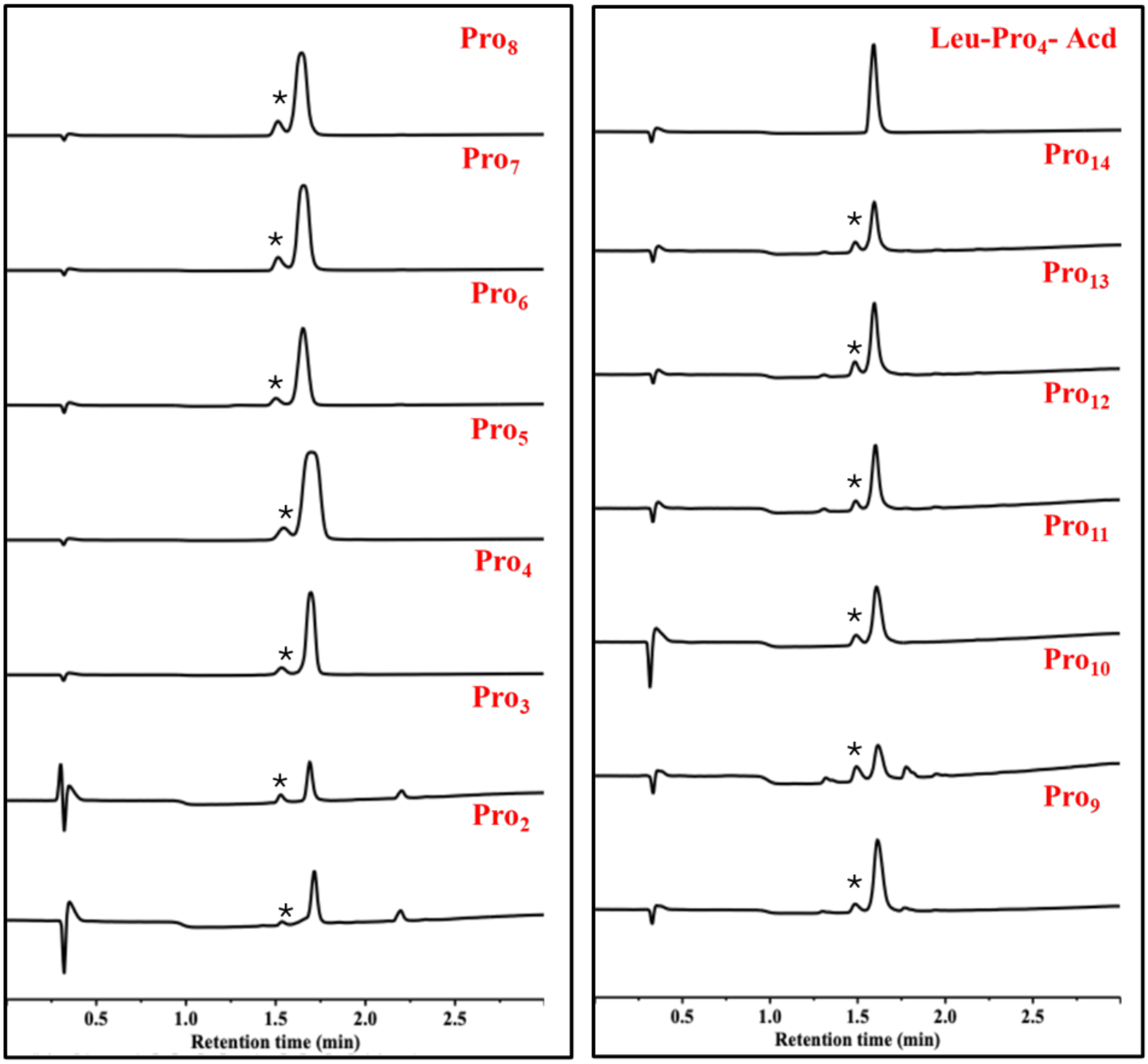

Figure S2. LC-MS chromatograms of purified peptides Tet*-Pro$_n$-Acd (n = 2-14) and Leu-Pro$_4$-Acd with * peak corresponding to the reduced form of Tet*.

Table S3. HPLC Gradients for Peptide Purification

	Time (min)	% B	No.	Time (min)	% B	No.	Time (min)	% B
1	0:00	5	2	0:00	5	3	0:00	5
	5:00	5		5:00	5		5:00	5
	10:00	25		10:00	30		10:00	40
	40:00	55		40:00	60		40:00	70
	45:00	100		45:00	100		45:00	100
	53:00	100		53:00	100		53:00	100
	55:00	5		55:00	5		55:00	5

Table S4. Calculated and Observed Masses of Peptides from LC-MS and MALDI-MS

Peptide	$[M+H]^+$				$[M+Na]^+$	
	Calculated		Observed		Calculated	Observed
	MALDI-MS	LC-MS	MALDI-MS	LC-MS	MALDI-MS	MALDI-MS
Tet*-(Pro)$_2$-Acd	675.26	675.26	675.28	675.28	697.24	697.51
Tet*-(Pro)$_3$-Acd	772.31	772.31	772.47	772.54	794.29	794.48
Tet*-(Pro)$_4$-Acd	869.37	869.37	869.71	869.61	891.35	892.68
Tet*-(Pro)$_5$-Acd	966.42	966.42	966.82	966.11	988.41	988.77
Tet*-(Pro)$_6$-Acd	1063.48	1063.48	1063.81	1063.82	1085.46	1085.81
Tet*-(Pro)$_7$-Acd	1160.53	1160.53	1160.93	1160.63	1182.51	1182.92
Tet*-(Pro)$_8$-Acd	1257.58	1257.58	1257.82	1257.70	1279.57	1279.86
Tet*-(Pro)$_9$-Acd	1354.64	1354.64	1354.76	1354.89	1376.62	1377.93
Tet*-(Pro)$_{10}$-Acd	1451.70	1451.70	1452.54	1452.01	1473.67	1475.27
Tet*-(Pro)$_{11}$-Acd	1548.76	1548.76	1549.44	1549.01	1570.73	1572.50
Tet*-(Pro)$_{12}$-Acd	1645.80	1645.80	1647.12	1646.04	1667.77	1668.84
Tet*-(Pro)$_{13}$-Acd	1742.85	1742.85	1744.30	1743.05	1764.82	1765.91
Tet*-(Pro)$_{14}$-Acd	1839.90	1839.90	1841.27	1840.25	1861.87	1862.39
Leu-(Pro)$_4$-Acd	826.41	826.41	826.86	826.64	848.40	848.92

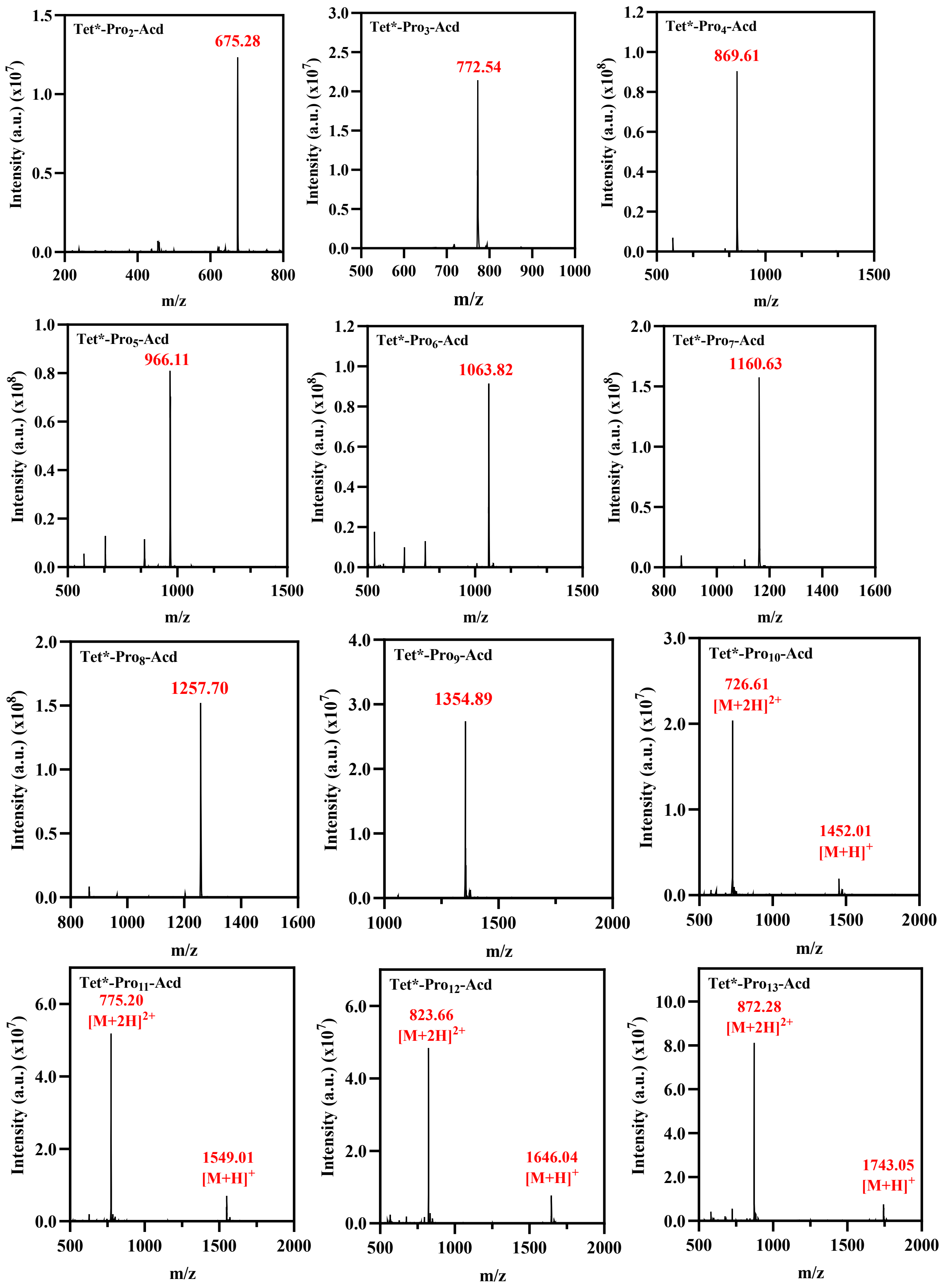
Tet*-Pro$_2$-Acd
675.28
Tet*-Pro$_3$-Acd
772.54
Tet*-Pro$_4$-Acd
869.61
Tet*-Pro$_5$-Acd
966.11
Tet*-Pro$_6$-Acd
1063.82
Tet*-Pro$_7$-Acd
1160.63
Tet*-Pro$_8$-Acd
1257.70
Tet*-Pro$_9$-Acd
1354.89
Tet*-Pro$_{10}$-Acd
726.61
$[M+2H]^{2+}$
1452.01
$[M+H]^{+}$
Tet*-Pro$_{11}$-Acd
775.20
$[M+2H]^{2+}$
1549.01
$[M+H]^{+}$
Tet*-Pro$_{12}$-Acd
823.66
$[M+2H]^{2+}$
1646.04
$[M+H]^{+}$
Tet*-Pro$_{13}$-Acd
872.28
$[M+2H]^{2+}$
1743.05
$[M+H]^{+}$
Intensity (a.u.) (x10^7)
Intensity (a.u.) (x10^8)
m/z

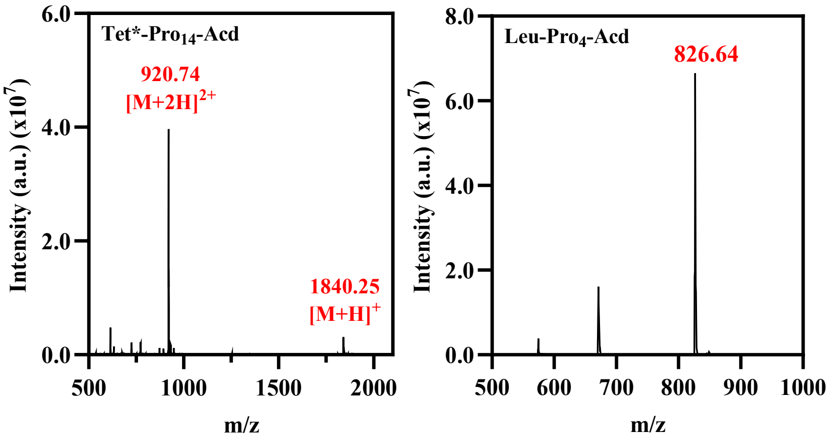

Figure S3. Mass spectrum of purified peptides Tet*-Pro_n-Acd (n = 2-14) and Leu-Pro_4-Acd.

Proline Ruler FRET Measurements

Fluorescence Spectroscopy. Dry proline ruler peptides were diluted in CH_3CN/H_2O (10:90 v/v) and absorbance spectra from 300 to 600 nm were scanned using quartz fluorometer cells with path lengths of 1.00 cm. The peptides were diluted to concentrations of approximately 10 μM in 0.1 M phosphate buffer (57 mM Na_2HPO_4•$7H_2O$, 42 mM $NaH_2PO_4.H_2O$, pH 7.0 adjusted with HCl), as determined by absorbance at 386 nm (ε_{386} = 5700 $M^{-1}cm^{-1}$).[3] Fluorescence spectra for each peptide were collected at 450 nm with an excitation wavelength of 385 nm in triplicate using Tecan Spark plate reader (5 nm bandwidth, 40 μs integration time, 31000 μm Z-position, 4 sec linear shaking) with a nonsterile Greiner black, flat μClear, 384 well low volume microplate. Also, all Acd containing peptides were excited at 385 nm, with emission measured from 400 nm to 600 nm (30 flashes, 5 nm bandwidth). Examples of fluorescence spectra and associated UV spectra are shown in Figure S4.

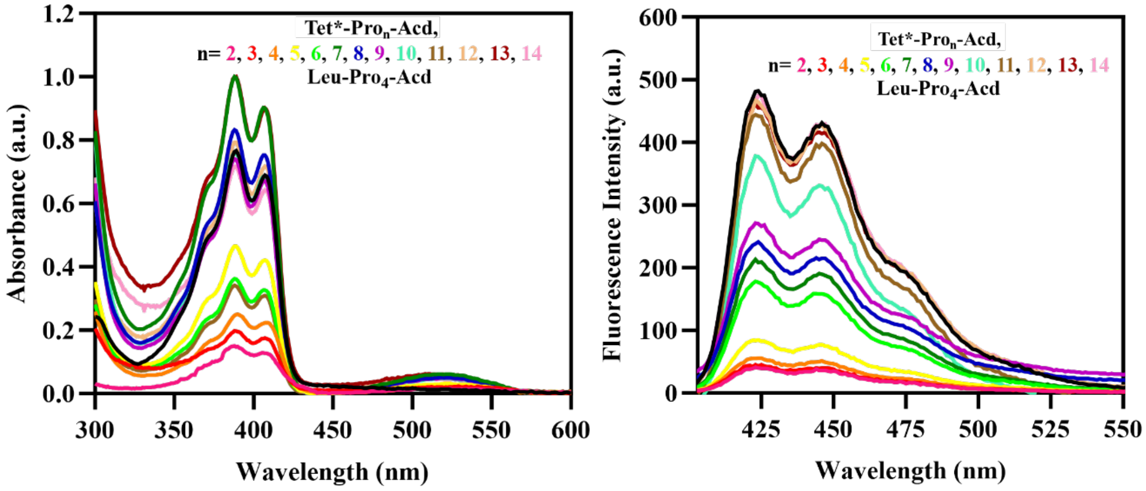

Figure S4. Left: UV absorption spectra collected between 300 and 600 nm. Absorbance is normalized. Right: Fluorescence emission spectra collected between 400 and 600 nm with excitation at 385 nm for Pro Series peptides. Emission spectra are colored according to the corresponding absorption spectrum. The acetonitrile concentration after dilution in 0.1 M phosphate buffer is <1% for all peptides.

Förster Distance Calculation. The Förster distance, R_0, is given in Å by Equation S2[4-5]

$$R_0^6 = \frac{9000(ln10)\kappa^2 Q_D J}{128\pi^5 n^4 N_A} \tag{S2}$$

Where κ^2 is a geometrical factor that relates the orientation of the donor and acceptor transition moments, Q_D is the quantum yield of the donor, n is the index of refraction of the solvent, N_A A is Avogadro's number, and J is the spectral overlap integral defined in units of M^{-1} •cm^{-1} •nm^4 . Combining constants and rearranging gives R_0 as

$$R_0 = 0.211(Q_D \kappa^2 n^{-4} J)^{1/6} \tag{S3}$$

J is formally defined as

$$J = \int_0^\infty f_D(\lambda)\varepsilon_A(\lambda)\lambda^4 \, d\lambda \tag{S4}$$

Where $\varepsilon_A(\lambda)$ is the molar extinction coefficient of the acceptor at each wavelength λ and $f_D(\lambda)$ is the normalized donor emission spectrum given by

$$f_D(\lambda) = \frac{F_{D\lambda}(\lambda)}{\int_0^\infty F_{D\lambda}(\lambda)d(\lambda)} \tag{S5}$$

where $F_{D\lambda}(\lambda)$ is the fluorescence of the donor at each wavelength λ. Fluorescence spectra of Acd in water were integrated with GraphPad Prism (version 10.0.0, GraphPad Software, Boston, Massachusetts, USA) from 400 to 600 nm to calculate $f_D(\lambda)$. UV-Vis spectra of Tet* were used to determine $\varepsilon_A(\lambda)$. The value of ε_{540} = 300 M^{-1} •cm^{-1} was used to calculate J, which was found to be $9.51 \times 10^{12} M^{-1} \cdot cm^{-1} \cdot nm^4$. Substituting this result into Equation S2, as well as 0.95 for the quantum yield of Acd in water,[7] 1.33 for the index of refraction of water, and 2/3 for κ^2 gives the Förster distance of 24 Å for the Tet*-Acd FRET pair.

For the theoretical FRET distance dependence calculation, Equation S6 was used, where R_0 the distance was fixed at 24 Å (~ 8 prolines).

$$\frac{F}{F_0} = \left\{1 - \frac{1}{1+\left(\frac{R}{R_0}\right)^6}\right\} \tag{S6}$$

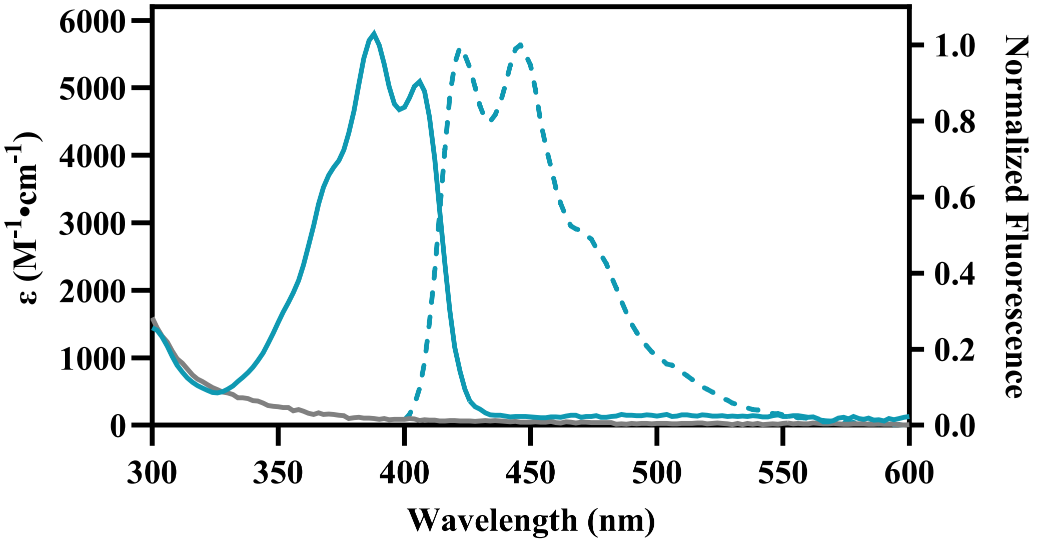

Figure S5. Acd/Tet*-sTCO-OH adduct FRET Quenching. Acd/Tet* spectra showing Acd absorbance (solid blue line) and emission from 400 to 600 nm with excitation at 385 nm (dashed blue line) and Tet*-sTCO-OH adduct absorbance (grey solid line).

For Acd and the Tet*-sTCO-OH adduct spectral overlap calculation, UV-Vis spectra of the Tet*-sTCO-OH adduct were used to determine $\varepsilon_A(\lambda)$ which gave J as $1.99 \times 10^{12} M^{-1} \cdot cm^{-1} \cdot nm^4$. Using this J value, an R_0 of 18 Å can be calculated for Acd/Tet-sTCO FRET.

Dequenching of Proline Ruler Peptides

sTCO-OH reaction with peptides. Fresh 15 μM solutions of each peptide were prepared in 0.1 M phosphate buffer. Fluorescence spectra for each peptide were collected at 450 nm with an excitation wavelength of 385 nm in triplicate using Tecan Spark plate reader (5 nm bandwidth, 40 μs integration time, 31000 μm Z-position, 4 sec linear shaking) with a nonsterile Greiner black, flat μClear, 384 well low volume microplate. 150 μM of sTCO-OH was then added to each peptide well. The mixtures were allowed to react for 15 minutes with shaking (500 rpm) at 37 °C. Again, fluorescence spectra for each peptide were collected at 450 nm with an excitation wavelength of 385 nm under the same conditions as unlabeled peptides.

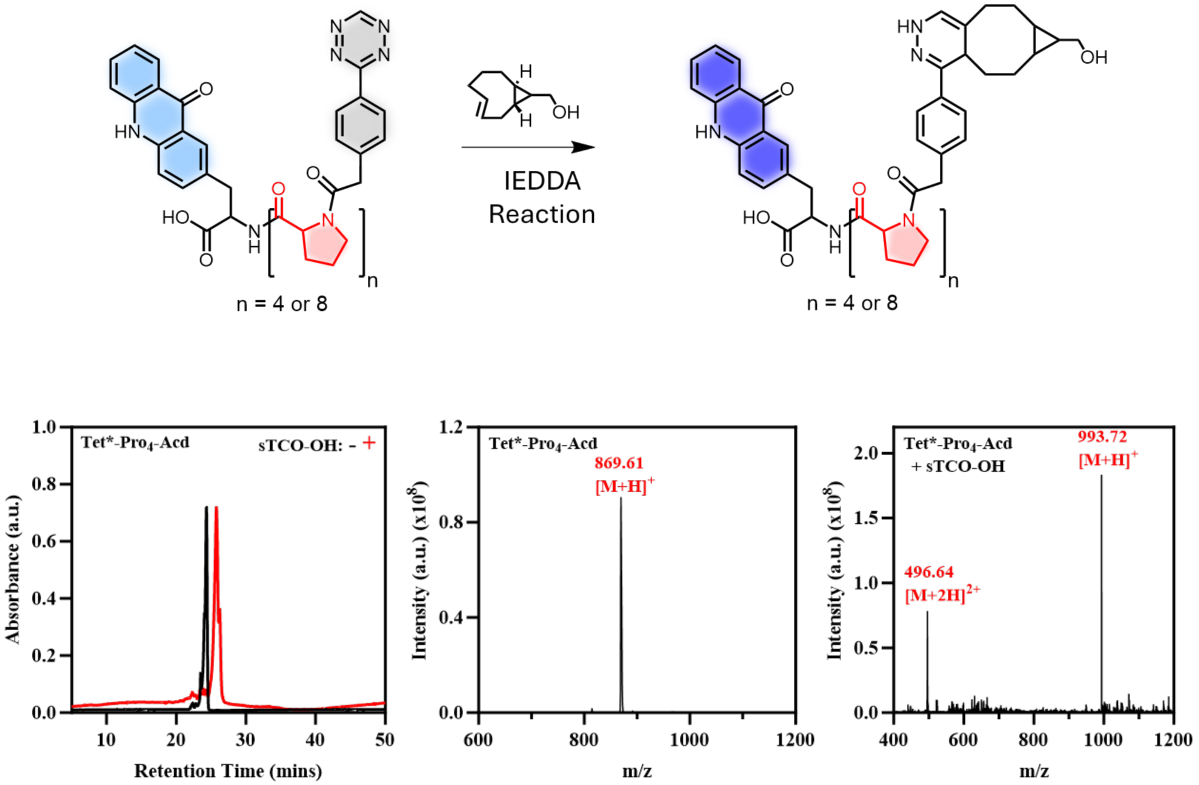

Figure S6. Top: Schematic representation of Tet* labeling with sTCO-OH. Bottom Left: Analytical HPLC Chromatograms of Tet*-Pro_4-Acd before and after reaction with sTCO-OH. Absorbance is normalized. Peak absorption measured at 215 nm. Bottom Middle: MALDI-MS spectra of Tet*-Pro_4-Acd. Bottom Right: MALDI-MS spectra of Tet*-Pro_4-Acd after sTCO-OH labeling.

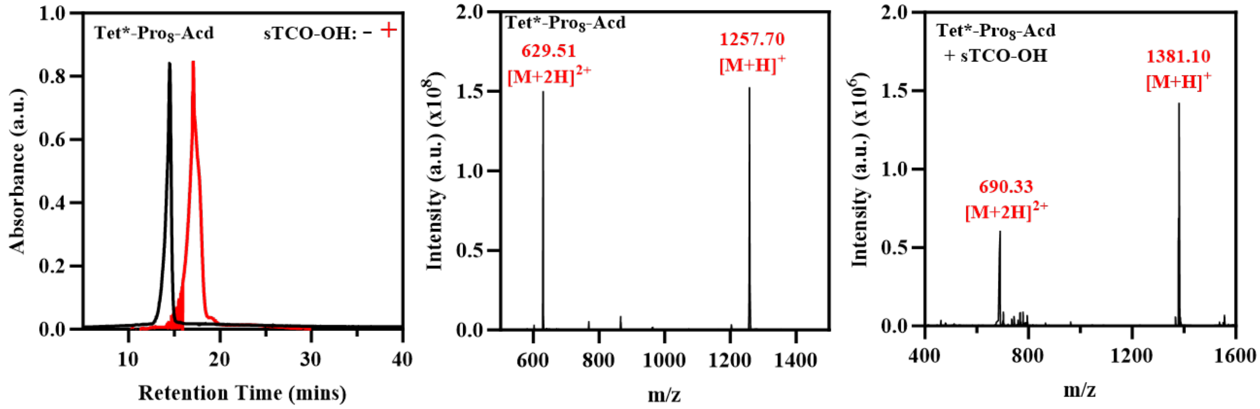

Figure S7. Left: Analytical HPLC Chromatograms of Tet*-Pro$_8$-Acd before and after reaction with sTCO-OH. Peak absorbance is normalized that measured at 215 nm. Middle: MALDI-MS spectra of Tet*-Pro$_8$-Acd. Right: MALDI-MS spectra of Tet*-Pro$_8$-Acd after sTCO-OH labeling.

Photolysis of peptides. 15 μM of Tet* -Pro$_5$-Acd was prepared in 0.1 M phosphate buffer. These peptides were irradiated with UV light of 254 nm under different time intervals (0, 5, 10, 25, 35, 50, 60) and fluorescence spectra were collected at 450 nm with an excitation wavelength of 385 nm in triplicate using Tecan Spark plate reader under the same conditions as described before.

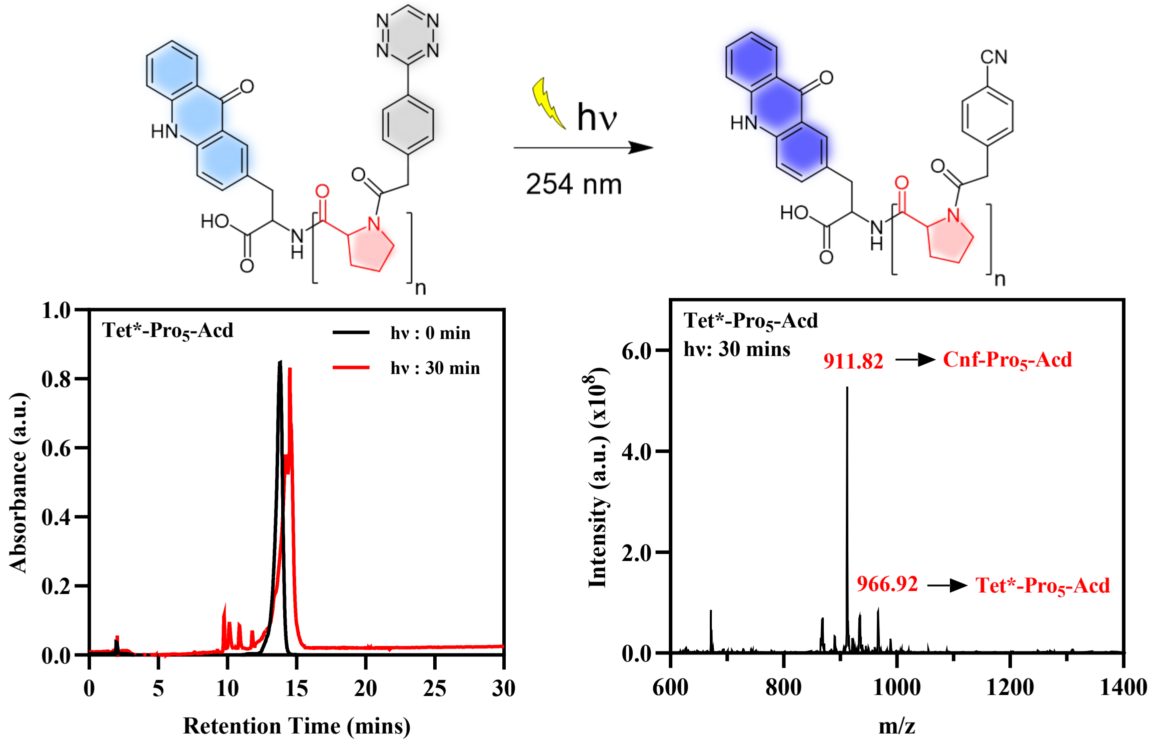

Figure S8. Top: Schematic representation of Tet* photolysis at 254 nm leading to dequenching of Acd fluorescence *via* formation of Cnf. Bottom Left: Analytical HPLC Chromatograms of Tet*-Pro$_5$-Acd before and after irradiation. Absorbance is normalized. Peak absorbance measured at 215 nm. Bottom Right: MALDI-MS spectra of Tet*-Pro$_5$-Acd after 30 minutes of irradiation.

Cloning of Dual Label Plasmids: CaM, LexA, and RecA

Previously Cloned constructs. The gene containing full-length calmodulin (CaM) was previously cloned into the pTXB1 vector containing a C-terminal MxeGyrA intein, followed by a His_6 purification tag.[8] The TAG codon for Acd incorporation at position 113 via amber stop codon suppression was previously inserted using QuikChange® PCR, yielding the pTXB1-CaM-TAG_{113}-GyrA-His_6 plasmid.[9]

Cloning of CaM plasmid. All PCR reactions were carried out on a T100 thermocycler from Bio-Rad (Hercules, CA, USA). DNA concentrations were determined with a Tecan Spark plate reader. Agarose gels were visualized on G:Box mini (Syngene).

To incorporate Tet at position 93 of CaM, PCR was performed on the pTXB1-CaM-TAG_{113}-GyrA-His_6 plasmid using the NEB Q5 Hot Start HiFi Master Mix to mutate the Phe codon to TAA (primers for pTXB1CaM-TAA_{93}-TAG_{113}-GyrA-His_6, Table S6). The resulting DNA was transformed into DH5α cells (NEB) and purified using the Miniprep kit (Qiagen).

Cloning of LexA dual-label plasmids. To make pET41-LexA-TAA_{81}-TAG_{86}-His, the original TAA stop codon from pET41-LexA-TAG_{86}-His (reported by Hostetler et al.[12]) was first mutated to TGA using cassette mutagenesis. Briefly, the parent plasmid (pET41-LexA-TAG_{86}-His) was digested using AvrII and XhoI (NEB). Digest was assessed by agarose gel, and the desired fragment was obtained by gel purification (Monarch DNA Gel Extraction Kit, NEB). An oligo pair (Table S6) containing the mutation was annealed, phosphorylated using T4 PNK (NEB) in a T100 Thermal Cycler (BioRad), and ligation was completed using T4 DNA ligase (NEB) with overnight cycling ligation. The resulting ligation mixture and negative control were transformed into NEB Turbo competent cells. Plasmid was obtained by miniprep (Qiagen). The mutation was confirmed by Sanger sequencing (Genewiz by Azenta). A second round of cassette mutagenesis was used to mutate the R81 codon to TAA. This cloning was accomplished as described above with the following alterations: a different oligo pair (see Table S6 below) was used to install the TAA mutation, and the pET41-LexA-TAG_{86}-His (stop TGA) parent plasmid was digested using SpeI-HF and BmtI-HF (NEB).

For pET41-LexA-TAG_{81}-TAA_{86}-His, pET41-LexA-TAA_{86}-TAG_{175}-QM-His, and pET41-LexA-TAA_{86}-TAG_{175}-K156A-His, the pET41-LexA-TAG_{86}-His (stop TGA) parent plasmid was digested using SpeI-HF and HindIII (NEB). Digestion was assessed by agarose gel, and the desired fragment was obtained by gel purification (Monarch DNA Gel Extraction Kit, NEB). Gene fragments containing the desired mutations with flanking SpeI and HindIII restriction enzyme sites were synthesized (Twist Bioscience) (see Table S5 below). The gene fragments were digested with SpeI-HF and HindIII (NEB) and purified by NucleoSpin Gel & PCR Clean-Up Kit (Macherey-Nagel). Ligation was completed using T4 DNA ligase (NEB) with overnight cycling ligation. The resulting ligation mixture and negative control were transformed into NEB Turbo competent cells. Plasmid was obtained by miniprep (Qiagen). The constructs were confirmed by Sanger sequencing (Genewiz by Azenta).

For pET41-LexA-TAG_{175}-QM-His, the parent plasmid was pET41-LexA-TAA_{86}-TAG_{175}_QM-His. Cassette mutagenesis was used as described above with the following alterations: a different oligo pair (see Table S6 below) was used to revert the TAA mutation to the E86 codon, the oligo pair was annealed, but not phosphorylated, and the parent plasmid was digested using BamHI-

HF and EagI-HF (NEB). Additionally, the vector was purified using the NucleoSpin Gel & PCR Clean-Up Kit (Macherey-Nagel). The construct was confirmed by whole-plasmid sequencing (Plasmidsaurus).

For pET41-LexA-TAG_{175}-K156A-His, the parent plasmid was pET41- LexA-TAA_{86}-TAG_{175}-K156A-His. Cassette mutagenesis was used as described above with the following alterations: a different oligo pair (see Table S6 below) was used to revert the TAA mutation to the E86 codon, and the parent plasmid was digested using BamHI-HF and EagI-HF (NEB). Additionally, the vector was purified using the NucleoSpin Gel & PCR Clean-Up Kit (Macherey-Nagel). The construct was confirmed by whole-plasmid sequencing (Plasmidsaurus).

Cloning of RecA dual-label plasmid. To make the RecA dual label construct plasmid, a gene fragment containing the sequence of the RecA gene with the desired mutations (codon for R33 changed to TAA and codon for I102 changed to TAG) and flanking SphI and AvrII restriction enzyme sites was synthesized (Twist Bioscience) (see Table S5 below). Both the parent plasmid (pET41-RecA-WT-His)[17] and gene fragment were digested with SphI-HF and AvrII (NEB). For the vector, digestion was assessed by agarose gel, and the desired fragment was obtained by gel purification (Monarch DNA Gel Extraction Kit, NEB). For the insert, the digested gene fragment was purified by NucleoSpin Gel & PCR Clean-Up Kit (Macherey-Nagel). Ligation was completed using T4 DNA ligase (NEB) with overnight cycling ligation. The resulting ligation mixture and negative control were transformed into NEB Turbo competent cells. Plasmid was obtained by miniprep (Qiagen). The construct was confirmed by whole-plasmid sequencing (Plasmidsaurus).

Table S5: Gene fragments used for plasmid cloning

**Construct**	**Gene Fragment Sequence (5'-3')**
pET41-LexA-TAG_{81}-TAA_{86}-His	AGAAGGGTTGCCACTAGTAGGTtagGTGGCGGCCGGTtaaCCACTGCTAGC GCAACAGCATATTGAAGGTCATTACCAGGTGGATCCTTCCTTATTCAAGC CGAATGCTGATTTCCTGCTGCGCGTCAGCGGGATGTCGATGAAAGATATT GGCATTATGGATGGAGATCTGCTGGCAGTGCATAAAACTCAGGACGTCC GTAACGGTCAGGTCGTTGTCGCACGTATTGATGACGAAGTTACCGTTAAA CGTTTGAAAAAACAGGGCAATAAAGTCGAGCTCTTGCCAGAAAATTCCGA GTTTAAACCAATTGTCGTTGACCTTCGTCAGCAAAGCTTCACCATTGAAG G
pET41-LexA-TAA_{86}-TAG_{175}-QM-His	AGAAGGGTTGCCACTAGTAGGTCGTGTGGCGGCCGGTtaaCCACTGccaG CGCAAtggCATATTGAAGGTCATTACCAGGTGGATCCTTCCTTATTCAAGC CGAATGCTGATTTCCTGCTGCGCGTCAGCGGGATGTCGATGAAAGATATT GGCATTATGGATGGAGATCTGCTGGCAGTGCATAAAACTCAGGACGTCC GTAACGGTCAGGTCGTTGTCGCACGTATTGATGACgcaGTTACCGTTgcaC GTTTGAAAAAACAGGGCAATAAAGTCGAGCTCTTGCCAGAAAATTCCGAG TTTtagCCAATTGTCGTTGACCTTCGTCAGCAAAGCTTCACCATTGAAGG
pET41-LexA-TAA_{86}-TAG_{175}-K156A-His	AGAAGGGTTGCCACTAGTAGGTCGTGTGGCGGCCGGTtaaCCACTGCTAG CGCAACAGCATATTGAAGGTCATTACCAGGTGGATCCTTCCTTATTCAAG CCGAATGCTGATTTCCTGCTGCGCGTCAGCGGGATGTCGATGAAAGATAT TGGCATTATGGATGGAGATCTGCTGGCAGTGCATAAAACTCAGGACGTCC GTAACGGTCAGGTCGTTGTCGCACGTATTGATGACGAAGTTACCGTTgca CGTTTGAAAAAACAGGGCAATAAAGTCGAGCTCTTGCCAGAAAATTCCGA GTTTtagCCAATTGTCGTTGACCTTCGTCAGCAAAGCTTCACCATTGAAGG
pET41-RecA-TAA_{33}-TAG_{102}-His	ccgccgccgcaaggaatggtgcatgcaaggagatggcgcccaacagtcccccggccacggggcctgcc accatacccacgccgaaacaagcgctcatgagcccgaagtggcgagcccgatcttccccatcggtgatgt cggcgatataggcgccagcaaccgcacctgtggcgccggtgatgccggccacgatgcgtccggcgtaga

	ggatcgagatcgatctcgatcccgcgaaattaatacgactcactataggggaattgtgagcggataacaatt<br>cccctctagaaataattttgtttaactttaagaaggagatatacgatgGCTATCGACGAAAACAAA<br>CAGAAAGCGTTGGCGGCAGCACTGGGCCAGATTGAGAAACAATTTGGTA<br>AAGGCTCCATCATGCGCCTGGGTGAAGACtaaTCCATGGATGTGGAAACC<br>ATCTCTACCGGTTCGCTTTCACTGGATATCGCGCTTGGGGCAGGTGGTCT<br>GCCGATGGGCCGTATCGTCGAAATCTACGGACCGGAATCTTCCGGTAAA<br>ACCACGCTGACGCTGCAGGTGATCGCCGCAGCGCAGCGTGAAGGTAAAA<br>CCTGTGCGTTTATCGATGCTGAACACGCGCTGGACCCAtagTACGCACGT<br>AAACTGGGCGTCGATATCGACAACCTGCTGTGCTCCCAGCCGGACACCG<br>GCGAGCAAGCTTTGGAAATCTGTGACGCCCTGGCGCGTTCTGGCGCAGT<br>AGACGTTATCGTCGTTGACTCCGTGGCGGCACTGACGCCGAAAGCGGAA<br>ATCGAAGGCGAAATCGGCGACTCTCAtATGGGCCTTGCGGCACGTATGAT<br>GAGCCAGGCGATGCGTAAGCTGGCGGGTAACCTTAAGCAGTCCAACACG<br>CTGCTGATCTTCATCAACCAGATCCGTATGAAAATTGGTGTGATGTTCGG<br>TAACCCGGAAACCACTACCGGTGGTAACGCGCTGAAATTCTACGCTAGC<br>GTTCGTCTCGACATCCGTCGTATCGGCGCGGTGAAAGAGGGCGAAAACG<br>TGGTGGGTAGCGAAACCCGCGTGAAAGTGGTGAAGAACAAAATCGCTGC<br>GCCGTTTAAACAGGCTGAATTCCAGATCCTCTACGGCGAAGGTATCAACT<br>TCTACGGCGAACTGGTTGACCTGGGCGTAAAAGAGAAGCTGATCGAGAA<br>AGCAGGCGCGTGGTACAGCTACAAAGGTGAGAAGATCGGTCAGGGTAAA<br>GCGAATGCGACTGCCTGGCTGAAAGATAACCCGGAAACCGCGAAAGAGA<br>TCGAGAAGAAAGTACGTGAGCTCCTGCTGAGCAACCCGAACTCAACGCC<br>GGATTTCTCTGTAGATGATAGCGAAGGCGTAGCAGAAACTAACGAAGATT<br>TTCACCACCATCACCATCATCACCACTgATTGATTAATACCTAGGcaaggctg<br>ctaaacaaagcc

Cloning of Synthetase plasmids:

pULTRAI-Tet3.0 RS[TAA] was cloned as previously described (Addgene #164580).[13] To create pEVOL-AcdA9 RS[TAG], pEVOL-pAzF[TAG] (Addgene # #31186) was first digested with BglII and PstI.[14] Three PCR amplifications were performed, two of which used pBK-*Mj*-Acd A9[15] as a template to amplify two copies of the *Mj* Acd A9 RS genes with TGA stop codons and the appropriate homology ends for directional recombination into the two RS open reading frames of pEVOL (primers pEVOL-Mj-aaRS1-fwd and pEVOL-Mj-aaRS1(TAA>TGA)-rev; pEVOL-Mj-aaRS2-fwd and pEVOL-Mj-aaRS2(TAA>TGA)-rev). The third PCR reaction amplified the fragment connecting the two RS open reading frames from pEVOL-pAzF[TAG], using primers pEVOL-inter-aaRS-fwd and pEVOL-inter-aaRS-rev. These three PCR fragments and the BglII/PstI digested pEVOL-pAzF[TAG] backbone were gel purified and extracted using a Gel Extraction Kit (Macherey-Nagel). The Seamless Ligation Cloning Extract (SLiCE) cloning method[16] was used to recombine these three PCR fragments with the BglII/PstI digested pEVOL-pAzF[TAG] vector simultaneously. The resulting DNA was transformed into chemically competent DH10b cells and purified using a NucleoSpin Plasmid kit (Macherey-Nagel), and the plasmid sequence was confirmed using Sanger sequencing (GeneWiz by Azenta).

Primers:

Primer	Sequence
pEVOL-Mj-aaRS1-fwd	5' TGGGCTAACAGGAGGAATTAGATCTATGGACGAATTTGAAATGATAAAGAGAAACAC 3'
pEVOL-Mj-aaRS1(TAA>TGA)-rev	5' CCAAGCTGGAGACCGTTTAAACTCATCATAATCTCTTTCTAATTGGCTCTAAAATCTTTATAAGTTC 3'
pEVOL-Mj-aaRS2-fwd	5' TTGTTTACGCTTTGAGGAATCCCATATGGACGAATTTGAAATGATAAAGAGAAACA 3'
pEVOL-Mj-aaRS2(TAA>TGA)-rev	5' GGCAATTTAGCGTTTGAAACTGCAGTCATAATCTCTTTCTAATTGGCTCTAAAATCTTTATAAGTTCTTC 3'
pEVOL-inter-aaRS-fwd	5' TGAGTTTAAACGGTCTCCAGCTTGG 3'
pEVOL-inter-aaRS-rev	5' ATGGGATTCCTCAAAGCGTAAACAA 3'

Synthetase plasmids and their maps used in this study:

Synthetase plasmid	origin of replication	GCE platform	resistance
pULTRAI-**Tet3.0** RS[TAA]	CDF	*M. barkeri*	Streptomycin
pEVOL-**Acd**A9 RS[TAG]	p15A	*M. janaschii*	Chloramphenicol

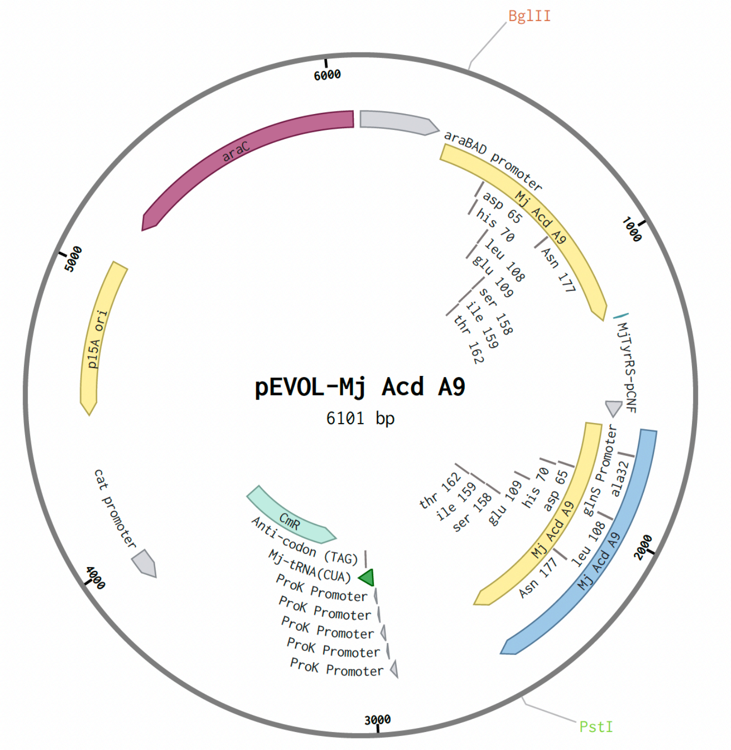

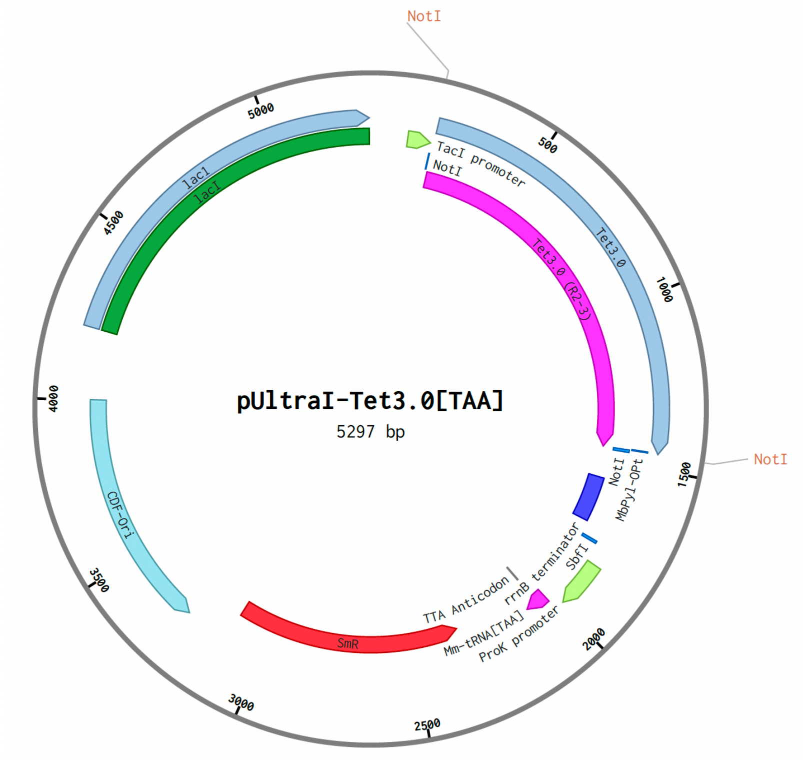

Plasmids used in this study:

Plasmid	Resistance
pTXB1-CaM-TAA$_{93}$-TAG$_{113}$-GyrA-His$_6$	Ampicillin
pET41-LexA-TAA$_{81}$-TAG$_{86}$-His	Kanamycin
pET41-LexA-TAG$_{81}$-TAA$_{86}$-His	Kanamycin
pET41-LexA-TAA$_{86}$-TAG$_{175}$-QM-His	Kanamycin
pET41-LexA-TAA$_{86}$-TAG$_{175}$-K156A-His	Kanamycin
pET41-LexA-TAG$_{175}$-QM-His	Kanamycin
pET41-LexA-TAG$_{175}$-K156A-His	Kanamycin
pET41-RecA-TAA$_{33}$-TAG$_{102}$-His	Kanamycin

Table S6. Primers used for PCR mutations and cassette mutagenesis

Primer	Sequence	T_m (˚C)
CaM(F93TAA) FWD	5' GTGTAAGACAAGGATGGTAATG 3'	61
CaM(F93TAA) REV	5' ACGGAACGCTTCTCTAATTT 3'	
LexA(TAA to TGA stop) FWD	5'TCGAGCACCACCACCACCACCACCACCACTGA TTGATTAATAC 3'	
LexA(TAA to TGA stop) REV	5'CTAGGTATTAATCAATCAGTGGTGGTGGTGGTG GTGGTGGTGC 3'	
LexA(R81 codon to TAA) FWD	5'CTAGTAGGTTAAGTGGCGGCCGGTTAGCCACT GCTAG 3'	
LexA(R81 codon to TAA) REV	5' CAGTGGCTAACCGGCCGCCACTTAACCTA 3'	
LexA(TAA to E86 codon) FWD (to make insert for pET41-LexA-TAG$_{175}$-QM-His)	5' GGC CGG TGA ACC ACT GCC TGC GCA ATG GCA TAT TGA AGG TCA TTA CCA GGT G 3'	
LexA(TAA to E86 codon) REV (to make insert for pET41-LexA-TAG$_{175}$-QM-His)	5' GAT CCA CCT GGT AAT GAC CTT CAA TAT GCC ATT GCG CAG GCA GTG GTT CAC C 3'	
LexA(TAA to E86 codon) FWD (to make insert for pET41-LexA-TAG$_{175}$-K156A-His)	5' GGC CGG TGA ACC ACT GCT TGC GCA ACA GCA TAT TGA AGG TCA TTA CCA GGT G 3'	
LexA(TAA to E86 codon) REV (to make insert for pET41-LexA-TAG$_{175}$-K156A-His)	5' GAT CCA CCT GGT AAT GAC CTT CAA TAT GCT GTT GCG CAA GCA GTG GTT CAC C 3'	

Tm: Calculated annealing temperature

Reagents and Buffers for Bacterial Culture

The following is a general protocol for preparing media for dual ncAA incorporation during protein expression.

Preparation of starter culture media: non-inducing media (NIM). Each component in the table below should be prepared separately and sterilized separately. Once sterilized, each component can be stored separately at room temperature indefinitely, provided sterility is maintained. Components should only be mixed when at room temperature and immediately before use.

	For 500 mL
Aspartate (5%, pH 7.5)	25 mL
18 amino acid mix (25x)	20 mL
1 M $MgSO_4$	1 mL
25x M-Salts	20 mL
40% (w/v) α-D-glucose	6.25 mL
Trace Metals solution (5,000x)*	100 µl
Sterile water (to 500 mL)	428 mL

Expression media: auto-inducing media (AIM). Each component in the table below should be prepared separately and sterilized separately. Once sterilized, each component can be stored separately at room temperature indefinitely, provided sterility is maintained. *Components should only be mixed when at room temperature and immediately before use*. Note that recipes for all components except the 50x 5052 solution and 20% (w/v) arabinose are in the above section above describing "starter culture media".

	For 500 mL
Aspartate (5%, pH 7.5)	25 mL
18 amino acid mix (25x)	20 mL
1 M $MgSO_4$	1 mL
25x M-Salts	20 mL
50x 5052 solution	10 mL
20% (w/v) arabinose	1.25 mL
Trace Metals solution (5,000x)*	100 µL
Sterile water (to 500 mL)	423 mL

Preparation of 18 amino acid mix (25x). Combine the following a total volume of 1 liter. Sterile filter after all amino acids is dissolved. Aliquot into sterile 100-mL bottles or 50 mL conical vials. Stable at 4°C for years w/o contamination, or at -20°C. Final concentration in media for each amino acid is 200 µg/mL.

	MW	mass (g)
Glutamic acid, Na salt	169.1	5
Aspartic acid	133.1	5

Lysine-HCl	182.6	5
Arginine-HCl	210.7	5
Histidine-HCl-H_2O	209.6	5
Alanine	89.1	5
Proline	115.1	5
Glycine	75.1	5
Threonine	119.1	5
Serine	105.1	5
Glutamine	146.1	5
Asparagine-H_2O	150.1	5
Valine	117.1	5
Leucine	131.2	5
Isoleucine	131.2	5
Phenylalanine	165.2	5
Tryptophan	204.2	5
Methionine	149.2	5

Preparation of 25x M-salts. To prepare the solution, dissolve 88.73 g of anhydrous sodium dibasic (Na_2HPO_4) and 85.05 g of anhydrous potassium monobasic (KH_2PO_4) in 1 liter of water to achieve 0.625 M concentrations for each phosphate salt. Next, add 66.86 g of NH_4Cl to obtain a 1.25 M solution, followed by 17.75 g of anhydrous sodium sulfate (Na_2SO_4) for a 0.125 M solution. Do not adjust the pH of the solution. Sterilize the final solution by autoclaving.

Preparation of 5000x trace metals. The following metals are mixed in the ratios mentioned below to prepare the desired 5000x trace metal solution, which is then sterile filtered.

Amount for 30 ml stock solution	Amount of 30 ml stock solution for 50 ml (5,000x) solution	1x media concentration
$CaCl_2 \cdot 2H_2O$ (8.82 g)	500 µl	4 µM
$MnCl_2 \cdot 4H_2O$ (5.93 g)	500 µl	2 µM
$ZnSO_4 \cdot 7H_2O$ (8.62 g)	500 µl	2 µM
$CoCl_2 \cdot 6H_2O$ (1.32 g)	500 µl	0.4 µM
$CuCl_2$ (807 mg)	500 µl	0.4 µM
$NiCl_2$ (777 mg)	500 µl	0.4 µM
$Na_2MoO_4 \cdot 2H_2O$ (1.45 g)	500 µl	0.4 µM
Na_2SeO_3 (1.03 g)	500 µl	0.4 µM
H_3BO_3 (371 mg)	500 µl	0.4 µM
$FeCl_3$ (486 mg)	25 ml	10 µM
	Dilute to 50 ml with sterile water	

Preparation of 50 x 5052 solution for 500 mL. To prepare the solution, dissolve 12.5 g of α-D-glucose (2.5% w/v) and 50 g of lactose (10% w/v) in an appropriate volume of Milli-Q (MQ) water. For the glycerol component (25% v/v), either directly add 125 mL of 100% glycerol or prepare a 50% glycerol solution by adding 250 mL of MQ water to a 500 mL graduated cylinder, placing a clean stir bar in the cylinder, and stirring the water while slowly adding 250 mL of 100% glycerol until the total volume reaches 500 mL. Stir for 5–10 minutes to ensure thorough mixing. Note that the lactose may not dissolve immediately; heat the solution in a microwave for 2–3 minutes to dissolve the lactose fully, ensuring it remains in solution before autoclaving. Once all components are dissolved and mixed, autoclave the solution.

Overexpression of CaM, RecA, and LexA with Tet and Acd

Triple plasmid transformation of CaM. Prepare 1.7 mL Eppendorf tubes containing 1–1.5 µL (~50–200 ng) each of pEVOL-Acd A9 RS[TAG], pULTRAI-Tet3.0 RS[TAA], and the desired pTXB1CaM-TAA$_{93}$-TAG$_{113}$-GyrA-His$_6$ plasmid with a C-terminal intein-His$_6$ fusion gene bearing the amber stop codon and ochre stop codon at the desired position for Acd and Tet incorporation. Thaw 50 µL aliquots of electrocompetent BL21(DE3) cells, gently mix the cells with the plasmids by pipetting, and transfer the mixture into pre-chilled 1 mM electro-cuvettes. Electroporate according to instrument settings, immediately resuspend the cells in 1 mL SOC media, transfer to Eppendorf tubes, and incubate at 37°C with shaking at 250 rpm for 90 minutes. Cells transformed using electroporation were grown on LB/agar plates containing triple antibiotics: for for CaM, 25 µg/mL Chlor, 100 µg/mL Strep, and 50 µg/mL ampicillin (Amp).

Overexpression of CaM. For the starter culture, an individual colony was seeded into 5 mL of non-inducing media (NIM) and grown overnight with shaking at 250 rpm at 37°C. Subsequently, 1% of the NIM culture was inoculated into 100 mL of auto-inducing media (AIM) containing 25 µg/mL chloramphenicol, 100 µg/mL streptomycin, and 50 µg/mL ampicillin, and the culture was grown at 37 °C. After 1 hour and 30 minutes of incubation, non-canonical amino acids such as Tet•HCl (0.5 mM final) dissolved in dry DMF and Acd (0.5 mM final) dissolved in water (solubilized by adding an equimolar amount of NaOH from a 5 M stock) were added to the media. The culture was grown until reaching an optical density (OD600) of 1.5 at 37 °C, followed by overnight incubation with shaking at 250 rpm at varied temperatures of 18 °C, 25 °C, or 30 °C.

Cell lysis and protein purification. The following day, cells were harvested by centrifugation at 4 °C for 20 min at 2740 RCF (Sorvall GS3 rotor). Pellets were then re-suspended in 15 mL of 40 mM Tris at pH 8.3 for a 100 mL culture that was supplemented with EDTA-free protease inhibitor tablets (Pierce Biotechnology; Waltham, MA, USA) and transferred to a metal cup for sonication. Cells were lysed by sonication on ice with a Q700 probe sonicator (QSonica LLC; Newtown, CT, USA) with the following settings: Amplitude 50, Process Time 5-6 min, Pulse-ON Time 1 s, Pulse-OFF Time 2 s. Crude lysate was then transferred to 50 mL centrifugation tubes and clarified via centrifugation at 23400 RCF for 30 min (Sorvall SS34 rotor). Following centrifugation, the supernatant was removed and transferred to a 50 mL Falcon tube. Then, 5 mL of nickel agarose resin (GoldBio; St. Louis, MO, USA) was added, and the lysate-nickel mixture was incubated with nutation at 4 °C for 1 h. The lysate-nickel mixture was then poured into a 20 mL fritted column, and the flow-through was saved. The remaining resin was then washed with ~20 mL Wash Buffer 1 (50 mM HEPES buffer, pH 7.5), ~20 mL Wash Buffer 2 (50 mM HEPES, 5 mM imidazole, pH 7.5), and eluted with 12 mL Elution Buffer (50 mM HEPES, 300mM imidazole, pH 7.5). The resulting elution was dialyzed against 20 mM Tris, pH 8.0, for 4 h to get rid of the excess imidazole. Then β-mercaptoethanol (Bio-Rad Laboratories; Hercules, CA, USA) was added to crude lysate (200 mM final concentration), and the mixture was allowed to incubate with nutation at room temperature overnight. The resulting cleaved protein was dialyzed against 20 mM Tris, pH 8.0, for 8-10 h. The resulting dialysate was then treated with 5 mL nickel agarose resin and incubated with nutation at 4 °C for 1 h. The mixture was then applied to a 20 mL fritted column, and flow-through containing CaM was collected in a 15 mL Falcon tube. The resulting enriched protein

mixture was then purified via FPLC using a 5 mL HiTrap Q-HP column (Cytiva; Marlborough, MA, USA) using the following method: Buffer A: 20 mM Tris, pH 8.0; Buffer B: 20 mM Tris, 1 M NaCl, pH 8.0; Gradient: 0% Buffer B – 5 column volumes, 0 – 10% Buffer B – 5 column volumes, 20-30% Buffer B – 20 column volumes, 30-100% Buffer B – 10 column volumes; flow rate 3 mL/min. The resulting fractions were then assessed for purity via MALDI MS, and pure fractions were combined. Protein was then concentrated, and buffer exchanged into 20 mM Tris pH 8.0 to a final concentration of 100-200 μM via Amicon 3 kDa MWCO filters (Millipore Sigma; St. Louis, MO, USA). Purified protein was aliquoted into 1.5 mL tubes and stored at -80 °C until further use.

Overexpression of RecA mutant: In a 1.7 mL eppendorf tube 50 μL aliquot of electrocompetent BL21(DE3) cells were transformed with three plasmids 1–1.5 μL (~50–200 ng) each, one containing the recombinant RecA gene bearing the amber stop codon and ochre stop codon at the desired position for Acd and Tet incorporation respectively, and the other two encoding the aminoacyl tRNA synthetase with improved specificity for Acd and Tet incorporation. The mixture was then transferred into pre-chilled 1 mM electro-cuvettes. After electroporation according to the instrument settings, the cells were immediately resuspended in 1 mL SOC media, transferred to an Eppendorf tube, and incubated at 37 °C with shaking at 250 rpm for 90 minutes. Cells transformed using electroporation were grown on LB/agar plates at 37 °C overnight with 25 μg/mL chloramphenicol, 100 μg/mL streptomycin, and 50 μg/mL kanamycin as required antibiotics. For starter culture, an individual colony was seeded into 5 mL of non-inducing media (NIM) with selective antibiotics and grown at 37°C overnight with shaking at ~250 rpm. To scale up expressions, ~1% inoculum (2 mL) of NIM culture was added to 200 mL of auto-induction media in 1 L baffled flasks with selective antibiotics and grown at 37 °C. After ~1-2 hours, solubilized Acd and Tet were added to a final concentration of 0.5 mM, and continued growth at 37 °C until OD_{600} was ~1.5, and then the temperature was reduced to 30 °C. After growth for 20 hours, cells were harvested and stored at -20 °C.

Cell lysis and protein purification: Cells were resuspended in lysis buffer containing 50 mM sodium phosphate, pH 8.0, 500 mM KCl, 500 mM NaCl, 25 mM imidazole, 250 U/mL benzonase nuclease (Sigma), and 0.25 mg/mL lysozyme (Sigma); cells were then lysed using a sonicator. Lysates were then centrifuged (Sorvall SS34 rotor) for 30 mins at 26900 RCF. Clarified lysates were allowed to flow over HisPur cobalt resin (Thermo Fisher) pre-equilibrated with the respective lysis buffer. The resin was then washed with 20 column volumes of RecA cobalt wash buffer (50 mM sodium phosphate pH 8.0, 500 mM KCl, 500 mM NaCl, 100 mM imidazole), followed by elution in 3 column volumes of RecA cobalt elution buffer (50 mM sodium phosphate, pH 8.0, 500 mM KCl, 500 mM NaCl, 400 mM imidazole). After HisPur cobalt resin, for further purification, RecA samples were precipitated by incubation for 30 min at 4 °C using 48% (final) saturation ammonium sulfate, and the pellet was retained after spinning at 3,000 rcf for 30 min. Pellets were resuspended in RecA heparin load/wash buffer (20 mM Tris–HCl, pH 8.0, 20 mM NaCl, 2 mM tris(2-carboxyethyl)phosphine (TCEP)) and dialyzed overnight at 4 °C against the same buffer to remove residual ammonium sulfate. All samples were loaded onto Heparin HiTrap HP 5 ml columns (Cytiva) using an ÄKTAPurifier system. The column was washed with 0% and 10% of wash buffer and eluted with 30% of elution buffer (RecA: 20 mM Tris–HCl, pH 8.0, 1.5 M NaCl, 2 mM TCEP), and all fractions were collected. Fractions were dialyzed overnight at 4 °C into RecA

storage buffer (20 mM Tris–HCl, pH 8.0, 200 mM NaCl, 2 mM TCEP, 10% glycerol) and stored at -80 °C.

Overexpression of LexA mutant: In a 1.7 mL eppendorf tube 50 μL aliquot of electrocompetent BL21(DE3) cells were transformed with three plasmids 1–1.5 μL (~50–200 ng) each, one containing the recombinant LexA gene bearing the amber stop codon and ochre stop codon at the desired position for Acd and Tet incorporation respectively, and the other two encoding the aminoacyl tRNA synthetase with improved specificity for Acd and Tet incorporation. The mixture was then transferred into pre-chilled 1 mM electro-cuvettes. After electroporation according to the instrument settings, the cells were immediately resuspended in 1 mL SOC media, transferred to an Eppendorf tube, and incubated at 37 °C with shaking at 250 rpm for 90 minutes. Cells transformed using electroporation were grown on LB/agar plates at 37 °C overnight with 25 μg/mL chloramphenicol, 100 μg/mL streptomycin, and 50 μg/mL kanamycin as required antibiotics. For starter culture, an individual colony was seeded into 5 mL of non-inducing media (NIM) with selective antibiotics and grown at 37°C overnight with shaking at ~250 rpm. To scale up expressions, ~1% inoculum (2 mL) of NIM culture was added to 200 mL of auto-induction media in 1 L baffled flasks with selective antibiotics and grown at 37 °C. After ~1-2 hours, solubilized Acd and Tet were added to a final concentration of 0.5 mM, and continued growth at 37 °C until OD_{600} was ~1.5, and then the temperature was reduced to 30 °C. After growth for 24 hours, cells were harvested and stored at -20 °C.

Cell lysis and protein purification: Cells were resuspended in lysis buffer containing 20 mM sodium phosphate pH 6.8, 500 mM NaCl, 25 mM imidazole, 250 U/mL benzonase nuclease (Sigma) and 0.25 mg/mL lysozyme (Sigma); cells were then lysed using a sonicator. Lysates were then centrifuged (Sorvall SS34 rotor) for 15 mins at 26900 RCF. Clarified lysates were affinity purified with HisPur cobalt resin (Thermo Fisher) using a 25 to 400 mM imidazole gradient. To further purify the HisPur eluted protein, size exclusion chromatography was done using Superdex™ 200 pg in 50 mM Tris-HCl pH 7.0, 200 mM NaCl, 0.5 mM EDTA, and 10% glycerol. Fractions containing purified Acd/Tet-labeled LexA were stored at -80 °C.

Overexpression of WT-RecA: The RecA construct was overexpressed in BL21(DE3) cells containing the recombinant RecA gene. Overnight cultures were grown in LB media with kanamycin at 37 °C with shaking at ~250 rpm and diluted 1:100 into fresh 1L LB media. Cultures were grown to an optical density at 600 nm of 1.2 before induction with 1 mM IPTG. After 5 hours at 37 °C, harvested cells were resuspended in RecA lysis buffer with cOmplete EDTA-free protease inhibitor cocktail tablet (Sigma). Cell lysis and purification were similar to those of previously described RecA mutants.

Overexpression of WT-LexA: The LexA construct was overexpressed in BL21(DE3) cells containing the recombinant LexA gene. Overnight cultures were grown in LB media with kanamycin at 37 °C with shaking at ~250 rpm and diluted 1:100 into fresh 1L LB media. Cultures were grown to an optical density at 600 nm of 0.6-0.8 before induction with 1 mM IPTG. After 20 hours at 37 °C, harvested cells were resuspended in LexA lysis buffer with cOmplete EDTA-free protease inhibitor cocktail tablet (Sigma).

Cell lysis and purification were similar to those of previously described LexA mutants.

Purification and Characterization of CaM$\tau_{93}\delta_{113}$

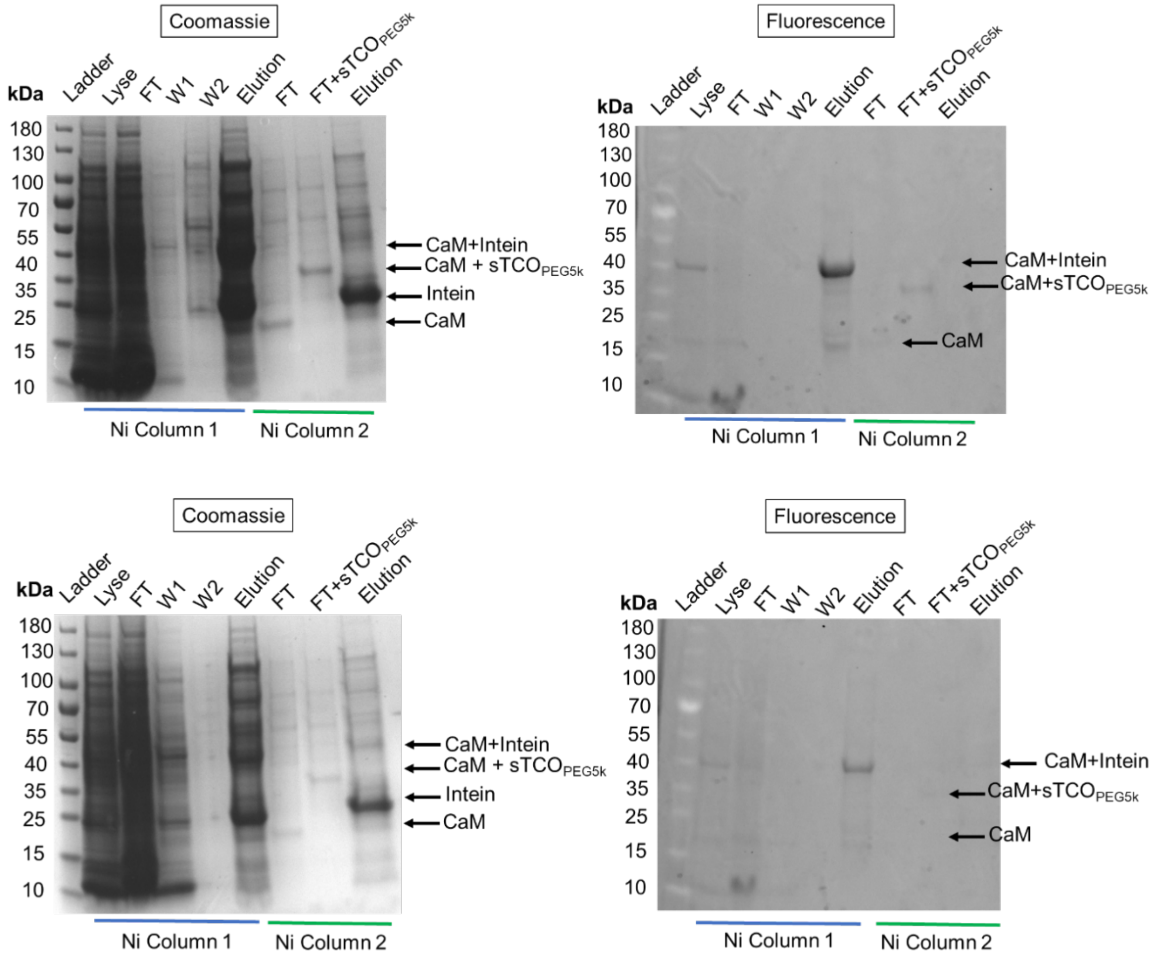

Figure S9. Top: SDS-PAGE analysis of expression of CaM$\tau_{93}\delta_{113}$ at 25 ˚C (top) and 30 ˚C (bottom). Left: gel code blue stain and Right: fluorescence. MW = molecular weight in kDa. Lyse = lysate, FT = flow through, W1 = wash 1, and W2 = wash 2. Excitation Wavelength of Acd is 350 nm for fluorescence gel imaging.

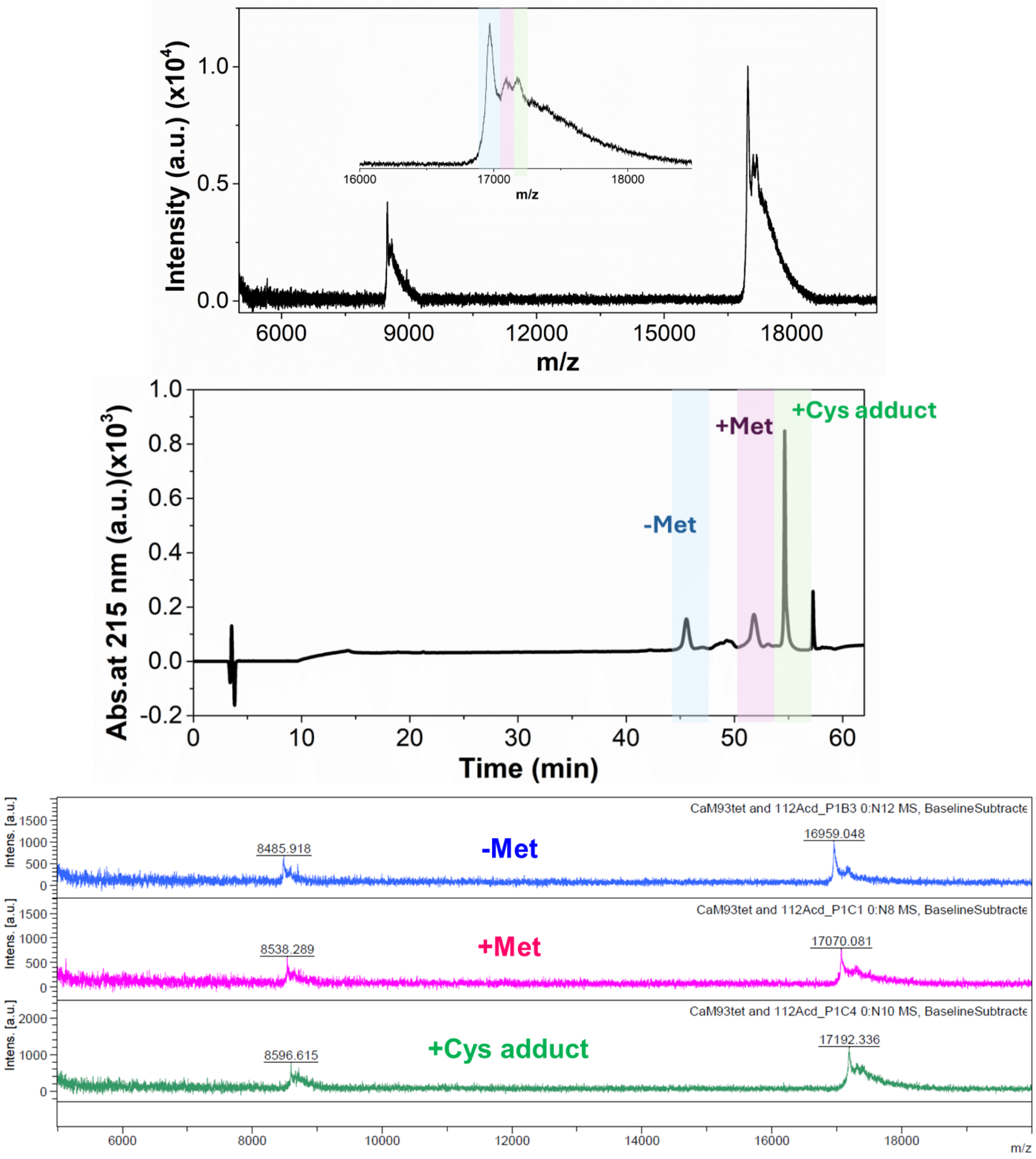

Figure S10. Top: MALDI-TOF-MS characterization of FPLC purified CaM$\tau_{93}\delta_{113}$. Middle: Analytical HPLC chromatogram showing the separation profile of CaM$\tau_{93}\delta_{113}$ from Cys adduct of Tet. *Bottom:* MALDI-MS analysis of the individual fractions collected from the analytical HPLC, confirming the purity of full-length CaM$\tau_{93}\delta_{113}$, as well as the presence of its variant, the N-terminal methionine-cleaved form and the cysteine adduct with Tet.

Purification and Characterization of RecA$\tau_{33}\delta_{102}$

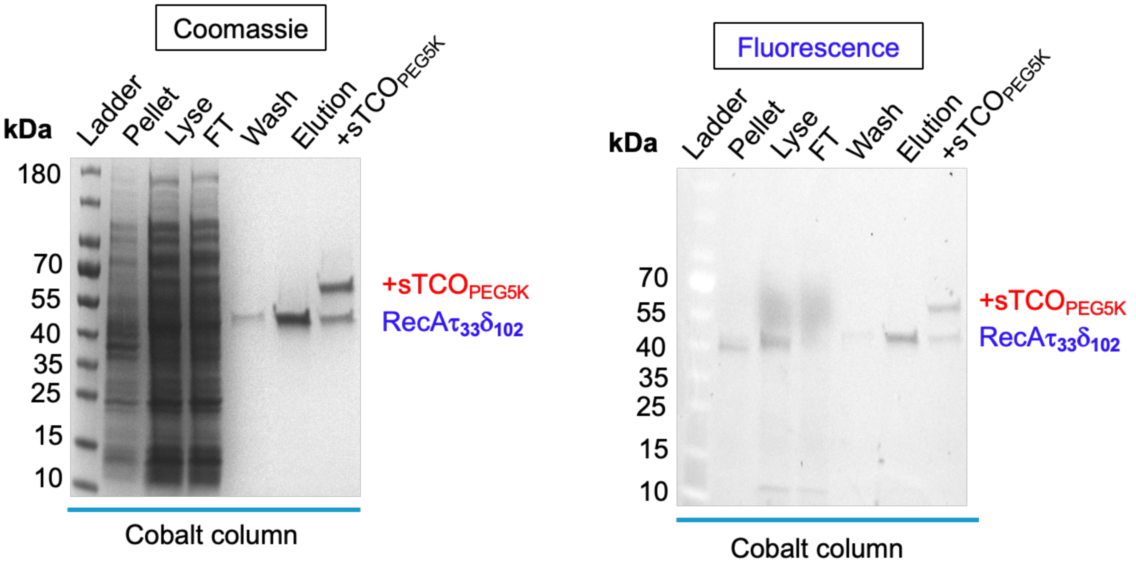

Figure S11. SDS-PAGE analysis of expression of RecA$\tau_{33}\delta_{102}$ at 30 ˚C. Left: gel code blue stain, and Right: Fluorescence. MW = molecular weight in kDa. Lyse = lysate, FT = flow through. Excitation wavelength of Acd is 350 nm for fluorescence gel imaging.

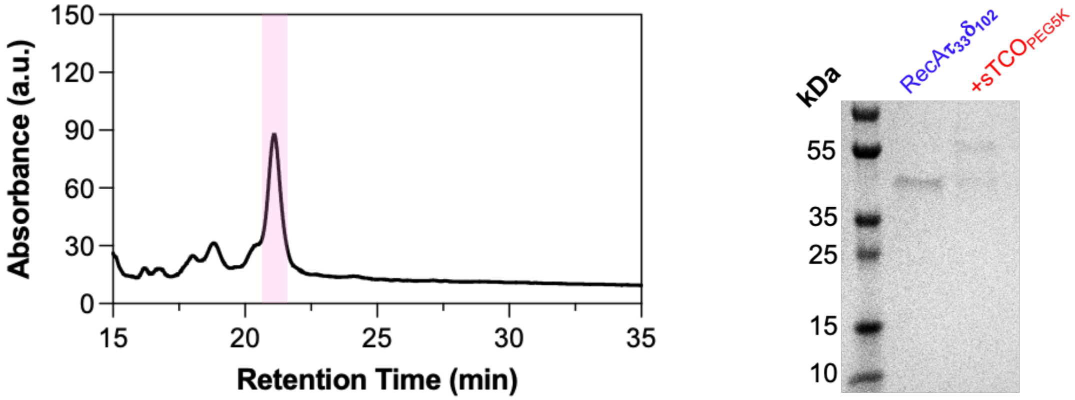

Figure S12. Left: Analytical HPLC chromatogram showing the purity of RecA$\tau_{33}\delta_{102}$. Peak absorption measured at 215 nm. Right: Gel analysis of RecA$\tau_{33}\delta_{102}$ after analytical HPLC and confirmation of Tet incorporation by reaction with sTCO$_{PEG5k}$.

Purification and Characterization of LexA$\tau_{81}\delta_{86}$

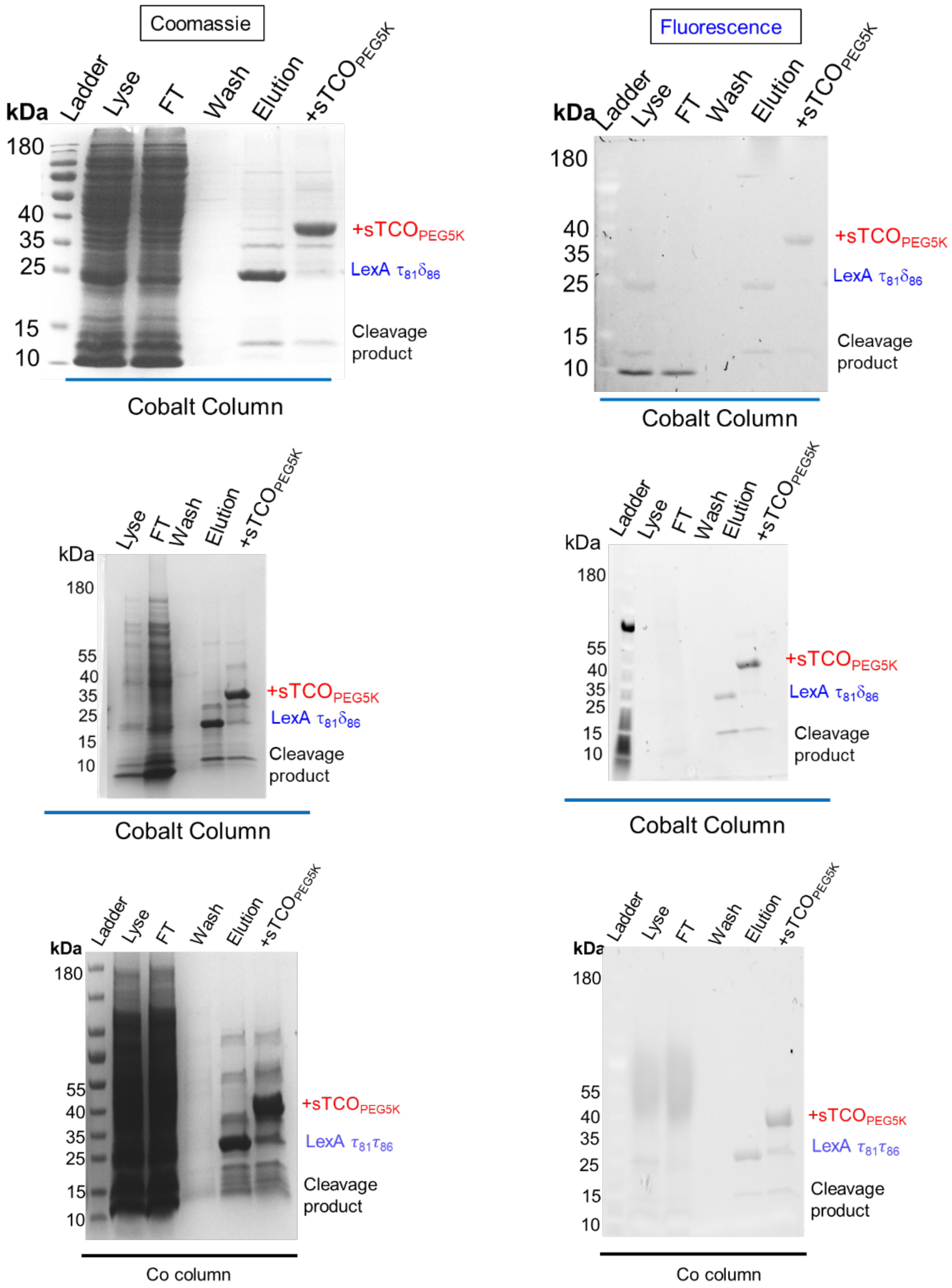

Figure S13. SDS-PAGE analysis of expression of LexA$\tau_{81}\delta_{86}$ at 30 ˚C in three independent protein expressions. Left: gel code blue stain, and Right: Fluorescence. MW = molecular weight in kDa. Lyse = lysate, FT = flow through. Excitation wavelength of Acd is 350 nm for fluorescence gel imaging.

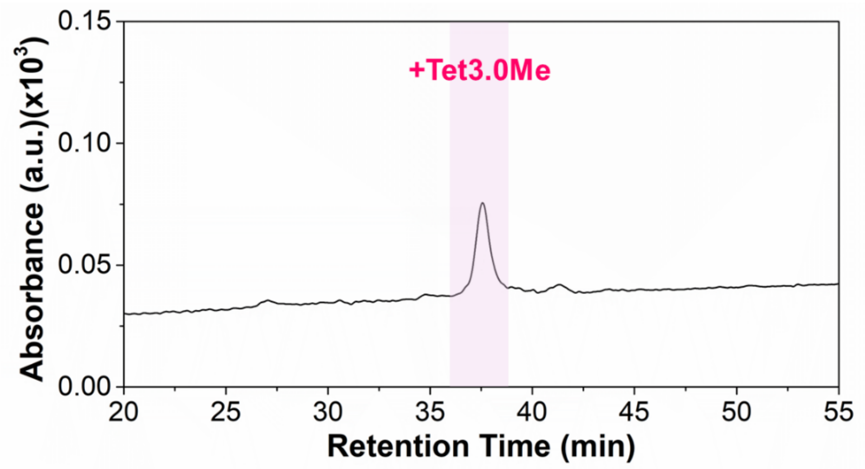

Figure S14. Analytical HPLC chromatogram showing the purity of LexA$\tau_{81}\delta_{86}$. Peak absorption measured at 215 nm.

Purification and Characterization of LexA$\delta_{81}\tau_{86}$

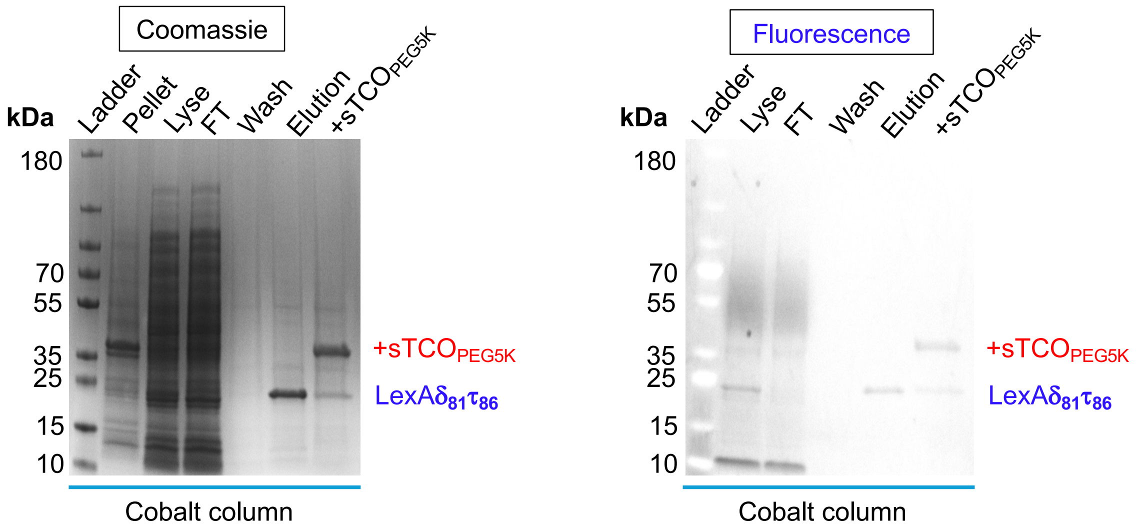

Figure S15. SDS-PAGE analysis of expression of LexA$\delta_{81}\tau_{86}$ at 30 ˚C. Left: gel code blue stain, and Right: Fluorescence. MW = molecular weight in kDa. Lyse = lysate, FT = flow through. Excitation wavelength of Acd is 350 nm for fluorescence gel imaging.

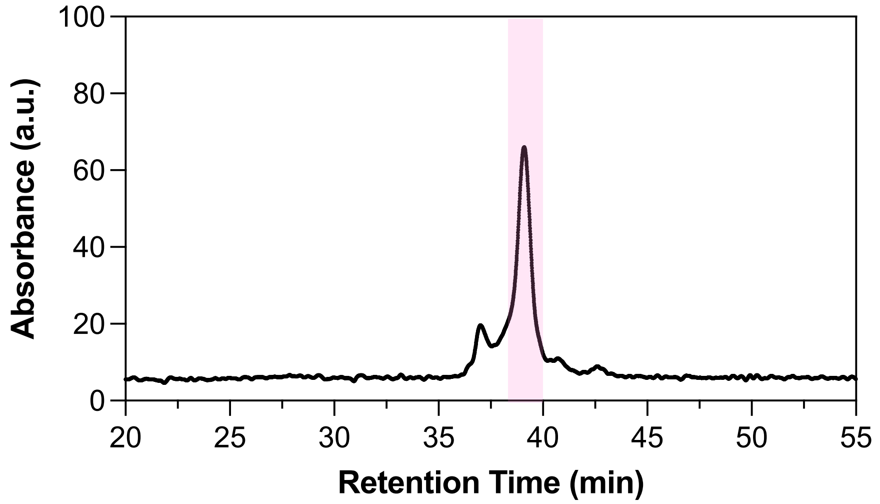

Figure S16. Analytical HPLC chromatogram showing the purity of LexA$\delta_{81}\tau_{86}$. Peak absorption measured at 215 nm.

Purification and Characterization of LexA$\tau_{86}\delta_{175}$ (K156A)

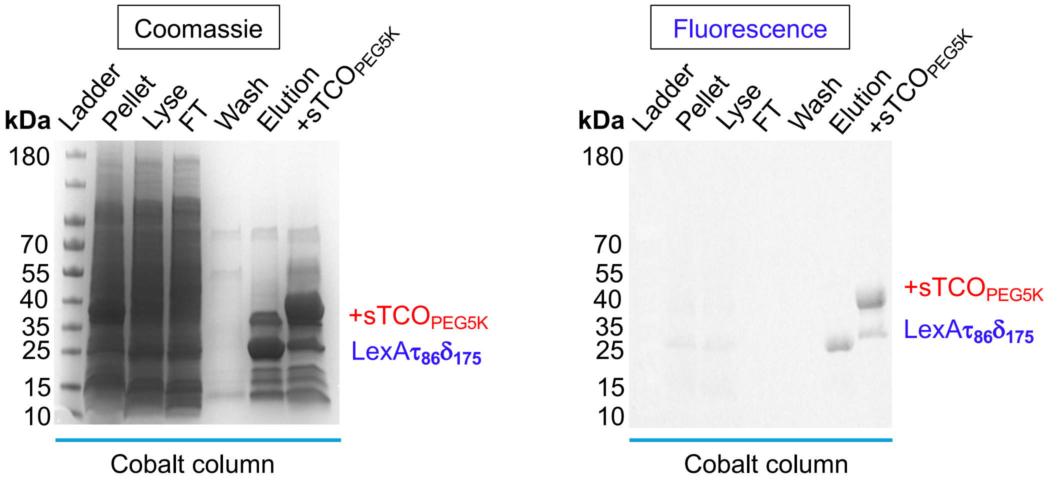

Figure S17. SDS-PAGE analysis of expression of LexA$\tau_{86}\delta_{175}$ (K156A) at 30 ˚C. Left: gel code blue stain, and Right: Fluorescence. MW = molecular weight in kDa. Lyse = lysate, FT = flow through. Excitation wavelength of Acd is 350 nm for fluorescence gel imaging.

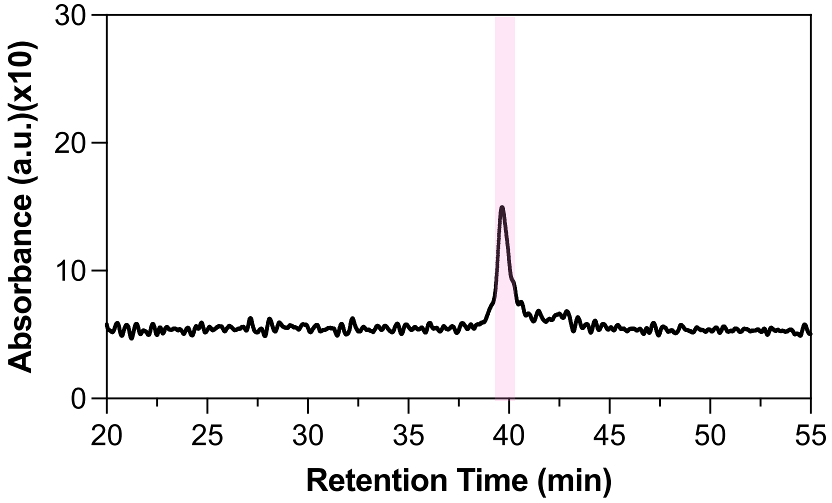

Figure S18. Analytical HPLC chromatogram showing the purity of LexA$\tau_{86}\delta_{175}$ (K156A). Peak absorption measured at 215 nm.

Purification and Characterization of LexA$\tau_{86}\delta_{175}$ (QM-L_{89}P, Q_{92}W, E_{152}A, and K_{156}A)

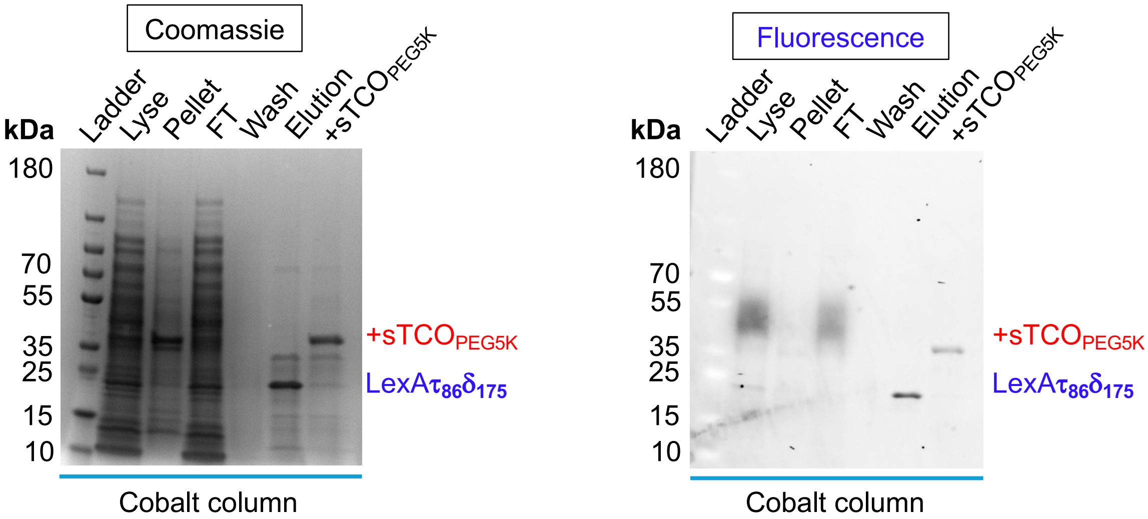

Figure S19. SDS-PAGE analysis of expression of LexA$\tau_{86}\delta_{175}$ (QM) at 30 ˚C. Left: gel code blue stain, and Right: Fluorescence. MW = molecular weight in kDa. Lyse = lysate, FT = flow through. Excitation wavelength of Acd is 350 nm for fluorescence gel imaging.

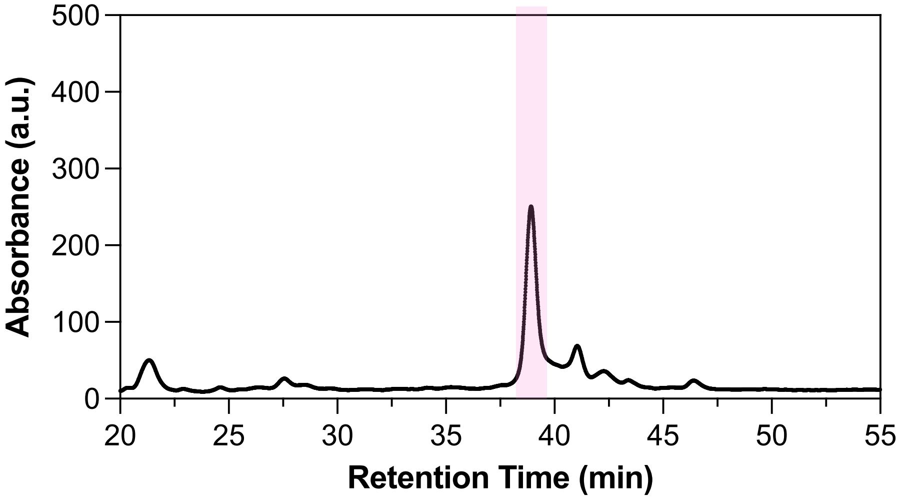

Figure S20. Analytical HPLC chromatogram showing the purity of LexA$\tau_{86}\delta_{175}$ (QM). Peak absorption measured at 215 nm.

Purification of LexAδ175 (K156A) and LexAδ175(QM)

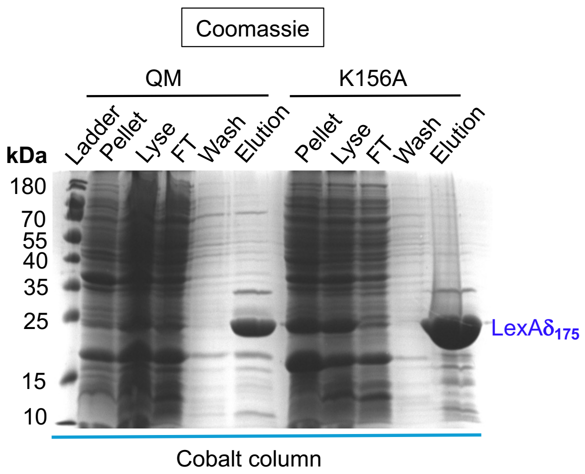

Figure S21. SDS-PAGE analysis of expression of LexAδ175 (QM) and LexAδ175 (K156A) at 30 ˚C. Left: gel code blue stain, and Right: Fluorescence. MW = molecular weight in kDa. Lyse = lysate, FT = flow through. Excitation wavelength of Acd is 350 nm for fluorescence gel imaging.

Oxidation of DihydroTet to Tet by Horseradish Peroxidase (HRP). We prepared a 40 μM stock solution of HRP (Pierce™ Horseradish Peroxidase, Fisher Scientific, USA) in 100 mM phosphate buffer (pH 6.5) and stored it at -80 °C until further use. Separately, we prepared a 25 μM solution of $LexA_{\tau 81 \delta 86}$ in 100 mM phosphate buffer (pH 7.4), ensuring compatibility with HRP activity at pH 7.4. To these solutions, we added varying concentrations of HRP (0, 15 nM, and 1 μM) and incubated them at 37 °C with shaking at 250 rpm using an IKA MS3 control orbital shaker (Wilmington, NC, USA) for 20 minutes. After the incubation, Tet was reacted with either 1 equivalent or 2 equivalents of $sTCO_{PEG5K}$ (10 mM stock solution in H_2O). The reaction mixture was further incubated at 37 °C with shaking at 250 rpm for 5 minutes.
After 5 minutes of incubation, 5 μL of loading dye was added to 15 μL of each sample. The samples were then boiled for 5 minutes to denature the proteins. The denatured samples were loaded onto a 4% stacking and 12% resolving SDS-PAGE gel and run at 100–140 volts for 1 hour until the dye front reached the bottom of the gel. The gel was fixed in a destaining solution (40% (v/v) methanol and 10% (v/v) glacial acetic acid in Milli-Q water), followed by staining with GelCode Blue. The gel was subsequently destained with H_2O to remove excess dye and imaged using a G:Box mini (Syngene).

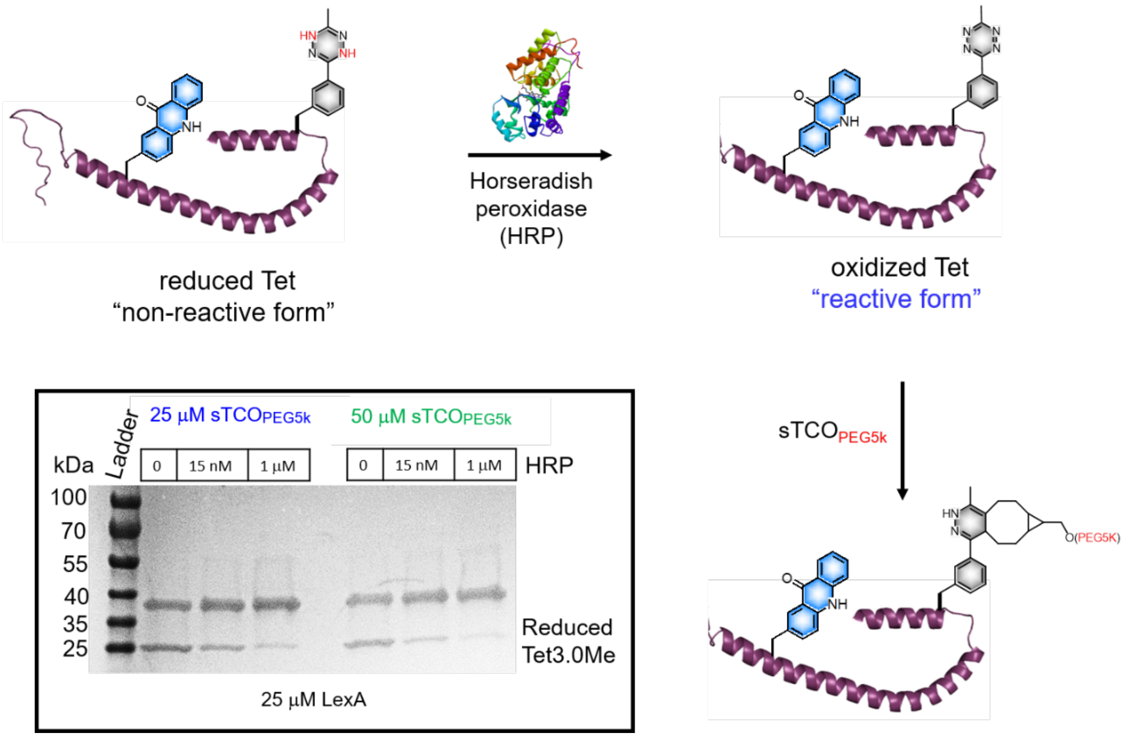

Figure S22. Schematic representation of Tet oxidation of $LexA_{\tau 81 \delta 86}$ (25 μM) by HRP and followed by gel shifting assay by labeling with the $sTCO_{PEG5K}$. Inset: SDS-PAGE of $LexA_{\tau 81 \delta 86}$ construct treated with varying concentrations of HRP (0, 15 nM, and 1 μM), followed by labeling with either 1 equiv. or 2 equiv. of $sTCO_{PEG5K}$.

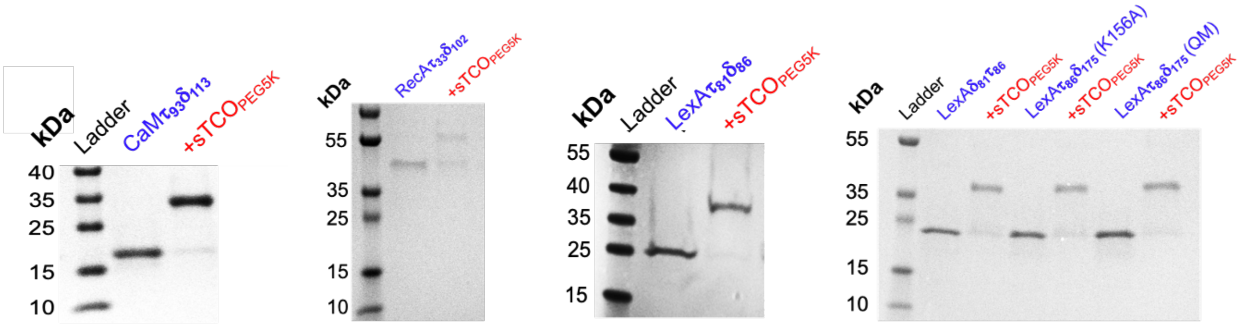

Figure S23. SDS-PAGE analysis of Tet IEDDA reaction of CaM, RecA, and LexA dual constructs with $sTCO_{PEG5k}$.

Table S7. Single and Dual Incorporation of Tet and Acd in Proteins Expressed in *E. coli*.

Protein of Interest (POI)	Tet at TAA codon	Acd at TAG codon	Total Yield[a] (mg)/100 mL	Calculated Mass (m/z)	Observed Mass (m/z)
CaM	93	113	3.5 (3.3)[b]	16950.8	16959.0
RecA	33	102	2.0	39341.7	39336.5
LexA	81	86	2.4	23803.8	23804.1
LexA	86	81	2.5	23839.2	23837.6
LexA (K156A)	86	175	2.3	23810.1	23803.6
LexA (QM)	86	175	2.1	23794.1	23785.4
LexA (K156A)	-	175	2.8	23679.9	23670.7
LexA (QM)	-	175	3.0	23663.9	23654.3

[a]Total protein yield was determined using the DC assay. [b]Yield shown in parentheses corresponds to the pure protein; the difference from the total yield is attributed to cysteine adduct formation.

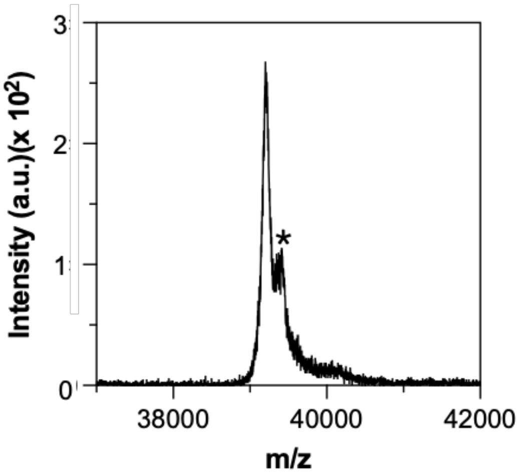

Figure S24. MALDI-TOF MS analysis of Tet and Acd incorporation in RecA. The data show masses corresponding to the successful incorporation of Tet and Acd. An asterisk (*) denotes a known matrix adduct.

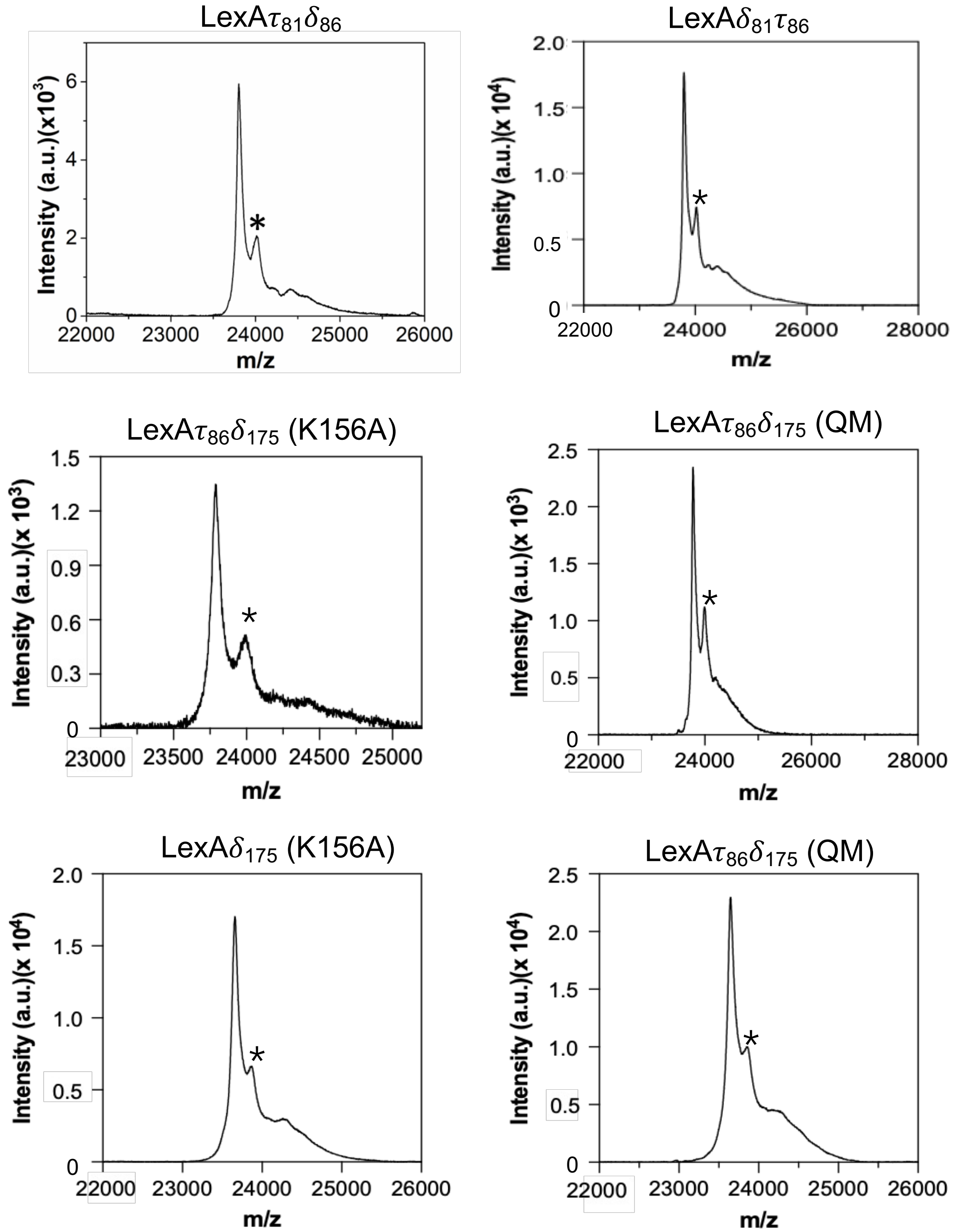

Figure S25. MALDI-TOF MS analysis of Tet and Acd incorporation in LexA. The data show masses corresponding to the successful incorporation of Tet and Acd. An asterisk (*) denotes a known matrix adduct.

Conformational Changes in CaM Upon Ca^{2+} Sensing and Tet Photolysis

Fluorescence spectral measurements of CaM$\tau_{93}\delta_{113}$ with Ca^{2+} ions. The dual construct CaM$\tau_{93}\delta_{113}$ (25 µM) in 20 mM Tris buffer (pH 8.0) was titrated with varying concentrations of Ca^{2+} (0, 0.5, 2.5, 7.5, 15, and 25 µM). The mixtures were mixed homogeneously by pipetting in Greiner 96-well flat black half-area plates at room temperature. Fluorescence spectra were recorded using a Tecan Spark plate reader (Männedorf, Switzerland) with an excitation wavelength (λ_{ex}) of 385 nm using the following parameters: excitation and emission bandwidths of 5 nm, delay time of 0 µs, and integration time of 40 µs.

Similarly, we measured fluorescence intensity to validate the conformational change. The dual construct CaM$\tau_{93}\delta_{113}$ (25 µM) in 20 mM Tris buffer (pH 8.0) was titrated by altering the order of addition of Ca^{2+} (25 µM) and sTCO-OH (50 µM) via an IEDDA reaction. Fluorescence intensity was measured using a Tecan Spark plate reader (Männedorf, Switzerland) with an excitation wavelength (λ_{ex}) of 385 nm and an emission wavelength of 450 nm. The following parameters were used: excitation and emission bandwidths of 5 nm, delay time of 0 µs, and integration time of 40 µs.

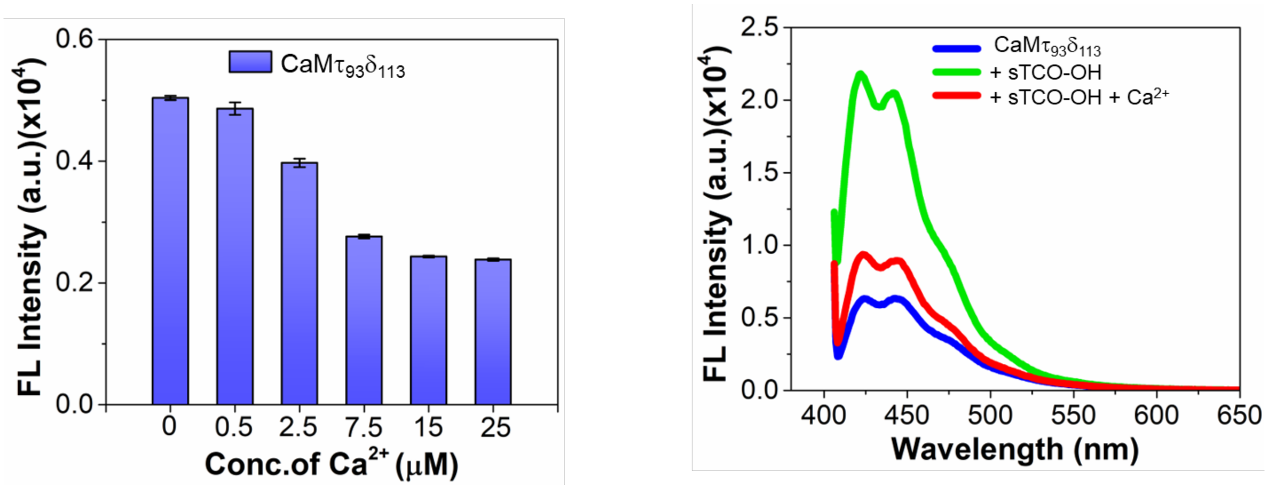

Figure S26. Left: Fluorescence spectral changes of CaM$\tau_{93}\delta_{113}$ upon titration with Ca^{2+} ions in 20 mM Tris at pH 8.0, demonstrating the fluorescence response to calcium-induced conformational changes. Right: Validation of conformational changes through the retention of Acd fluorescence, analyzed by the addition of sTCO-OH (*via* the IEDDA reaction) and subsequent addition of Ca^{2+} ions (25 µM) in 20 mM Tris at pH 8.0. Excitation and emission wavelengths for Acd were 385 nm and 450 nm, respectively. Error bars represent the SD of 3 measurements.

Retention of Acd fluorescence by photolysis of Tet under 254 nm light irradiation. The fluorescence intensity of the dual construct CaM$\tau_{93}\delta_{113}$ (25 µM) in Greiner 96-well flat black half-area plate was measured in the presence or absence of Ca^{2+} in 20 mM Tris buffer (pH 8.0) at 0 minutes. The plate was then irradiated with a 254 nm light source (USHIO G8T5 low-pressure mercury-arc lamp, 7.2 watts) or 365 nm light source (ANALYTIK JENA, Mfr. No.34-0006-01, 8 watts) placed 5 cm away. Fluorescence intensity was recorded at different intervals of irradiation time.

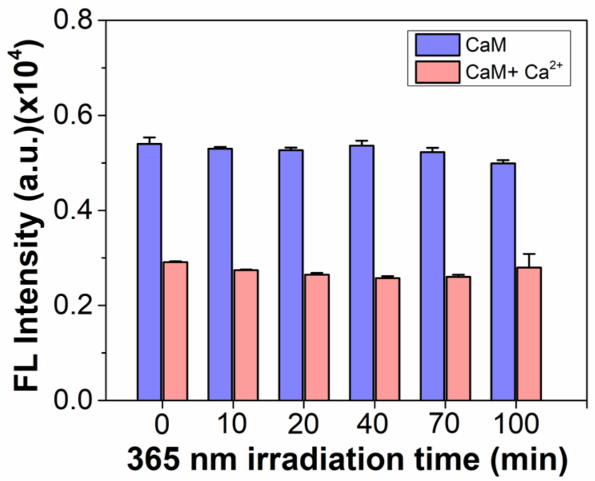

Figure S27. Fluorescence intensity changes during the photolysis of CaM$\tau_{93}\delta_{113}$ (25 µM) with and without Ca^{2+} ions (25 µM) upon 365 nm light irradiation. Ex/Em wavelength of Acd is 385 nm/450 nm.

LexA Cleavage

Alkaline-mediated and RecA*-mediated LexA auto-proteolysis assays. LexA cleavage assays were performed essentially as previously described.[17] For the alkaline-mediated auto-proteolysis assay, 25 μM of His-LexA$\tau_{81}\delta_{86}$ was either mixed with an equal volume of RecA control buffer (70 mM Tris-HCl pH 7.5 or 8.5, 10 mM $MgCl_2$, 2 mM TCEP, 150 mM NaCl, 50 μg/mL BSA) or with an equal volume of alkaline pH reaction buffer (100 mM Tris-glycine-CAPS pH 10.6, 300 mM NaCl). Fluorescence intensity of Acd was monitored at 37 °C using Tecan Spark microplate reader with excitation wavelength at 385 nm and emission wavelength at 450 nm for 12 hours.

For the RecA*-mediated auto-proteolysis assay, RecA was activated by mixing His-RecA and 18-mer poly-GGT ssDNA (5'-GGT GGT GGT GGT GGT GGT-3' from Integrated DNA Technologies, Coralville, Iowa, USA.) in RecA activation buffer (70 mM Tris-HCl, pH 7.5, 10 mM MgCl2, 2 mM TCEP, 150 mM NaCl, 50 μg/mL BSA, and 0.25 mM ATPγS). The reactions were incubated for 3 hours at 25 °C. Activated RecA* was then added 1:1 with 25 μM of His-LexA$\tau_{81}\delta_{86}$, and the fluorescence intensity of Acd was monitored at 25 °C using Tecan Spark microplate reader. A non-activated RecA control was prepared similarly, with the exclusion of ATP-S.

Simultaneously, a gel-based assay was also used to examine His- LexA$\tau_{81}\delta_{86}$ auto-proteolysis in the presence of activated RecA (RecA*) or alkaline pH. At time zero, 5 μL RecA* and LexA were independently added to 10 μL of a stop mixture containing 2x Laemmli buffer (BioRad). For the other time points, 10 μL reaction mixture was added to 10 μL of 2x Laemmli buffer and boiled at 95 °C for 5 minutes. 20 μL of each sample was loaded onto a 15% SDS-PAGE gel, run at 150 V for 60 minutes, and stained with GelCode™ Blue Stain Reagent (Thermo Fisher) to visualize cleavage bands. Following gel image acquisition, protein bands were quantified using ImageJ (NIH). The percentage of cleaved LexA was calculated as the ratio of the C-terminal fragment band over the sum of the full-length and C-terminal fragment bands.

Gel Analysis. Samples were collected during cell lysis, nickel column purification, intein cleavage, and the second nickel column purification to confirm expression and monitor protein loss. For SDS-PAGE, 5 μL of loading dye was added to 5 μL of each sample, diluted to 20 μL with water, and boiled for 5 minutes to denature the proteins. The samples were run on a 4% stacking and 12% resolving gel at 60-140 volts for 1.5 hours until the dye front reached the gel bottom. The gel was fixed in a destaining solution (40% (v/v) methanol, 10% (v/v) glacial acetic acid in Milli-Q water) to remove non-covalently bound Acd or Tet. Fluorescent imaging for Acd-labeled proteins was performed using a G:Box mini (Syngene) before overnight staining with Gel Code Blue and subsequent destaining to remove excess dye. Representative gels for each protein construct are shown below.

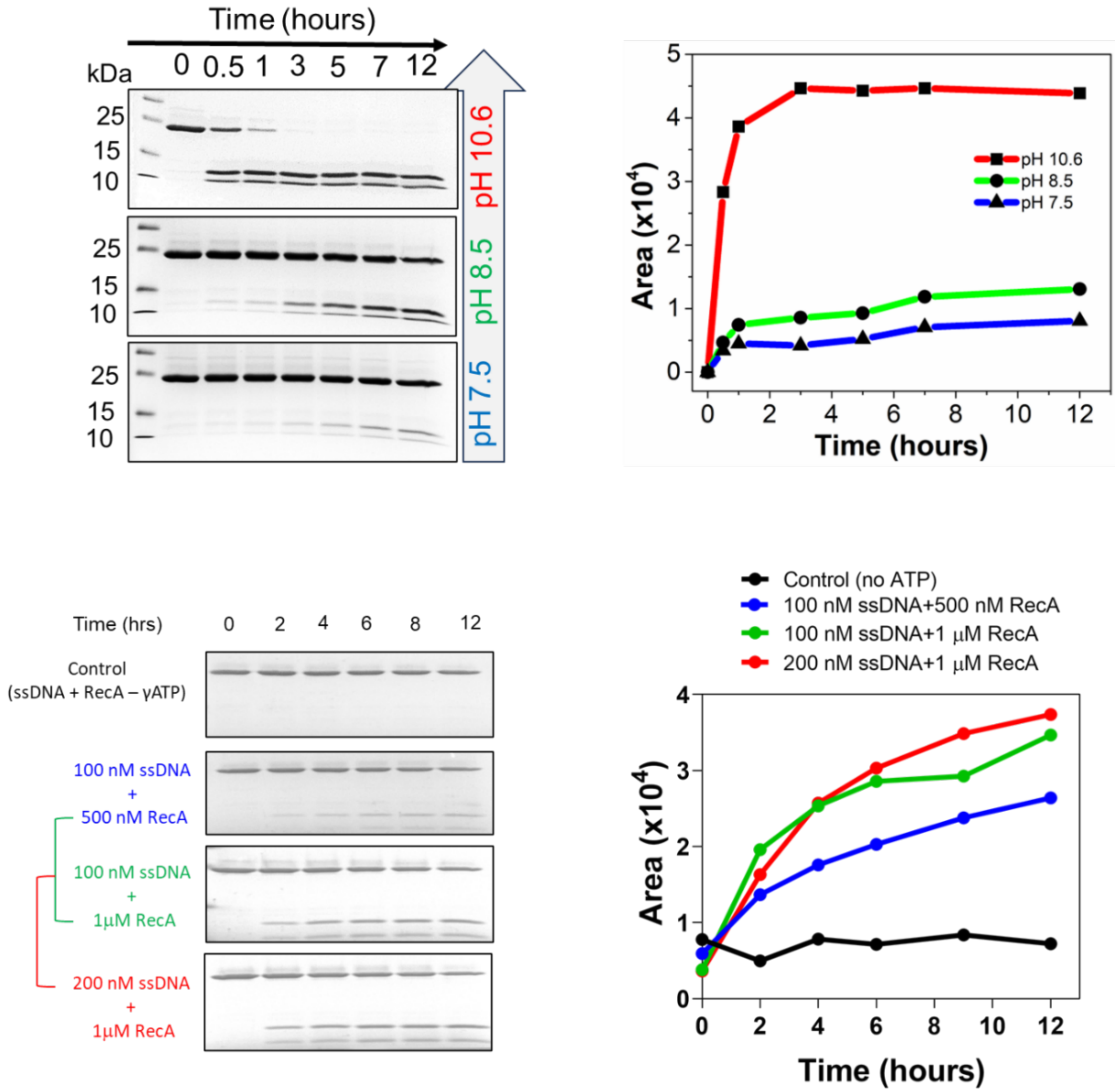

Figure S28. Top left: Quantitative validation of LexA (LexA$\tau_{81}\delta_{86}$) cleavage by SDS-PAGE gel analysis under varying pH conditions. Top right: pH-mediated LexA cleavage kinetics analyzed using ImageJ based on the gel bands of SDS-PAGE, correlating with fluorescence-based measurements. Bottom left: Quantitative validation of RecA* (with varying ssDNA or RecA concentrations) induced LexA cleavage by SDS-PAGE gel analysis. Bottom right: RecA*-mediated LexA (LexA$\tau_{81}\delta_{86}$) cleavage kinetics analyzed using ImageJ based on the gel bands of SDS-PAGE, correlating with fluorescence-based measurements.

RecA activation Reactions
A 2x concentrated reaction was prepared in activation buffer containing 70 mM Tris-HCl, pH 7.5, 10 mM MgCl2, 2 mM TCEP, 150 mM NaCl, 50 μg/mL BSA, and 0.25 mM ATPγS with His-RecA and 18-mer poly-GGT ssDNA (5'-GGT GGT GGT GGT GGT GGT-3'. The reactions were incubated for 3 hours at 25 °C. For experiments involving concentration series of RecA*, the ssDNA substrate was maintained at a 4:1 nucleotide to RecA monomers. Non-activated RecA control was prepared similarly, with the exclusion of ATPγS.

Fluorescence Anisotropy Measurements
All stopped-flow anisotropy measurements were made in a KinTek AutoSF-120 stopped-flow spectrofluorometer, with the following settings. A Xenon/Mercury lamp (Hamamatsu) with a monochromator (Oriel) was set to emit light through a fiber optic at 385 nm with a 3.16 mm diffraction grating. Incident light was plane-polarized using a polarized optical fiber and passed into the observation chamber. Emitted light was filtered with two 440 +/− 40 nm emission filters (Edmund Optics) with polarized films in the vertical or horizontal direction for either photomultiplier tube (PMT). PMT voltages were calibrated, and a grating factor (G-factor) was obtained at the start of each instrument run.
Mixtures of LexA at 0.5 μM and RecA* at different concentrations (0.08, 0.32, 1.28, 5.12, and 15 μM) were monitored for changes in fluorescence anisotropy for 60 seconds. The means at all times for each curve were imported into KinTek Explorer global fitting software. Sigma calculation and normalization for each trace were performed using the built-in afit (analytical fit) and concentration scaling factor analysis, respectively, present in the software. Parameters in a one-step reversible binding model with LexA and RecA in a 1:1 stoichiometry were manually adjusted. The imported data were initially fitted by visual inspection, followed by refinement using the software's built-in regression tools. In brief, the model parameters were adjusted to minimize the Chi² value, defined as the sum of squared residuals normalized by the standard error (σ) at each time point.

$$\chi^2 = \sum_{i=0}^{N-1} \left(\frac{y_i - y(x_i)}{\sigma_i} \right)^2$$

Subsequently, the optimized parameters were systematically varied to identify ranges that still produced acceptable fits, based on Chi² analysis. This approach helps assess how well the data constrain the model. A Chi² threshold of 0.70 was chosen as a moderately strict cutoff to define upper and lower bounds for each parameter. At this threshold, most experimental data points fall within the predicted confidence bounds of the fitted curves upon visual inspection.

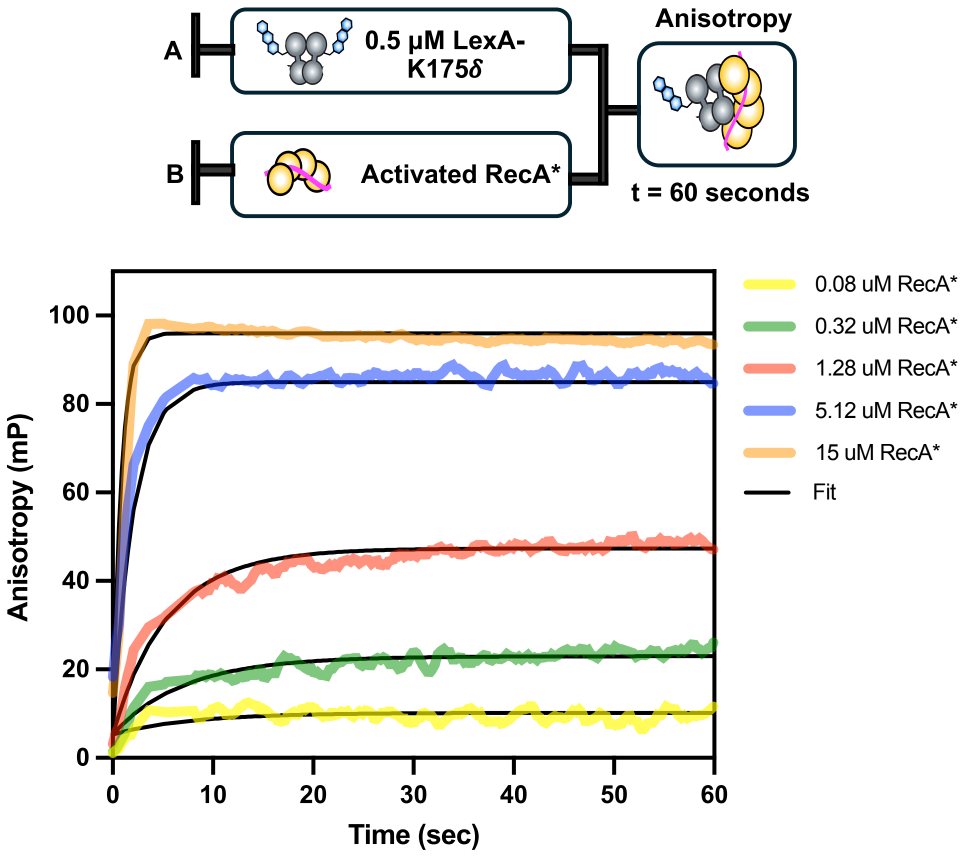

$$\mathrm{LexA + RecA^*} \underset{\mathbf{0.079\ s^{-1}}}{\overset{\mathbf{0.084\ \mu M^{-1} s^{-1}}}{\rightleftharpoons}} \mathrm{LexA^*\!: RecA^*}$$

$$\mathbf{K_D = 0.94\ \mu M}$$

Figure S29. Association kinetics of LexAδ$_{175}$ (K156A) with RecA. 500 nM of LexAδ$_{175}$ (dimer) was rapidly mixed with a 1:4 ratio of RecA* and anisotropy was measured for 60 seconds. The best-fit curves from reaction simulations after globally fitting three independent concentration series experiments are shown as solid black lines for each concentration.

$$\mathrm{LexA + RecA^*} \underset{\mathbf{k_{-1}}}{\overset{\mathbf{k_1}}{\rightleftharpoons}} \mathrm{LexA^*\!: RecA^*}$$

$$\mathrm{FP} = \frac{\mathbf{a} \times [\mathrm{LexA}] + \mathbf{b} \times [\mathrm{LexA^*\!: RecA^*}]}{[\mathrm{LexA}] + [\mathrm{LexA^*\!: RecA^*}]}$$

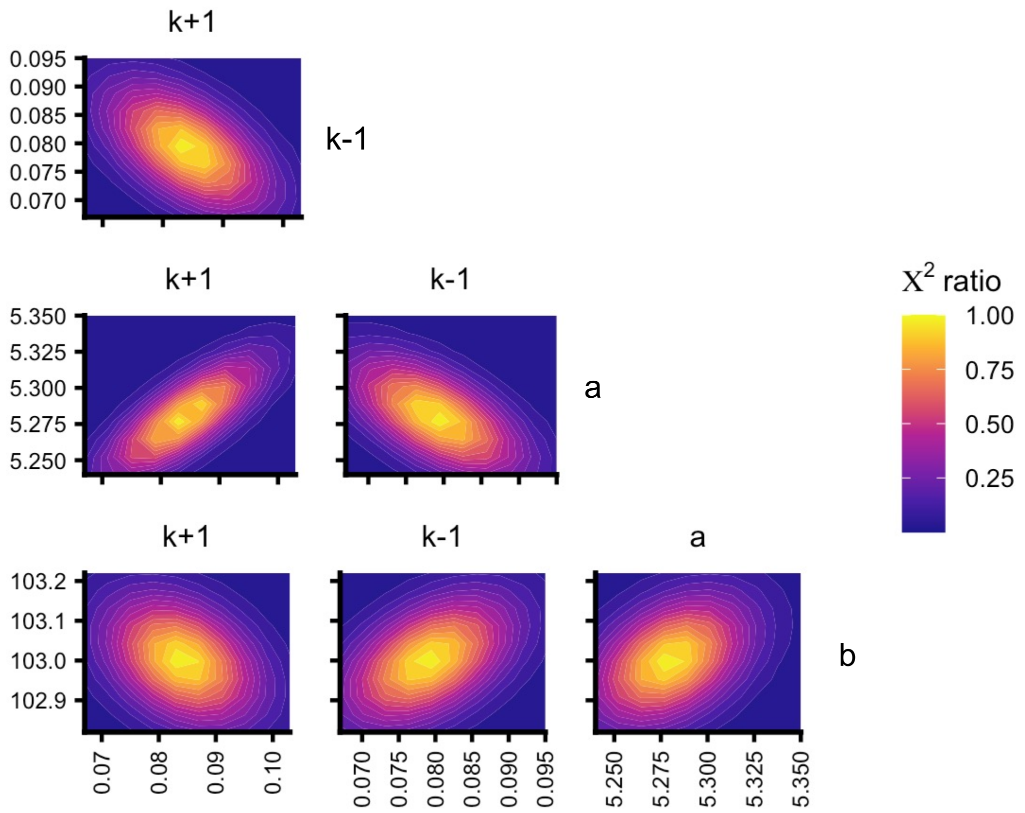

Figure S30. One-step reversible binding model for LexA:RecA* interaction. (Top) Fitted parameters include the forward and reverse rate constants, k_1 and k_{-1}, along with two factors, a and b, used to convert the protein concentrations to simulated fluorescence anisotropy signals. (Bottom) Two-dimensional FitSpace confidence intervals for these fitted parameters, with higher Chi^2 ratios corresponding to better fits.

Table S8. Fitted values for reversible, one-step binding model parameters

Parameter	Best fit value	Lower, Upper boundaries (Chi^2 threshold of 0.70)
k_1	0.0842	0.079, 0.087
k_{-1}	0.079	0.0763, 0.0825
a	5.28	5.26, 5.29
b	103	102.9, 103.1
C_1	1	N/A
C_2	0.97	0.96, 0.98
C_3	0.95	0.94, 0.96

Table S9. Summary of binding model fit results

Model characteristic	Result
Data points	10105
Degrees of Freedom (DoF)	10098
Sigma w.r.t. fit	0.319
Chi2	14644
Chi2/DoF	1.45
p-value	0
Chi2 Threshold	0.998116

Fluorescence Anisotropy Measurements for LexA$\tau_{86}\delta_{175}$ (QM):
Mixtures of LexA at 0.5 μM and RecA* at different concentrations (0, 0.08, 0.32, 1.28, 5.12, and 15 μM) were monitored for changes in fluorescence anisotropy for 60 seconds. Used the previous one-step reversible binding model for fitting the experimental data. Fits were optimized using built-in regression analysis in the software, similar to the previously mentioned fluorescence anisotropy data fitting.

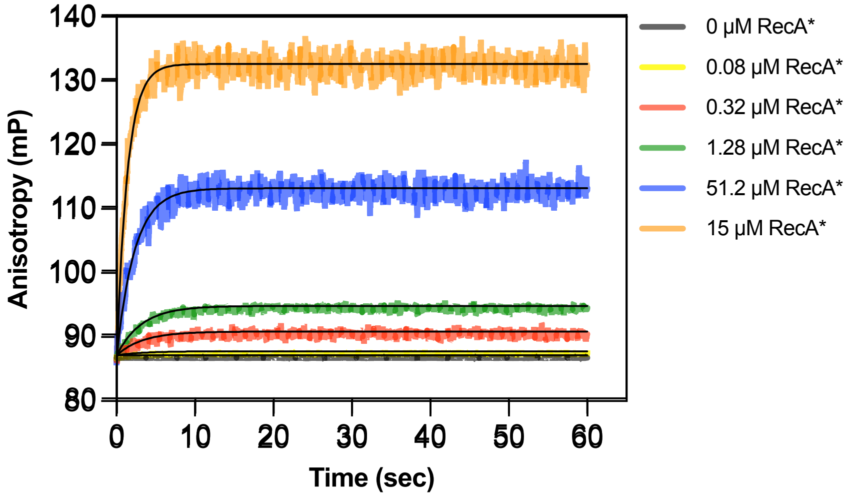

$$\text{LexA} + \text{RecA}^* \underset{0.269\ \mathrm{s^{-1}}}{\overset{0.030\ \mu\mathrm{M^{-1}s^{-1}}}{\rightleftharpoons}} \text{LexA}^*\text{: RecA}^*$$

K_D = 8.9 μM

Figure S31. Association kinetics of LexAδ_{175} (QM) with RecA. 500 nM of LexAδ_{175} (dimer) was rapidly mixed with a 1:4 ratio of RecA*, and anisotropy was measured for 60 seconds. The best-fit curves from reaction simulations after globally fitting three independent concentration series experiments are shown as solid black lines for each concentration.

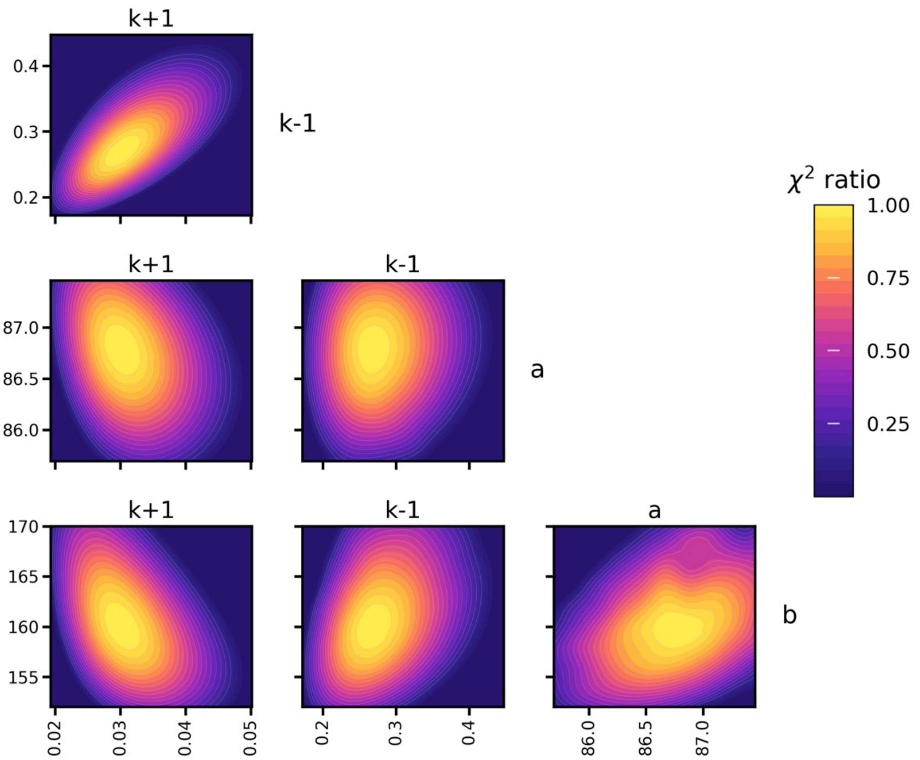

Figure S32. One-step reversible binding model for LexA(QM):RecA* interaction. (Top) Fitted parameters include the forward and reverse rate constants, k_1 and k_{-1}, along with two factors, a and b, used to convert the protein concentrations to simulated fluorescence anisotropy signals. (Bottom) Two-dimensional FitSpace confidence intervals for these fitted parameters, with higher Chi^2 ratios corresponding to better fits.

Table S10. Fitted values for reversible, one-step binding model parameters

**Parameter**	**Best fit value**	**Lower, Upper boundaries (Chi^2 threshold of 0.70)**
k_1	0.030	0.024, 0.038
k_{-1}	0.269	0.235, 0.313
a	86.780	86.300, 87.200
b	160.003	155.000, 166.000
C_1	1	N/A
C_2	0.98	0.97,0.99
C_3	0.97	0.96, 0.98

Table S11. Summary of binding model fit results

Model characteristic	Result
Data points	24978
Degrees of Freedom (DoF)	24974
Sigma w.r.t. fit	0.143
Chi2	35493
Chi2/DoF	1.42
p-value	0
Chi2 Threshold	0.999

Fluorescence Quenching Measurements (LexA conformational change)

All stopped-flow fluorescence intensity measurements were made in a KinTek AutoSF-120 stopped-flow spectrofluorometer, with the following settings. A Xenon/Mercury lamp (Hamamatsu) with a monochromator (Oriel) was set to emit light through a fiber optic at 385 nm with a 3.16 mm diffraction grating. Emitted light was filtered with a 440 +/− 40 nm emission filter (Edmund Optics) for either photomultiplier tube (PMT).

Mixtures of LexA$\tau_{86}\delta_{175}$ (K156A) at 1.5 μM and RecA* at different concentrations (1.5, 3, 5, 10, and 15 μM) were monitored for changes in fluorescence anisotropy for 60 seconds. A three-step kinetic model was used to fit the experimental data. Fits were optimized using built-in regression analysis in the software, similar to the previously mentioned fluorescence anisotropy data fitting.

$$\mathrm{LexA_1} \underset{\mathbf{k_{-1}}}{\overset{\mathbf{k_1}}{\rightleftharpoons}} \mathrm{LexA_2}$$

$$\mathrm{LexA_2} + \mathrm{RecA^*} \underset{\mathbf{k_{-2}}}{\overset{\mathbf{k_2}}{\rightleftharpoons}} \mathrm{LexA_2}:\mathrm{RecA^*}$$

$$\mathrm{LexA_2}:\mathrm{RecA^*} \underset{\mathbf{k_{-3}}}{\overset{\mathbf{k_3}}{\rightleftharpoons}} \mathrm{LexA^*}:\mathrm{RecA^*}$$

$$\mathrm{F} = \frac{\mathbf{a} \times [\mathrm{LexA_1}] + \mathbf{b} \times [\mathrm{LexA_2} + \mathrm{LexA_2}:\mathrm{RecA^*}] + \mathbf{c} \times [\mathrm{LexA^*}:\mathrm{RecA^*}]}{[\mathrm{LexA_1}] + [\mathrm{LexA_2}] + [\mathrm{LexA_2}:\mathrm{RecA^*}] + [\mathrm{LexA^*}:\mathrm{RecA^*}]}$$

Here,
LexA$_1$ = Open state
LexA$_2$ = Closed state
LexA* = RecA* optimized closed state (Cleavable state)

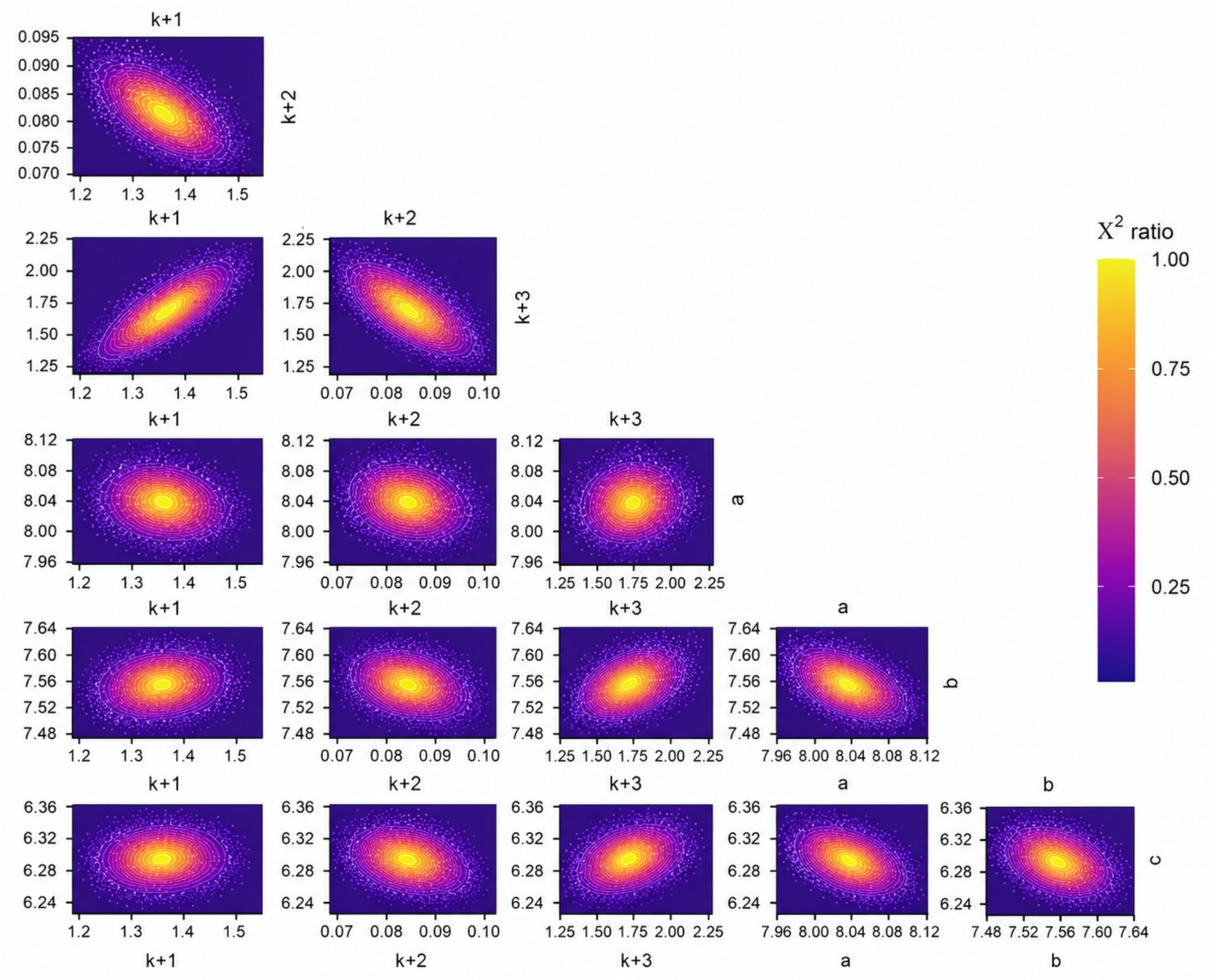

Figure S33. Three-step hybrid model for LexA conformational change. (Top) Fitted parameters include the forward equilibrium step, k_1, the RecA* binding step, k_2, and the RecA* stabilization step, k_3, along with three factors, a, b and c, used to convert the protein concentrations to simulated fluorescence signals. (Bottom) Two-dimensional FitSpace confidence intervals for these fitted parameters, with higher Chi^2 ratios corresponding to better fits.

Table S12. Fitted values for the three-step model parameters

Parameter	Best fit value	Lower, Upper boundaries (Chi^2 threshold of 0.70)
k_1	1.360	1.190, 1.560
k_{-1}	0.203 (fixed)	N/A
k_2	0.086	0.078, 0.094
k_{-2}	0.080	N/A
k_3	1.856	1.520, 2.270
k_{-3}	0.435 (fixed)	N/A
a	8.049	7.970, 8.120
b	7.589	7.530, 7.630
c	6.309	6.250, 6.360
C_1	1	N/A
C_2	0.99	0.98,1.00
C_3	0.96	0.95, 0.97

Table S13. Summary of binding model fit results

Model characteristic	Result
Data points	9995
Degrees of Freedom (DoF)	9991
Sigma w.r.t. fit	0.019
Chi2	12491
Chi2/DoF	1.250
p-value	0
Chi2 Threshold	0.999

Alternative models tested for LexA conformational step

Model 1 (Induced Fit):

$$\mathrm{LexA_1 + RecA^*} \underset{\mathbf{k_{-1}}}{\overset{\mathbf{k_1}}{\rightleftharpoons}} \mathrm{LexA_1{:}\,RecA^*}$$

$$\mathrm{LexA_1{:}\,RecA^*} \underset{\mathbf{k_{-2}}}{\overset{\mathbf{k_2}}{\rightleftharpoons}} \mathrm{LexA_2{:}\,RecA^*}$$

$$\mathrm{F} = \frac{\mathbf{a} \times [\mathrm{LexA_1 + LexA_1{:}\,RecA^*}] + \mathbf{b} \times [\mathrm{LexA_2{:}\,RecA^*}]}{[\mathrm{LexA_1}] + [\mathrm{LexA_2{:}\,RecA^*}] + [\mathrm{LexA_1{:}\,RecA^*}]}$$

Here,
$LexA_1$ = Open state
$LexA_2$ = Closed state

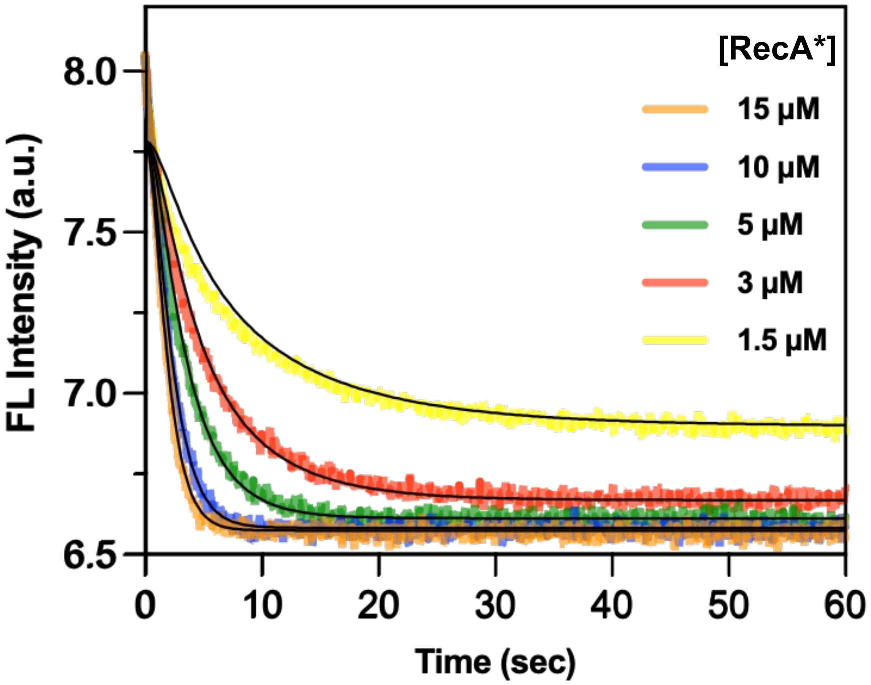

Figure S34. Fitting to an induced fit model (1). (Top) Fitted parameters include the RecA* binding step, k_1, and the RecA* induced conformational change step, k_2, along with two factors, a and b used to convert the protein concentrations to simulated fluorescence signals. (Bottom) The best-fit curves from reaction simulations after globally fitting three independent concentration series experiments are shown as solid black lines for each concentration.

Table S14. Fitted values for the two-step model parameters

Parameter	Best fit value	Lower, Upper boundaries (Chi^2 threshold of 0.70)
k_1	0.084 (fixed)	N/A
k_{-1}	0.016	0.013, 0.020
k_2	0.141	0.121, 0.176
k_{-2}	0.754	0.675, 0.921
a	7.783	7.730, 7.830
b	3.847×10^{-6}	3.850×10^{-9}, 8.850×10^{-5}

Table S15. Summary of binding model fit results

Model characteristic	Result
Data points	9995
Degrees of Freedom (DoF)	9992
Sigma w.r.t. fit	0.029
Chi2	32567
Chi2/DoF	3.26
p-value	0
Chi2 Threshold	0.999

Model 2 (Conformational Selection):

$$\mathrm{LexA_1} \underset{\mathbf{k_{-1}}}{\overset{\mathbf{k_1}}{\rightleftharpoons}} \mathrm{LexA_2}$$

$$\mathrm{LexA_2} + \mathrm{RecA^*} \underset{\mathbf{k_{-2}}}{\overset{\mathbf{k_2}}{\rightleftharpoons}} \mathrm{LexA_2{:}\,RecA^*}$$

$$\mathrm{F} = \frac{\mathbf{a} \times [\mathrm{LexA_1}] + \mathbf{b} \times [\mathrm{LexA_2} + \mathrm{LexA_2{:}\,RecA^*}]}{[\mathrm{LexA_1}] + [\mathrm{LexA_2}] + [\mathrm{LexA_2{:}\,RecA^*}]}$$

Here,
$\mathrm{LexA_1}$ = Open state
$\mathrm{LexA_2}$ = Closed state

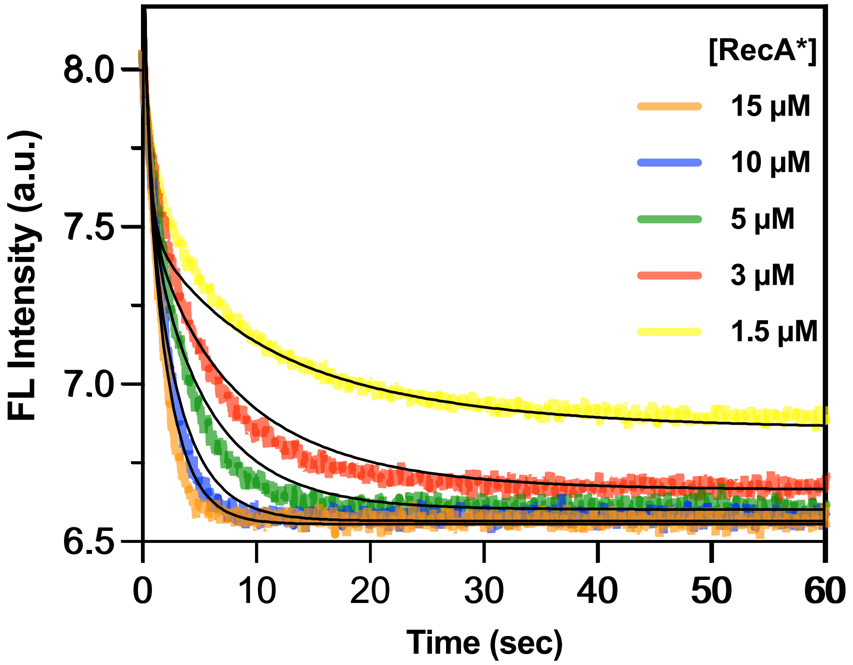

Figure S35. Fitting to a conformational selection model (2). (Top) Fitted parameters include the forward equilibrium step, k_1, and the RecA* binding step, along with two factors, a and b used to convert the protein concentrations to simulated fluorescence signals. (Bottom) The best-fit curves from reaction simulations after globally fitting three independent concentration series experiments are shown as solid black lines for each concentration.

Table S16. Fitted values for the two-step model parameters

Parameter	Best fit value	Lower, Upper boundaries (Chi^2 threshold of 0.70)
k_1	1.175	0.940, 1.840
k_{-1}	1.071	0.857, 1.670
k_2	0.084 (fixed)	N/A
k_{-2}	0.012	0.006, 0.019
a	8.482	8.340, 8.680
b	6.537	6.510, 6.560

Table S17. Summary of binding model fit results

Model characteristic	Result
Data points	9995
Degrees of Freedom (DoF)	9992
Sigma w.r.t. fit	0.040
Chi2	61339
Chi2/DoF	6.14
p-value	0
Chi2 Threshold	0.999

Model 3 (Not selected due to over-fitting of experimental data):

$$\mathrm{LexA_1} \underset{\mathbf{k_{-1}}}{\overset{\mathbf{k_1}}{\rightleftharpoons}} \mathrm{LexA_2}$$

$$\mathrm{LexA_1} + \mathrm{RecA^*} \underset{\mathbf{k_{-2}}}{\overset{\mathbf{k_2}}{\rightleftharpoons}} \mathrm{LexA_1{:}\,RecA^*}$$

$$\mathrm{LexA_1{:}\,RecA^*} \underset{\mathbf{k_{-3}}}{\overset{\mathbf{k_3}}{\rightleftharpoons}} \mathrm{LexA_2{:}\,RecA^*}$$

$$\mathrm{LexA_2} + \mathrm{RecA^*} \underset{\mathbf{k_{-4}}}{\overset{\mathbf{k_4}}{\rightleftharpoons}} \mathrm{LexA_2{:}\,RecA^*}$$

$$F = \frac{\mathbf{a} \times [\mathrm{LexA_1} + \mathrm{LexA_1{:}RecA^*}] + \mathbf{b} \times [\mathrm{LexA_2} + \mathrm{LexA_2{:}RecA^*}]}{[\mathrm{LexA_1}] + [\mathrm{LexA_2}] + [\mathrm{LexA_2{:}RecA^*}] + [\mathrm{LexA_1{:}RecA^*}]}$$

Here,
$LexA_1$ = Open state
$LexA_2$ = Closed state

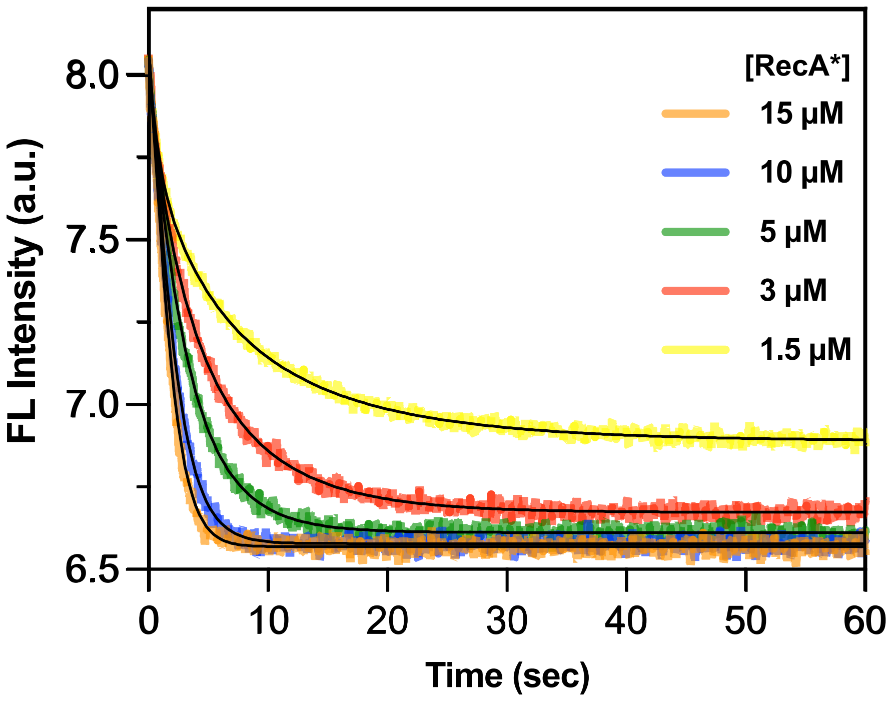

Figure S36. Fitting to an alternate conformational selection + induced fit model (3). (Top) Fitted parameters include the forward equilibrium step, k_1, the RecA* binding step, k_2, the RecA* induced conformational change step, k_3, and the second RecA* binding step, k_4, along with two factors, a and b used to convert the protein concentrations to simulated fluorescence signals. (Bottom) The best-fit curves from reaction simulations after globally fitting three independent concentration series experiments are shown as solid black lines for each concentration.

Table S18. Fitted values for the four-step model parameters

Parameter	Best fit value	Lower, Upper boundaries (Chi^2 threshold of 0.70)
k_1	0.338	0.044, 0.661
k_{-1}	1.012	0.648, 1.980
k_2	0.059	0.030, 0.099
k_{-2}	8.618×10^{-6}	8.620×10^{-9}, 8.620×10^{-5}
k_3	0.901	0.151, 1.760
k_{-3}	0.074	1.360×10^{-6}, 0.816
k_4	0.106	0.000, 0.691
k_{-4}	0.016	0.013, 0.092
a	8.048	7.980, 8.140
b	6.430	5.360×10^{-6}, 6.56

Table S19. Summary of binding model fit results

Model characteristic	Result
Data points	9995
Degrees of Freedom (DoF)	9986
Sigma w.r.t. fit	0.015
Chi2	6952
Chi2/DoF	0.69
p-value	1
Chi2 Threshold	0.999

LexA Cleavage Kinetics Using Fluorescence Intensity Measurement

All fluorescence intensity measurements were made using a Tecan Spark plate reader. Fluorescence intensity was measured at 450 nm with an excitation wavelength of 385 nm (5 nm bandwidth, 40 μs integration time, 31000 μm Z-position, and 3 sec linear shaking) using a nonsterile Greiner black, flat, 384-well low-volume microplate.

Mixtures of 0.5 μM LexA$\delta_{81}\tau_{86}$ and RecA* at different concentrations (0.25, 0.5, 1, 2.5, 5, 7.5, 15 μM) were monitored for changes in fluorescence intensity for 6 hours. The curves were imported into KinTek Explorer global fitting software. Sigma calculation and normalization for each trace were performed using the built-in afit (analytical fit) and concentration scaling factor analysis, respectively, present in the software. A three-step kinetic model was used to fit the experimental data. Fits were optimized using built-in regression analysis in the software, similar to the previously mentioned fluorescence anisotropy data fitting.

$$\mathrm{LexA} + \mathrm{RecA}^* \underset{\mathbf{k_{-1}}}{\overset{\mathbf{k_1}}{\rightleftharpoons}} \mathrm{LexA}^*:\mathrm{RecA}^*$$

$$\mathrm{LexA}^*:\mathrm{RecA}^* \xrightarrow{\mathbf{k_2}} \mathrm{LexA}^{**} + \mathrm{RecA}^*$$

$$\mathrm{F} = \frac{\mathbf{a} \times [\mathrm{LexA}] + \mathbf{b} \times [\mathrm{LexA}^{**}]}{[\mathrm{LexA}] + [\mathrm{LexA}^{**}]}$$

The following constraints were applied to better fit the experimental data to the above proposed model :

1. k_1 and k_{-1} were fixed as 0.084 and 0.079, respectively
2. k_{-2} fixed as 0

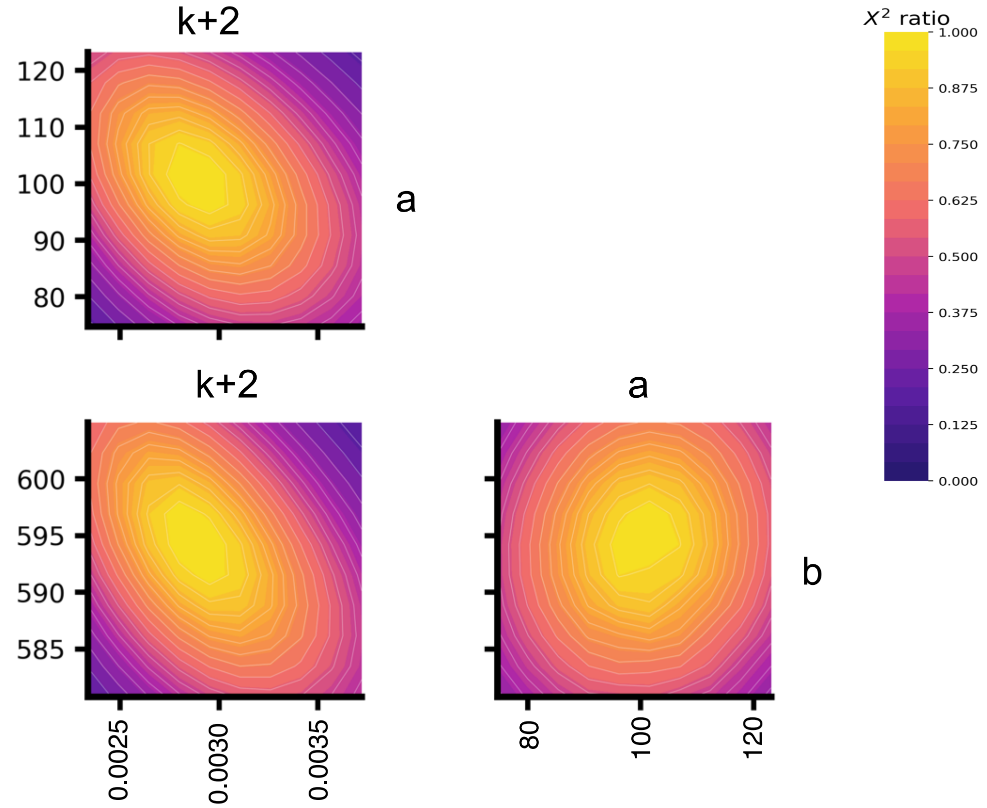

Figure S37. Two-step model for LexA cleavage. (Top) Fitted parameters include the forward rate constants for the autoproteolysis step (k_2), along with two factors (a and b) used to convert protein concentrations to simulated fluorescence signals. (Bottom) Two-dimensional FitSpace confidence intervals for these fitted parameters, with higher Chi^2 ratios corresponding to better fits.

Table S20. Fitted values for two-step cleavage model parameters

Parameter	Best fit value	Lower, Upper boundaries (Chi^2 threshold of 0.70)
k_1	0.084 (fixed)	N/A
k_{-1}	0.079 (fixed)	N/A
k_2	0.0030	0.0027, 0.0031
k_{-2}	0 (fixed)	N/A
a	103.71	90.80, 111.00
b	595.64	589.00, 600.00
C_1	1.00	N/A
C_2	0.96	0.95, 0.97
C_3	0.98	0.97, 0.99

Table S21. Summary of binding model fit results

Model characteristic	Result
Data points	1784
Degrees of Freedom (DoF)	1781
Sigma w.r.t. fit	0.1678
Chi2	2713.19
Chi2/DoF	1.52
p-value	0
Chi2 Threshold	0.9956

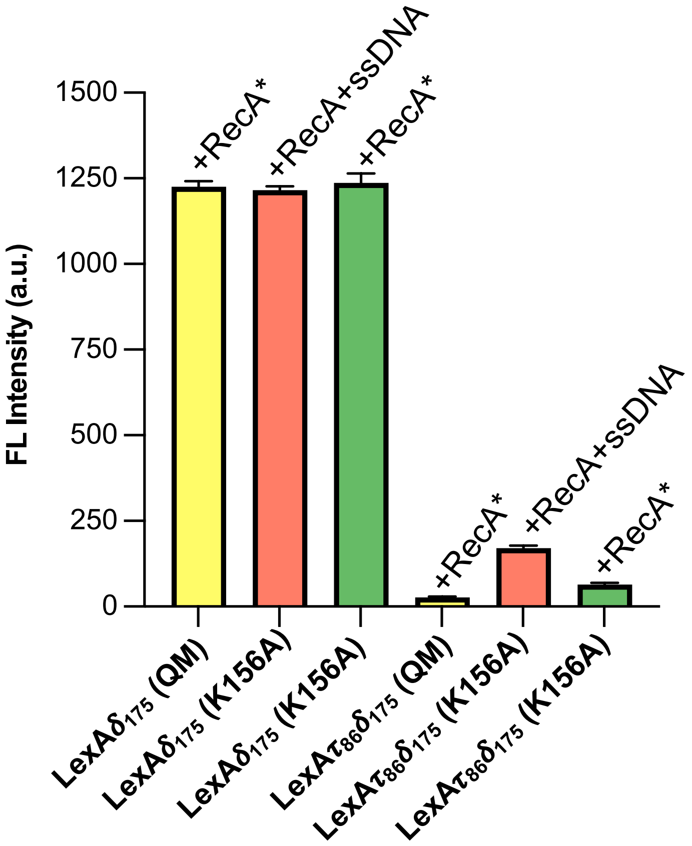

Figure S38. Control experiment to identify the effect of surrounding amino acids on Acd fluorescence quenching. Plot for changes in Acd (acridone) fluorescence intensity values on mixing activated RecA (RecA*) with single Acd LexA mutants, LexAδ_{175} (QM) and LexAδ_{175} (K156A), and dual Acd-Tet LexA mutants, LexA$\tau_{86}\delta_{175}$ (QM) and LexA$\tau_{86}\delta_{175}$ (K156A). Excitation and emission wavelengths for Acd were 385 nm and 450 nm, respectively. Error bars represent the standard deviation of three replicates.

RecA*-Mediated LexA$\delta_{81}\tau_{86}$ FRET Biosensor Assay:
RecA was activated by mixing 1 μM His-RecA and 2 μM 18-mer poly-GGT ssDNA (5'-GGT GGT GGT GGT GGT GGT-3' from Integrated DNA Technologies, Coralville, Iowa, USA.) in RecA activation buffer (70 mM Tris-HCl, pH 7.5, 10 mM $MgCl_2$, 2 mM TCEP, 150 mM NaCl, 50 µg/mL BSA, and 0.25 mM ATPγS). The reactions were incubated for 3 hours at 25 °C.
30 μM LexA$\delta_{81}\tau_{86}$ was pre-incubated for 10 minutes with 200 μM different small molecules (#1-20, Figure S41) before activated RecA* was then added 1:1 to give a final concentration of 15 μM LexA$\delta_{81}\tau_{86}$ and 100 μM of small molecule. The fluorescence intensity of Acd was monitored at 25 °C using a Tecan Spark microplate reader.

Gel analysis. After 18 hours from the time of addition of activated RecA * to LexA and small molecule mixes, 20 µL reaction mixture was added to 20 µL of 2x Laemmli buffer and boiled at 95 °C for 5 minutes. 40 µL of each sample was loaded onto a 15% SDS-PAGE gel, run at 150 V for 60 minutes, and stained with GelCode™ Blue Stain Reagent (Thermo Fisher) to visualize cleavage bands.

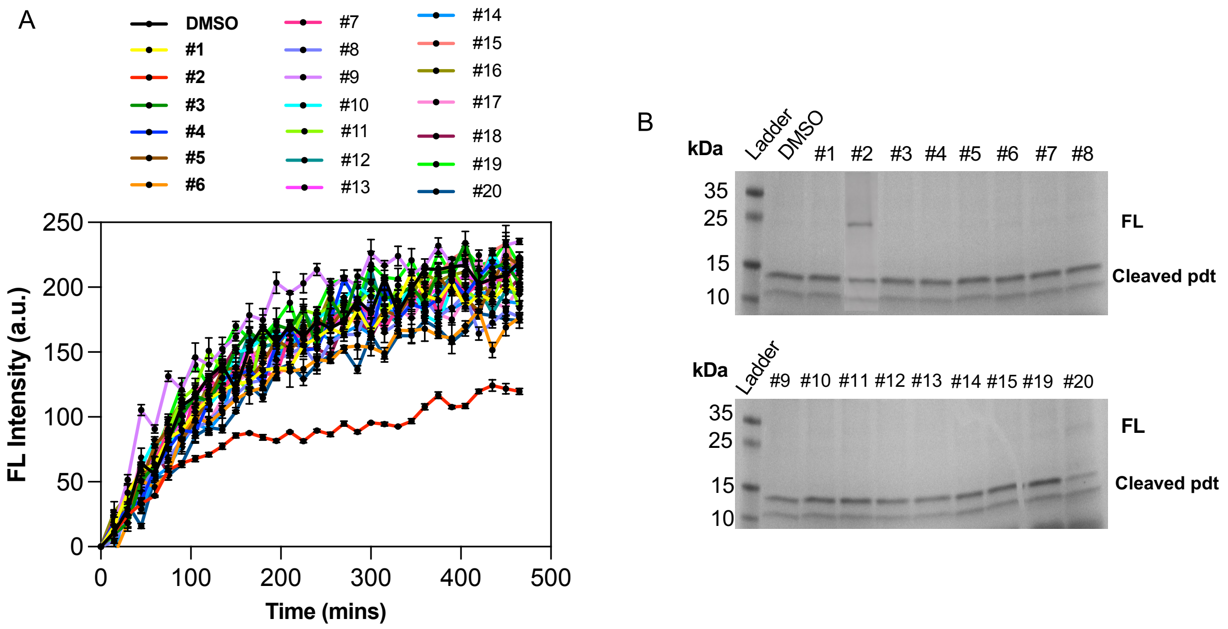

Figure S39. (A) Primary screening of small molecules using LexA$\delta_{81}\tau_{86}$ FRET biosensor on a 96-well plate. Error bars represent the standard deviation of three replicates. Excitation and emission wavelengths for Acd were 385 nm and 450 nm, respectively. (B) Gel-based LexA cleavage end point measurement after 18 hours from the addition of activated RecA*. FL=Full-length LexA

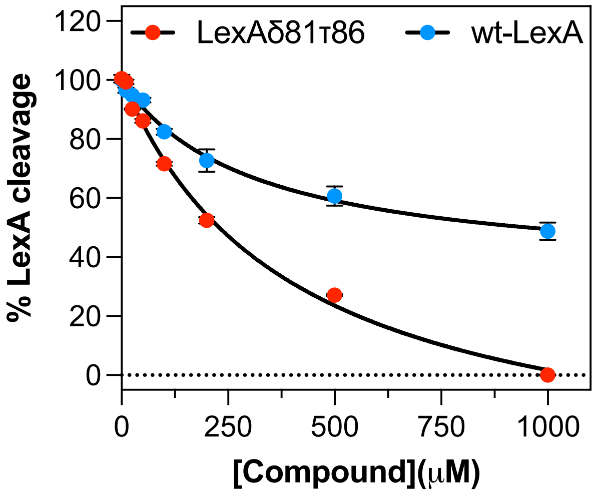

Figure S40. IC_{50} curves for inhibition of LexA$\delta_{81}\tau_{86}$ and WT-LexA cleavage by compound **2** evaluated in the 96-well plate–based fluorescence assay (red) and gel-based assay (blue), respectively.

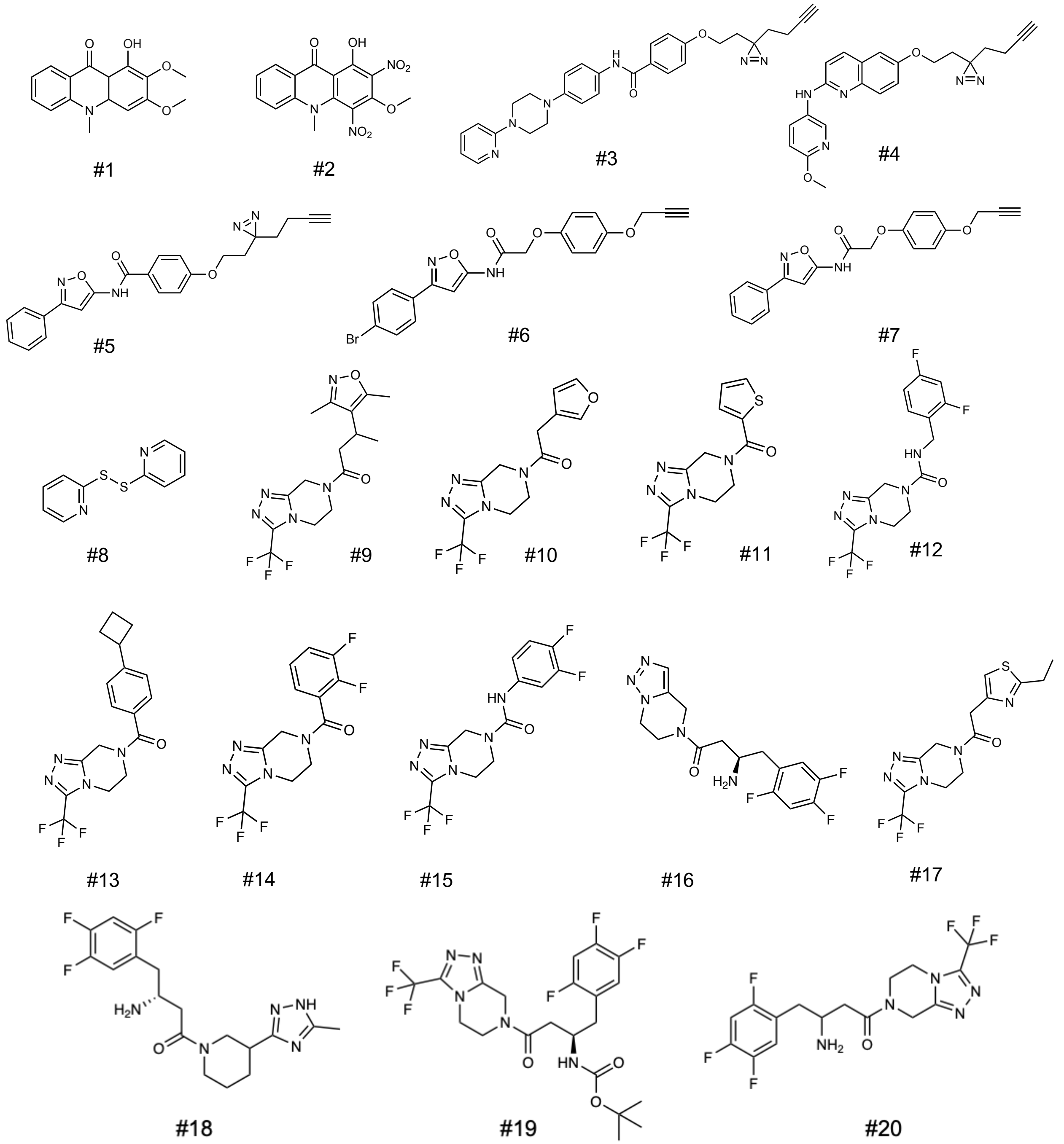

Figure S41. Chemical structures of small molecules screened against LexA$\delta_{81}\tau_{86}$ FRET biosensor.

1-hydroxy-2,3-dimethoxy-10-methylacridin-9(10H)-one (1): 1H NMR (600 MHz, CDCl3) δ 14.62 (s, 1H), 8.38 (d, J = 8.2 Hz, 1H), 7.76 – 7.69 (m, 1H), 7.47 (d, J = 8.7 Hz, 1H), 7.33 – 7.26 (m, 1H), 6.40 (s, 1H), 4.05 (s, 3H), 3.97 (s, 3H), 3.72 (s, 3H). 13C NMR (151 MHz, CDCl3) δ 181.9, 161.4, 160.1, 145.5, 138.6, 134.4, 129.1, 126.6, 121.7, 121.6, 115.7, 106.3, 93.9, 77.4, 77.2, 77.0, 61.7, 56.5, 40.7. HRMS (ESI$^+$) calcd for. $C_{16}H_{15}NO_4$ $[M + H]^+$, 286.1074; found: 286.1076

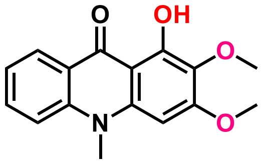

1-hydroxy-3-methoxy-10-methyl-2,4-dinitroacridin-9(10H)-one (2): 1H NMR (600 MHz, DMSO) δ 16.69 (s, 1H), 8.47 (d, J = 9.7 Hz, 1H), 7.92 (t, J = 7.9 Hz, 1H), 7.58 (d, J = 8.6 Hz, 1H), 7.54 (t, J = 8.0 Hz, 1H), 7.26 (s, 2H), 4.16 (s, 3H), 3.77 (s, 3H). 13C NMR (151 MHz, DMSO) δ 181.6, 161.0, 154.0, 143.9, 140.0, 136.4, 127.2, 126.9, 126.8, 125.0, 121.7, 117.0, 106.8, 65.6, 39.7. HRMS (ESI$^+$) calcd for. $C_{15}H_{11}N_3O_7$ $[M + H]^+$, 346.0670; found: 346.0670.

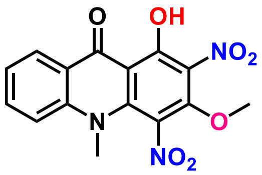

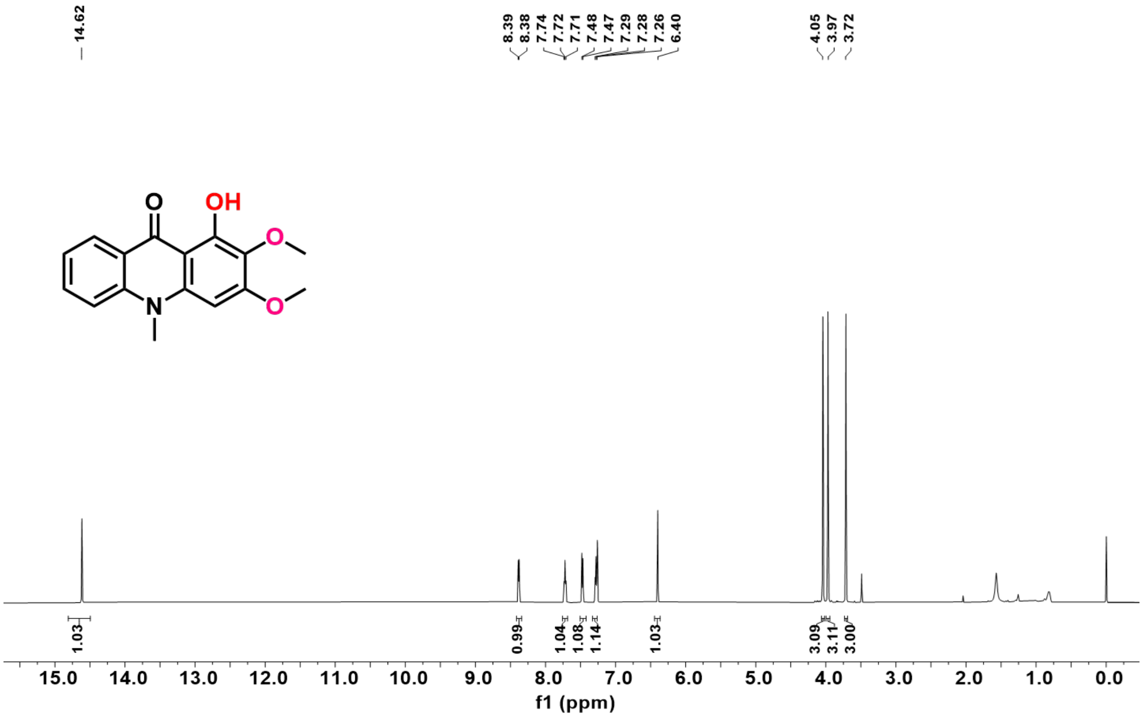

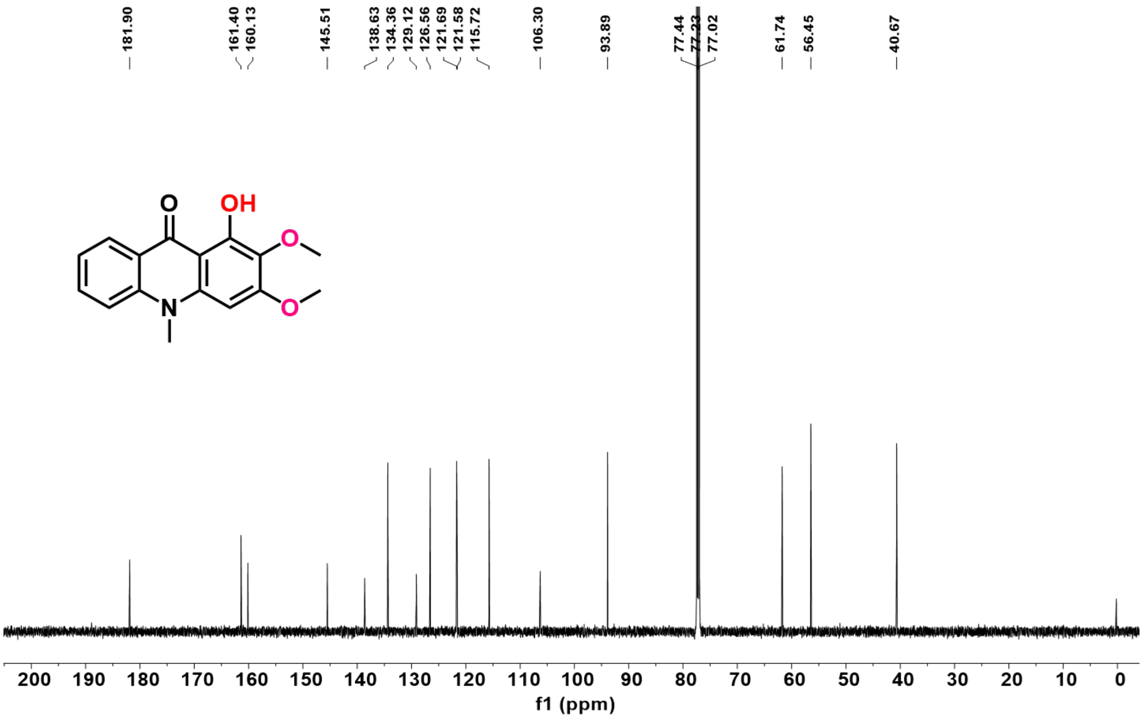

Figure S42. ^{1}H and ^{13}C NMR of the compound **1** in $CDCl_3$.

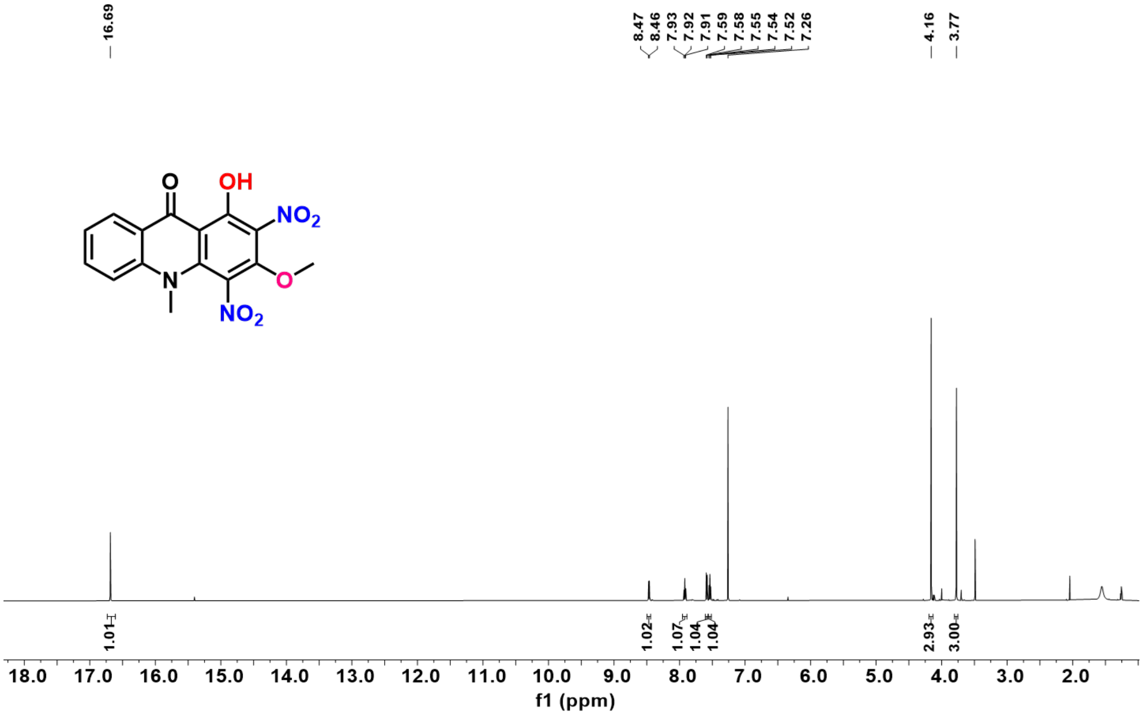

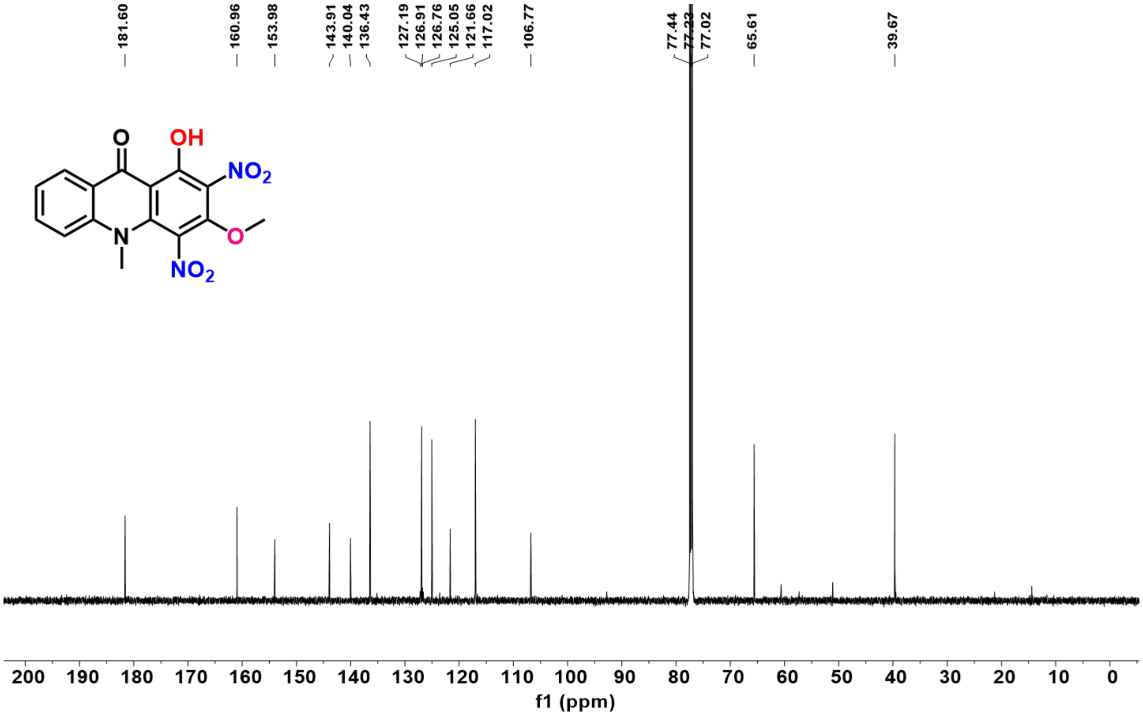

Figure S43. ^{1}H and ^{13}C NMR of the compound **2** in $CDCl_3$.

X-ray Structure Determination of Compound 1

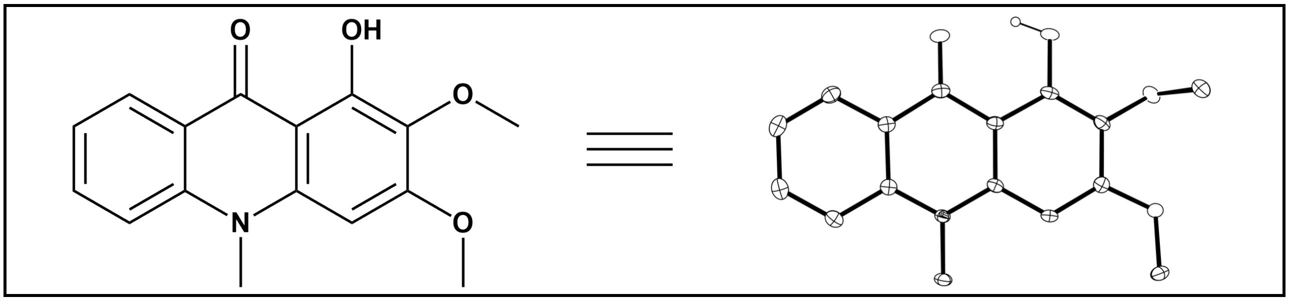

Compound **1**, $C_{16}H_{15}NO_4$, crystallizes in the orthorhombic space group Pbca (systematic absences hk0: h=odd, 0kl: k=odd, h0l: l=odd) with a=14.6718(6)Å, b=7.3756(3)Å, c=23.5504(8)Å, α=90°, β=90°, γ=90°, V=2548.47(17)Å^3, Z=8, and d_{calc}=1.487 g/cm^3. X-ray intensity data were collected on a Rigaku XtaLAB Synergy-S diffractometer [1] equipped with an HPC area detector (Dectris Pilatus3 R 200K) and employing confocal multilayer optic-monochromated Mo-Kα radiation (λ=0.71073 Å) at a temperature of 100K. Preliminary indexing was performed from a series of thirty 0.5° rotation frames with exposures of 10 seconds. A total of 1130 frames (10 runs) were collected employing ω scans with a crystal to detector distance of 34.0 mm, rotation widths of 0.5° and exposures of 50 seconds.

Rotation frames were integrated using CrysAlisPro [2], producing a listing of unaveraged F^2 and $\sigma(F^2)$ values. A total of 45565 reflections were measured over the ranges $4.436 \leq 2\theta \leq 56.556°$, $-17 \leq h \leq 19$, $-9 \leq k \leq 9$, $-31 \leq l \leq 31$ yielding 3148 unique reflections (R_{int} = 0.0550). The intensity data were corrected for Lorentz and polarization effects and for absorption using SCALE3 ABSPACK [3] (minimum and maximum transmission 0.48380, 1.00000). The structure was solved by dual space methods - SHELXT [4]. Refinement was by full-matrix least squares based on F^2 using SHELXL [5]. All reflections were used during refinement. The weighting scheme used was $w=1/[\sigma^2(F_o^2)+(0.0636P)^2+1.8077P]$ where $P=(F_o^2+2F_c^2)/3$. Non-hydrogen atoms were refined anisotropically and hydrogen atoms were refined using a riding model. Refinement converged to R1=0.0436 and wR2=0.1155 for 2713 observed reflections for which $F > 4\sigma(F)$ and R1=0.0509 and wR2=0.1201 and GOF =1.027 for all 3148 unique, non-zero reflections and 194 variables. The maximum Δ/σ in the final cycle of least squares was 0.001 and the two most prominent peaks in the final difference Fourier were +0.41 and -0.26 e/Å^3.

Table 1. lists cell information, data collection parameters, and refinement data. Final positional and equivalent isotropic thermal parameters are given in Tables 2. and 3. Anisotropic thermal parameters are in Table 4. Tables 5. and 6. list bond distances and bond angles. Figure 1. is an ORTEP representation of the molecule with 50% probability thermal ellipsoids displayed.

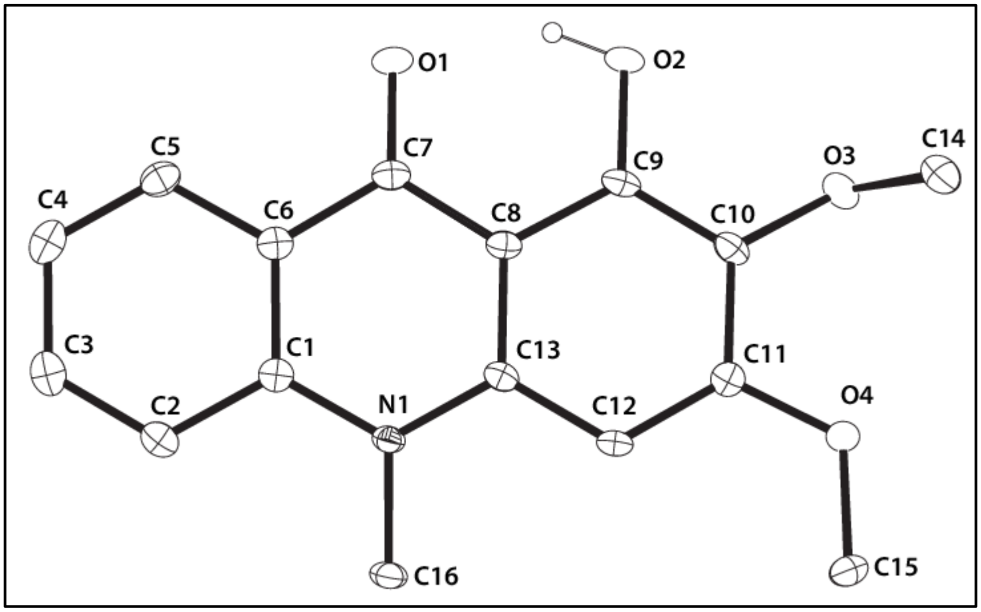

Figure S44. ORTEP drawing of the title compound **1** with 50% thermal ellipsoids.

Table S22. Summary of Structure Determination of Compound **1**

Empirical formula	$C_{16}H_{15}NO_4$
Formula weight	285.29
Diffractometer	Rigaku XtaLAB Synergy-S (Dectris Pilatus3 R 200K)
Temperature/K	100(2)
Crystal system	orthorhombic
Space group	Pbca
a	14.6718(6)Å
b	7.3756(3)Å
c	23.5504(8)Å
α	90°
β	90°
γ	90°
Volume	2548.47(17)$Å^3$
Z	8
d_{calc}	1.487 g/cm^3
μ	0.108 mm^{-1}
F(000)	1200.0
Crystal size, mm	0.497 × 0.068 × 0.057
2θ range for data collection	4.436 - 56.556°
Index ranges	-17 ≤ h ≤ 19, -9 ≤ k ≤ 9, -31 ≤ l ≤ 31
Reflections collected	45565
Independent reflections	3148[R(int) = 0.0550]
Data/restraints/parameters	3148/0/194
Goodness-of-fit on F^2	1.027
Final R indexes [I>=2σ (I)]	R_1 = 0.0436, wR_2 = 0.1155
Final R indexes [all data]	R_1 = 0.0509, wR_2 = 0.1201
Largest diff. peak/hole	0.41/-0.26 $eÅ^{-3}$

Table S23. Refined Positional Parameters for Compound **1**.

Atom	*x*	*y*	*z*	U(eq)
O1	0.45718(6)	0.31790(14)	0.53937(4)	0.0174(2)
O2	0.49033(6)	0.20360(14)	0.63814(4)	0.0179(2)
O3	0.42615(7)	0.07127(14)	0.73929(4)	0.0175(2)
O4	0.24816(6)	0.10849(14)	0.76466(4)	0.0167(2)
N1	0.18227(7)	0.30781(15)	0.57317(5)	0.0120(2)
C1	0.21240(9)	0.37955(17)	0.52197(5)	0.0123(3)
C2	0.15023(9)	0.44433(18)	0.48080(6)	0.0146(3)
C3	0.18161(10)	0.51609(19)	0.43044(6)	0.0169(3)
C4	0.27519(10)	0.52620(19)	0.41834(6)	0.0178(3)
C5	0.33655(9)	0.46440(18)	0.45797(6)	0.0156(3)
C6	0.30668(9)	0.39129(17)	0.51020(5)	0.0128(3)
C7	0.37291(9)	0.32348(17)	0.55085(6)	0.0129(3)
C8	0.33813(8)	0.26664(17)	0.60504(5)	0.0122(3)
C9	0.39956(9)	0.20823(18)	0.64859(6)	0.0135(3)
C10	0.36756(9)	0.15172(18)	0.70078(6)	0.0142(3)
C11	0.27293(9)	0.16063(18)	0.71163(5)	0.0132(3)
C12	0.21142(8)	0.21613(17)	0.67041(5)	0.0124(3)
C13	0.24309(8)	0.26443(17)	0.61610(5)	0.0113(3)
C14	0.44292(10)	0.1712(2)	0.79069(6)	0.0211(3)
C15	0.15480(9)	0.1363(2)	0.78077(6)	0.0207(3)
C16	0.08438(9)	0.27124(19)	0.58126(6)	0.0147(3)

Table S24. Positional Parameters for Hydrogens in Compound **1.**

**Atom**	***x***	***y***	***z***	**U(eq)**
H2	0.500362	0.240016	0.604932	0.027
H2A	0.086559	0.438346	0.487897	0.018
H3	0.138995	0.559676	0.403304	0.02
H4	0.295783	0.575036	0.383286	0.021
H5	0.399978	0.470947	0.450124	0.019
H12	0.148151	0.22149	0.678829	0.015
H14A	0.478992	0.279473	0.781958	0.032
H14B	0.476499	0.094634	0.817514	0.032
H14C	0.3847	0.207486	0.807621	0.032
H15A	0.115411	0.057176	0.758073	0.031
H15B	0.138051	0.263152	0.774117	0.031
H15C	0.147239	0.107543	0.821126	0.031
H16A	0.076779	0.165814	0.606137	0.022
H16B	0.056028	0.246433	0.544387	0.022
H16C	0.055229	0.377156	0.598621	0.022

Table S25. Refined Thermal Parameters (U's) for Compound **1.**

**Atom**	**U_{11}**	**U_{22}**	**U_{33}**	**U_{23}**	**U_{13}**	**U_{12}**
O1	0.0098(4)	0.0227(5)	0.0199(5)	0.0001(4)	0.0035(4)	-0.0012(4)
O2	0.0069(4)	0.0282(6)	0.0187(5)	-0.0003(4)	0.0010(4)	0.0007(4)
O3	0.0136(5)	0.0214(5)	0.0176(5)	0.0005(4)	-0.0038(4)	0.0040(4)
O4	0.0109(5)	0.0248(5)	0.0144(5)	0.0042(4)	0.0012(4)	0.0024(4)
N1	0.0082(5)	0.0148(5)	0.0128(5)	-0.0006(4)	-0.0006(4)	-0.0005(4)
C1	0.0135(6)	0.0099(6)	0.0135(6)	-0.0026(4)	0.0000(5)	-0.0002(4)
C2	0.0138(6)	0.0137(6)	0.0163(6)	-0.0027(5)	-0.0021(5)	0.0015(5)
C3	0.0225(7)	0.0133(6)	0.0150(6)	-0.0018(5)	-0.0030(5)	0.0028(5)
C4	0.0250(7)	0.0142(6)	0.0141(6)	-0.0001(5)	0.0027(5)	-0.0020(5)
C5	0.0164(6)	0.0137(6)	0.0169(6)	-0.0018(5)	0.0030(5)	-0.0023(5)
C6	0.0133(6)	0.0113(6)	0.0137(6)	-0.0031(5)	0.0005(5)	-0.0008(5)
C7	0.0114(6)	0.0109(6)	0.0162(6)	-0.0035(5)	0.0012(5)	-0.0015(4)
C8	0.0097(6)	0.0123(6)	0.0146(6)	-0.0024(5)	0.0002(5)	-0.0009(4)
C9	0.0082(6)	0.0145(6)	0.0178(6)	-0.0035(5)	-0.0007(5)	0.0008(5)
C10	0.0112(6)	0.0155(6)	0.0158(6)	-0.0009(5)	-0.0029(5)	0.0025(5)
C11	0.0142(6)	0.0118(6)	0.0137(6)	-0.0007(5)	0.0003(5)	0.0006(5)
C12	0.0076(5)	0.0137(6)	0.0158(6)	-0.0008(5)	0.0008(4)	0.0005(4)
C13	0.0099(6)	0.0094(6)	0.0144(6)	-0.0023(4)	-0.0014(5)	-0.0001(4)
C14	0.0158(7)	0.0296(8)	0.0180(7)	-0.0010(6)	-0.0035(5)	-0.0012(6)
C15	0.0126(7)	0.0312(8)	0.0184(7)	0.0073(6)	0.0048(5)	0.0029(6)
C16	0.0081(6)	0.0195(7)	0.0167(6)	-0.0007(5)	-0.0003(5)	-0.0005(5)

Table S26. Bond Distances in Compound **1, Å**

O1-C7	1.2663(16)	O2-C9	1.3547(15)	O3-C10	1.3834(16)
O3-C14	1.4383(17)	O4-C11	1.3563(16)	O4-C15	1.4361(16)
N1-C1	1.3891(16)	N1-C13	1.3859(16)	N1-C16	1.4737(16)
C1-C2	1.4143(18)	C1-C6	1.4134(17)	C2-C3	1.3780(19)
C3-C4	1.404(2)	C4-C5	1.375(2)	C5-C6	1.4128(18)
C6-C7	1.4528(18)	C7-C8	1.4371(18)	C8-C9	1.4317(18)
C8-C13	1.4186(17)	C9-C10	1.3801(19)	C10-C11	1.4133(18)
C11-C12	1.3872(18)	C12-C13	1.4067(17)		

Table S27. Bond Angles in Compound **1, °**

C10-O3-C14	116.00(11)	C11-O4-C15	117.28(10)	C1-N1-C16	119.47(11)
C13-N1-C1	121.08(11)	C13-N1-C16	119.41(11)	N1-C1-C2	121.23(12)
N1-C1-C6	120.33(11)	C6-C1-C2	118.43(12)	C3-C2-C1	120.29(12)
C2-C3-C4	121.45(13)	C5-C4-C3	119.01(12)	C4-C5-C6	120.97(13)
C1-C6-C7	120.29(12)	C5-C6-C1	119.84(12)	C5-C6-C7	119.84(12)
O1-C7-C6	121.57(12)	O1-C7-C8	121.79(12)	C8-C7-C6	116.62(11)
C9-C8-C7	120.03(12)	C13-C8-C7	121.02(11)	C13-C8-C9	118.94(12)
O2-C9-C8	119.76(12)	O2-C9-C10	119.25(12)	C10-C9-C8	120.97(12)
O3-C10-C11	120.79(12)	C9-C10-O3	120.13(12)	C9-C10-C11	118.77(12)
O4-C11-C10	114.61(11)	O4-C11-C12	123.62(12)	C12-C11-C10	121.75(12)
C11-C12-C13	119.74(12)	N1-C13-C8	119.76(11)	N1-C13-C12	120.60(11)
C12-C13-C8	119.64(11)				

This report has been created with Olex2 [6], compiled on 2022.04.07 svn.rca3783a0 for OlexSys.

X-ray Structure Determination of Compound 2

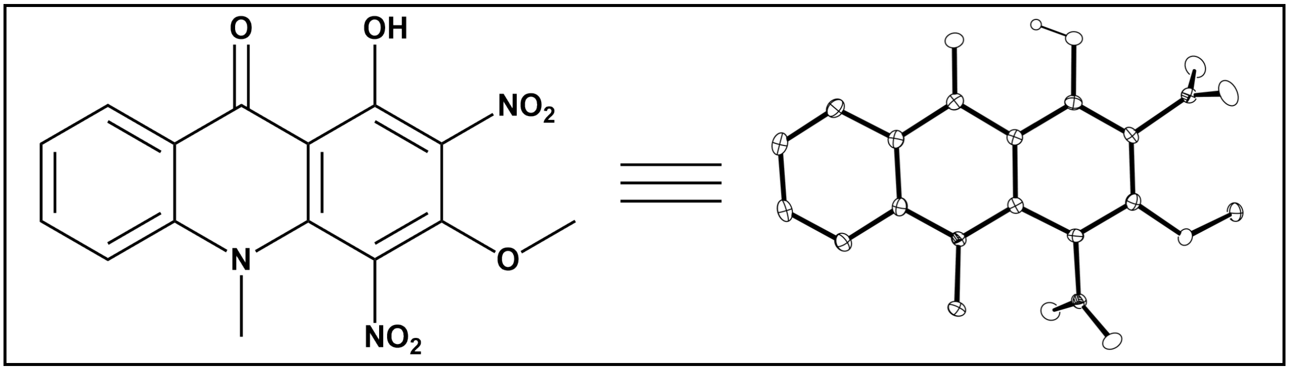

Compound **2**, $C_{15}H_{11}N_3O_7$, crystallizes in the monoclinic space group P2$_1$/c (systematic absences 0k0: k=odd and h0l: l=odd) with a=9.4121(4)Å, b=14.5547(6)Å, c=10.3081(5)Å, α=90°, β=99.071(4)°, γ=90°, V=1394.45(11)Å^3, Z=4, and d_{calc}=1.645 g/cm^3. X-ray intensity data were collected on a Rigaku XtaLAB Synergy-S diffractometer [1] equipped with an HPC area detector (Dectris Pilatus3 R 200K) and employing confocal multilayer optic-monochromated Mo-Kα radiation (λ=0.71073 Å) at a temperature of 100(K. Preliminary indexing was performed from a series of thirty 0.5° rotation frames with exposures of 1.25 seconds. A total of 1194 frames (13 runs) were collected employing ω scans with a crystal to detector distance of 34.0 mm, rotation widths of 0.5° and exposures of 13.25 seconds.

Rotation frames were integrated using CrysAlisPro [2], producing a listing of unaveraged F^2 and σ(F^2) values. A total of 27961 reflections were measured over the ranges 4.382 ≤ 2θ ≤ 56.56°, -12 ≤ h ≤ 12, -19 ≤ k ≤ 19, -13 ≤ l ≤ 13 yielding 3459 unique reflections (R_{int} = 0.0588). The intensity data were corrected for Lorentz and polarization effects and for absorption using SCALE3 ABSPACK [3] (minimum and maximum transmission 0.61353, 1.00000). The structure was solved by dual space methods - SHELXT [4]. Refinement was by full-matrix least squares based on F^2 using SHELXL [5]. All reflections were used during refinement. The weighting scheme used was $w=1/[\sigma^2(F_o^2)+(0.0487P)^2+0.5769P]$ where $P=(F_o^2+2F_c^2)/3$. Non-hydrogen atoms were refined anisotropically and hydrogen atoms were refined using a riding model. Refinement converged to R1=0.0357 and wR2=0.0948 for 2962 observed reflections for which F > 4σ(F) and R1=0.0433 and wR2=0.0984 and GOF =1.039 for all 3459 unique, non-zero reflections and 229 variables. The maximum Δ/σ in the final cycle of least squares was 0.000 and the two most prominent peaks in the final difference Fourier were +0.41 and -0.28 e/Å^3.

Table 1. lists cell information, data collection parameters, and refinement data. Final positional and equivalent isotropic thermal parameters are given in Tables 2. and 3. Anisotropic thermal parameters are in Table 4. Tables 5. and 6. list bond distances and bond angles. Figure 1. is an ORTEP representation of the molecule with 50% probability thermal ellipsoids displayed.

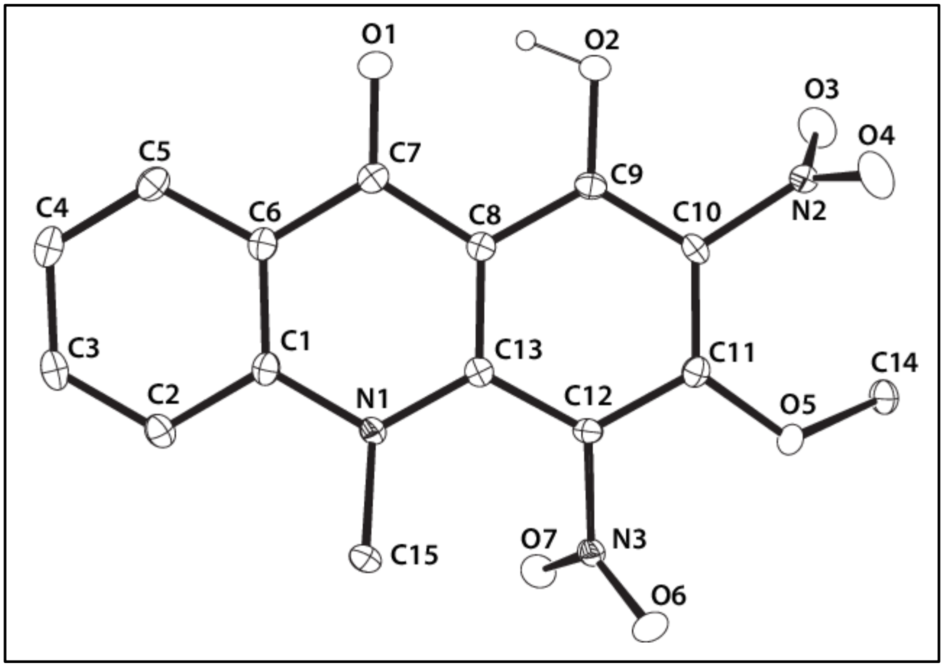

Figure S45. ORTEP drawing of the title compound **2** with 50% thermal ellipsoids.

Table S28. Summary of Structure Determination of Compound **2**

Empirical formula	$C_{15}H_{11}N_3O_7$
Formula weight	345.27
Diffractometer	Rigaku XtaLAB Synergy-S (Dectris Pilatus3 R 200K)
Temperature/K	100(2)
Crystal system	monoclinic
Space group	$P2_1/c$
a	9.4121(4)Å
b	14.5547(6)Å
c	10.3081(5)Å
α	90°
β	99.071(4)°
γ	90°
Volume	1394.45(11)$Å^3$
Z	4
d_{calc}	1.645 g/cm^3
μ	0.134 mm^{-1}
F(000)	712.0
Crystal size, mm	0.148 × 0.121 × 0.058
2θ range for data collection	4.382 - 56.56°
Index ranges	$-12 \leq h \leq 12$, $-19 \leq k \leq 19$, $-13 \leq l \leq 13$
Reflections collected	27961
Independent reflections	3459[R(int) = 0.0588]
Data/restraints/parameters	3459/0/229
Goodness-of-fit on F^2	1.039
Final R indexes [I>=2σ (I)]	R_1 = 0.0357, wR_2 = 0.0948
Final R indexes [all data]	R_1 = 0.0433, wR_2 = 0.0984
Largest diff. peak/hole	0.41/-0.28 $eÅ^{-3}$

Table S29. Refined Positional Parameters for Compound **2.**

**Atom**	***x***	***y***	***z***	**U(eq)**
O1	0.28892(9)	0.62651(6)	0.58474(8)	0.01562(19)
O2	0.11485(9)	0.64351(6)	0.37915(8)	0.01440(19)
O3	-0.03180(9)	0.59684(7)	0.11192(10)	0.0215(2)
O4	0.00712(10)	0.74100(7)	0.0791(1)	0.0230(2)
O6	0.58249(9)	0.68236(6)	0.00349(8)	0.0159(2)
O7	0.61864(9)	0.54913(6)	0.09872(9)	0.0160(2)
O5	0.29516(9)	0.66072(6)	-0.02924(8)	0.01397(19)
N1	0.62303(10)	0.63492(7)	0.37252(9)	0.0105(2)
N2	0.04674(10)	0.66430(7)	0.11766(10)	0.0131(2)
N3	0.55912(10)	0.62453(7)	0.08430(9)	0.0112(2)
C1	0.65068(12)	0.61198(8)	0.50591(11)	0.0113(2)
C2	0.79246(13)	0.59287(8)	0.56609(12)	0.0138(2)
C3	0.82043(13)	0.57456(8)	0.69931(12)	0.0150(2)
C4	0.71145(14)	0.57574(8)	0.77700(12)	0.0155(2)
C5	0.57145(13)	0.59182(8)	0.71829(12)	0.0139(2)
C6	0.53944(12)	0.60869(8)	0.58216(11)	0.0117(2)
C7	0.39191(12)	0.62438(8)	0.51989(11)	0.0114(2)
C8	0.36822(12)	0.63475(8)	0.37896(11)	0.0106(2)
C9	0.22463(12)	0.64253(8)	0.31258(11)	0.0111(2)
C10	0.19798(12)	0.65283(8)	0.17694(12)	0.0114(2)
C11	0.30979(12)	0.65403(8)	0.10240(11)	0.0110(2)
C12	0.45104(12)	0.64499(8)	0.16862(11)	0.0106(2)
C14	0.17003(13)	0.62357(9)	-0.11303(12)	0.0151(2)
C15	0.74413(12)	0.67173(9)	0.31328(12)	0.0148(2)
C13	0.48427(12)	0.63785(8)	0.30693(11)	0.0100(2)

Table S30. Positional Parameters for Hydrogens in Compound **2.**

Atom	*x*	*y*	*z*	U(eq)
H2	0.145976	0.639386	0.459914	0.022
H2A	0.868504	0.592549	0.515453	0.017
H3	0.916126	0.560839	0.739018	0.018
H4	0.733425	0.565614	0.869025	0.019
H5	0.496285	0.591502	0.769919	0.017
H14A	0.193622	0.611935	-0.200825	0.023
H14B	0.0907	0.667778	-0.119342	0.023
H14C	0.14124	0.565905	-0.07552	0.023
H15A	0.79433	0.621055	0.277277	0.022
H15B	0.811107	0.703941	0.380575	0.022
H15C	0.707355	0.71465	0.242666	0.022

Table S31. Refined Thermal Parameters (U's) for Compound **2**

Atom	U_{11}	U_{22}	U_{33}	U_{23}	U_{13}	U_{12}
O1	0.0134(4)	0.0217(5)	0.0127(4)	0.0004(3)	0.0049(3)	0.0016(3)
O2	0.0102(4)	0.0218(5)	0.0118(4)	0.0000(3)	0.0038(3)	0.0013(3)
O3	0.0143(4)	0.0237(5)	0.0254(5)	0.0016(4)	-0.0006(4)	-0.0070(4)
O4	0.0172(4)	0.0207(5)	0.0294(5)	0.0054(4)	-0.0019(4)	0.0061(4)
O6	0.0178(4)	0.0176(4)	0.0131(4)	0.0030(3)	0.0054(3)	-0.0019(3)
O7	0.0152(4)	0.0144(4)	0.0189(5)	-0.0006(3)	0.0041(3)	0.0035(3)
O5	0.0122(4)	0.0205(4)	0.0085(4)	0.0013(3)	-0.0005(3)	-0.0029(3)
N1	0.0082(4)	0.0127(5)	0.0104(5)	-0.0007(4)	0.0008(3)	-0.0010(4)
N2	0.0104(5)	0.0180(5)	0.0110(5)	0.0008(4)	0.0019(4)	0.0006(4)
N3	0.0094(4)	0.0137(5)	0.0103(5)	-0.0014(4)	0.0010(4)	-0.0013(4)
C1	0.0137(5)	0.0090(5)	0.0106(5)	-0.0013(4)	-0.0002(4)	-0.0012(4)
C2	0.0117(5)	0.0135(6)	0.0156(6)	0.0000(4)	0.0003(4)	-0.0013(4)
C3	0.0138(5)	0.0129(6)	0.0163(6)	-0.0008(4)	-0.0040(4)	-0.0003(4)
C4	0.0208(6)	0.0132(6)	0.0112(5)	0.0002(4)	-0.0017(5)	-0.0002(5)
C5	0.0182(6)	0.0121(6)	0.0117(6)	-0.0004(4)	0.0029(4)	-0.0002(4)
C6	0.0137(5)	0.0098(5)	0.0113(5)	-0.0009(4)	0.0008(4)	0.0001(4)
C7	0.0137(5)	0.0095(5)	0.0114(5)	-0.0008(4)	0.0027(4)	-0.0002(4)
C8	0.0109(5)	0.0098(5)	0.0110(5)	-0.0005(4)	0.0018(4)	-0.0001(4)
C9	0.0109(5)	0.0097(5)	0.0131(5)	-0.0008(4)	0.0036(4)	0.0000(4)
C10	0.0077(5)	0.0124(5)	0.0137(6)	0.0002(4)	0.0000(4)	0.0007(4)
C11	0.0131(5)	0.0091(5)	0.0105(5)	0.0006(4)	0.0010(4)	-0.0007(4)
C12	0.0093(5)	0.0117(5)	0.0114(5)	-0.0004(4)	0.0033(4)	-0.0004(4)
C14	0.0130(6)	0.0195(6)	0.0116(5)	-0.0025(5)	-0.0018(4)	-0.0020(5)
C15	0.0093(5)	0.0211(6)	0.0142(6)	0.0003(5)	0.0022(4)	-0.0036(4)
C13	0.0109(5)	0.0075(5)	0.0115(5)	-0.0007(4)	0.0012(4)	-0.0005(4)

Table S32. Bond Distances in Compound **2,** Å

O1-C7	1.2616(14)	O2-C9	1.3275(14)	O3-N2	1.2247(14)
O4-N2	1.2234(14)	O6-N3	1.2282(13)	O7-N3	1.2304(13)
O5-C11	1.3457(14)	O5-C14	1.4507(14)	N1-C1	1.3989(15)
N1-C15	1.4760(15)	N1-C13	1.3734(14)	N2-C10	1.4674(14)
N3-C12	1.4689(14)	C1-C2	1.4079(16)	C1-C6	1.4056(16)
C2-C3	1.3828(17)	C3-C4	1.3973(18)	C4-C5	1.3805(17)
C5-C6	1.4096(16)	C6-C7	1.4530(16)	C7-C8	1.4425(16)
C8-C9	1.4198(16)	C8-C13	1.4149(15)	C9-C10	1.3891(16)
C10-C11	1.3976(16)	C11-C12	1.4017(16)	C12-C13	1.4145(16)

Table S33. Bond Angles in Compound **2**, °

C11-O5-C14	121.18(9)	C1-N1-C15	117.21(9)	C13-N1-C1	120.42(9)
C13-N1-C15	121.43(10)	O3-N2-C10	117.87(10)	O4-N2-O3	124.41(10)
O4-N2-C10	117.71(10)	O6-N3-O7	124.49(10)	O6-N3-C12	118.50(9)
O7-N3-C12	117.00(9)	N1-C1-C2	119.78(10)	N1-C1-C6	121.21(10)
C6-C1-C2	119.00(11)	C3-C2-C1	119.63(11)	C2-C3-C4	121.66(11)
C5-C4-C3	119.14(11)	C4-C5-C6	120.36(11)	C1-C6-C5	120.09(11)
C1-C6-C7	119.46(10)	C5-C6-C7	120.44(11)	O1-C7-C6	122.02(11)
O1-C7-C8	121.43(11)	C8-C7-C6	116.52(10)	C9-C8-C7	118.55(10)
C13-C8-C7	121.48(10)	C13-C8-C9	119.97(10)	O2-C9-C8	120.74(10)
O2-C9-C10	119.17(10)	C10-C9-C8	120.05(10)	C9-C10-N2	116.16(10)
C9-C10-C11	121.51(10)	C11-C10-N2	122.32(10)	O5-C11-C10	126.09(10)
O5-C11-C12	115.87(10)	C10-C11-C12	118.02(10)	C11-C12-N3	115.03(10)
C11-C12-C13	122.67(10)	C13-C12-N3	121.19(10)	N1-C13-C8	119.58(10)
N1-C13-C12	122.71(10)	C12-C13-C8	117.71(10)		

This report has been created with Olex2 [6], compiled on 2022.04.07 svn.rca3783a0 for OlexSys.

Synthetase Sequences
pULTRAI-Tet3.0 RS (RS sequence underlined).

accaccctgaattgactctcttccgggcgctatcatgccataccgcgaaaggttttgcgccattcgatggtgtccgggatctcgacgctct
cccttatgcgactcctgcattagggagctgttgacaattaatcatcggctcgtataatgtgtggaattgtgagcggataacaatttcacaa
aggaggtgcggccgcatggataaaaaaccgctggatgtgctgattagcgcgaccggcctgtggatgagccgtaccggcaccctgc
ataaaatcaaacatcatgaagtgagccgcagcaaaatctatattgaaatggcgtgcggcgatcatctggtggtgaacaacagccgta
gctgccgtaccgcgcgtgcgtttcgtcatcataaataccgcaaaacctgcaaacgttgccgtgtgagcgatgaagatatcaacaacttt
ctgacccgtagcaccgaaagcaaaaacagcgtgaaagtgcgtgtggtgagcgcgccgaaagtgaaaaaagcgatgccgaaaa
gcgtgagccgtgcgccgaaaccgctggaaaatagcgtgagcgcgaaagcgagcaccaacaccagccgtagcgttccgagcccg
gcgaaaagcaccccgaacagcagcgttccggcgtctgcgccggcaccgagcctgacccgcagccagctggatcgtgtggaagcg
ctgctgtctccggaagataaaattagcctgaacatggcgaaaccgtttcgtgaactggaaccggaactggtgacccgtcgtaaaaac
gattttcagcgcctgtataccaacgatcgtgaagattatctgggcaaactggaacgtgatatcaccaaatttttgtggatcgcggctttct
ggaaattaaaagcccgattctgattccggcggaatatgtggaacgtatgggcattaacaacgacaccgaactgagcaaacaaatttt
ccgtgtggataaaaacctgtgcctgcgtccgatgctggccccgaccggttataactatttgcgtaaactggatcgtattctgccgggtccg
atcaaaatttttgaagtgggcccgtgctatcgcaaagaaagcgatggcaaagaacacctggaagaattcaccatggttggttttgctca
aatgggcagcggctgcacccgtgaaaacctggaagcgctgatcaaagaattcctggattatctggaaatcgacttcgaaattgtggg
cgatagctgcatggtgtatggcgataccctggatattatgcatggcgatctggaactgagcagcgcggtggtgggtccggttagcctgg
atcgtgaatggggcattgataaaccgtggattggcgcgggttttggcctggaacgtctgctgaaagtgatgcatggcttcaaaaacatta
aacgtgcgagccgtagcgaaagctactataacggcattagcacgaacctgtaagcggccgcgtttaaacggtctccagcttggctgtt
ttggcggatgagagaagattttcagcctgatacagattaaatcagaacgcagaagcggtctgataaaacagaatttgcctggcggca
gtagcgcggtggtcccacctgaccccatgccgaactcagaagtgaaacgccgtagcgccgatggtagtgtggggtctccccatgcg
agagtagggaactgccaggcatcaaataaaacgaaaggctcagtcgaaagactgggcctttgtttgtgagctcccggtcatcaatcat
ccccataatccttgttagcctgcaggtaattccgcttcgcaacatgtgagcaccggtttattgactaccggaagcagtgtgaccgtgtgctt
ctcaaatgcctgaggccagtttgctcaggctctccccgtggaggtaataattgacgatatgatcagtgcacggctaactaagcggcctg
ctgactttctcgccgatcaaaaggcattttgctattaagggattgacgagggcgtatctgcgcagtaagatgcgccccgcattcggaaa
cctgatcatgtagatcgaacggactttaaatccgttcagccgggttagattcccggggtttccgccaaattcgaaaagcctgctcaacg
agcaggctttttttgcatgctcgagcagctcagggtcgaatttgggtaccaccggcgcctcaggcatttgagaagcacacggtcacact
gcttccggtagtcaataaaccggtaaaccagcaatagacataagcggctatttaacgaccctgccctgaaccgacgaccgggtcatc
gtggccggatcttgcggcccctcggcttgaacgaattgttagacattatttgccgactaccttggtgatctcgcctttcacgtagtggacaa
attcttccaactgatctgcgcgcgaggccaagcgatcttcttcttgtccaagataagcctgtctagcttcaagtatgacgggctgatactg
ggccggcaggcgctccattgcccagtcggcagcgacatccttcggcgcgattttgccggttactgcgctgtaccaaatgcgggacaa
cgtaagcactacatttcgctcatcgccagcccagtcgggcggcgagttccatagcgttaaggtttcatttagcgcctcaaatagatcctgt
tcaggaaccggatcaaagagttcctccgccgctggacctaccaaggcaacgctatgttctcttgcttttgtcagcaagatagccagatc
aatgtcgatcgtggctggctcgaagatacctgcaagaatgtcattgcgctgccattctccaaattgcagttcgcgcttagctggataacg
ccacggaatgatgtcgtcgtgcacaacaatggtgacttctacagcgcggagaatctcgctctctccaggggaagccgaagtttccaa
aaggtcgttgatcaaagctcgccgcgttgtttcatcaagccttacggtcaccgtaaccagcaaatcaatatcactgtgtggcttcaggcc
gccatccactgcggagccgtacaaatgtacggccagcaacgtcggttcgagatggcgctcgatgacgccaactacctctgatagttg
agtcgatacttcggcgatcaccgcttccctcatactcttcctttttcaatattattgaagcatttatcagggttattgtctcatgagcggatacat
atttgaatgtatttagaaaaataaacaaatagctagctcactcggtcgctacgctccgggcgtgagactgcggcgggcgctgcggaca
catacaaagttacccacagattccgtggataagcaggggactaacatgtgaggcaaaacagcagggccgcgccggtggcgtttttc
cataggctccgcccctcctgccagagttcacataaacagacgcttttccggtgcatctgtgggagccgtgaggctcaaccatgaatctga
cagtacgggcgaaacccgacaggacttaaagatccccaccgtttccggcgggtcgctccctcttgcgctctcctgttccgaccctgccg
tttaccggatacctgttccgcctttctcccttacgggaagtgtggcgctttctcatagctcacacactggtatctcggctcggtgtaggtcgtt
cgctccaagctgggctgtaagcaagaactccccgttcagcccgactgctgcgccttatccggtaactgttcacttgagtccaacccgga

aaagcacggtaaaacgccactggcagcagccattggtaactgggagttcgcagaggatttgtttagctaaacacgcggttgctcttga
agtgtgcgccaaagtccggctacactggaaggacagatttggttgctgtgctctgcgaaagccagttaccacggttaagcagttcccc
aactgacttaaccttcgatcaaaccacctccccaggtggtttttcgtttacagggcaaaagattacgcgcagaaaaaaaggatctcaa
gaagatcctttgatcttttctactgaaccgctctagatttcagtgcaatttatctcttcaaatgtagcacctgaagtcagccccatacgatata
agttgtaattctcatgttagtcatgccccgcgcccaccggaaggagctgactgggttgaaggctctcaagggcatcggtcgagatccc
ggtgcctaatgagtgagctaacttacattaattgcgttgcgctcactgcccgctttccagtcgggaaacctgtcgtgccagctgcattaat
gaatcggccaacgcgcggggagaggcggtttgcgtattgggcgccagggtggtttttcttttcaccagtgagacgggcaacagctgat
tgcccttcaccgcctggccctgagagagttgcagcaagcggtccacgctggtttgccccagcaggcgaaaatcctgtttgatggtggtt
aacggcgggatataacatgagctgtcttcggtatcgtcgtatcccactaccgagatgtccgcaccaacgcgcagcccggactcggta
atggcgcgcattgcgcccagcgccatctgatcgttggcaaccagcatcgcagtgggaacgatgccctcattcagcatttgcatggtttgt
tgaaaaccggacatggcactccagtcgccttcccgttccgctatcggctgaatttgattgcgagtgagatatttatgccagccagccaga
cgcagacgcgccgagacagaacttaatgggcccgctaacagcgcgatttgctggtgacccaatgcgaccagatgctccacgccca
gtcgcgtaccgtcttcatgggagaaaataatactgttgatgggtgtctggtcagagacatcaagaaataacgccggaacattagtgca
ggcagcttccacagcaatggcatcctggtcatccagcggatagttaatgatcagcccactgacgcgttgcgcgagaagattgtgcacc
gccgctttacaggcttcgacgccgcttcgttctaccatcgacaccaccacgctggcacccagttgatcggcgcgagatttaatcgccgc
gacaatttgcgacggcgcgtgcagggccagactggaggtggcaacgccaatcagcaacgactgtttgcccgccagttgttgtgccac
gcggttgggaatgtaattcagctccgccatcgccgcttccactttttcccgcgttttcgcagaaacgtggctggcctggttcaccacgcgg
gaaacggtctgataagagacaccggcatactctgcgacatcgtataacgttactggtttcacattc

pEVOL-AcdA9 RS (RS sequence underlined).
aagaaaccaattgtccatattgcatcagacattgccgtcactgcgtcttttactggctcttctcgctaaccaaaccggtaaccccgcttatt
aaaagcattctgtaacaaagcgggaccaaagccatgacaaaaacgcgtaacaaaagtgtctataatcacggcagaaaagtccac
attgattatttgcacggcgtcacactttgctatgccatagcatttttatccataagattagcggatcctacctgacgctttttatcgcaactctct
actgtttctccatacccgtttttttgggctaacaggaggaattagatctatggacgaatttgaaatgataaagagaaacacatctgaaatt
atcagcgaggaagagttaagagaggttttaaaaaaagatgaaaaatctgctgcgataggttttgaaccaagtggtaaaatacatttag
ggcattatctccaaataaaaaagatgattgatttacaaaatgctggatttgatataattatagatttggctgatttacacgcctatttaaacc
agaaaggagagttggatgagattagaaaaataggagattataacaaaaaagtttttgaagcaatggggttaaaggcaaaatatgttt
atggaagtgaactggagcttgataaggattatacactgaatgtctatagattggctttaaaaactaccttaaaaagagcaagaaggagt
atggaacttatagcaagagaggatgaaaatccaaaggttgctgaagttatctatccaataatgcaggttaattctattcattatactggcg
ttgatgttgcagttggagggatggagcagagaaaaataaacatgttagcaagggagcttttaccaaaaaaggttgtttgtattcacaac
cctgtcttaacgggtttggatggagaaggaaagatgagttcttcaaaagggaattttatagctgttgatgactctccagaagagattagg
gctaagataaagaaagcatactgcccagctggagttgttgaaggaaatccaataatggagatagctaaatacttccttgaatatccttta
accataaaaaggccagaaaaatttggtggagatttgacagttaatagctatgaggagttagagagtttatttaaaaataaggaattgca
tccaatggatttaaaaaatgctgtagctgaagaacttataaagattttagagccaattagaaagagattatgatgagtttaaacggtctcc
agcttggctgttttggcggatgagagaagattttcagcctgatacagattaaatcagaacgcagaagcggtctgataaaacagaatttg
cctggcggcagtagcgcggtggtcccacctgaccccatgccgaactcagaagtgaaacgccgtagcgccgatggtagtgtggggtc
tccccatgcgagagtagggaactgccaggcatcaaataaaacgaaaggctcagtcgaaagactgggcctttgtttgtgagctcccggt
catcaatcatccccataatccttgttagattatcaattttaaaaaactaacagttgtcagcctgtcccgctttaatatcatacgccgttatacg
ttgtttacgctttgaggaatcccatatggacgaatttgaaatgataaagagaaacacatctgaaattatcagcgaggaagagttaagag
aggttttaaaaaaagatgaaaaatctgctgcgataggttttgaaccaagtggtaaaatacatttagggcattatctccaaataaaaaag
atgattgatttacaaaatgctggatttgatataattatagatttggctgatttacacgcctatttaaaccagaaaggagagttggatgagatt
agaaaaataggagattataacaaaaaagtttttgaagcaatggggttaaaggcaaaatatgtttatggaagtgaactggagcttgata
aggattatacactgaatgtctatagattggctttaaaaactaccttaaaaagagcaagaaggagtatggaacttatagcaagagagga
tgaaaatccaaaggttgctgaagttatctatccaataatgcaggttaattctattcattatactggcgttgatgttgcagttggagggatgga

gcagagaaaaataaacatgttagcaagggagcttttaccaaaaaaggttgtttgtattcacaaccctgtcttaacgggtttggatggag
aaggaaagatgagttcttcaaaagggaattttatagctgttgatgactctccagaagagattagggctaagataaagaaagcatactg
cccagctggagttgttgaaggaaatccaataatggagatagctaaatacttccttgaatatcctttaaccataaaaaggccagaaaaat
ttggtggagatttgacagttaatagctatgaggagttagagagtttatttaaaaataaggaattgcatccaatggatttaaaaaatgctgta
gctgaagaacttataaagattttagagccaattagaaagagattatgactgcagtttcaaacgctaaattgcctgatgcgctacgcttatc
aggcctacatgatctctgcaatatattgagtttgcgtgcttttgtaggccggataaggcgttcacgccgcatccggcaagaaacagcaa
acaatccaaaacgccgcgttcagcggcgtttttctgcttttcttcgcgaattaattccgcttcgcaacatgtgagcaccggtttattgactac
cggaagcagtgtgaccgtgtgcttctcaaatgcctgaggccagtttgctcaggctctccccgtggaggtaataattgacgatatgatcag
tgcacggctaactaagcggcctgctgactttctcgccgatcaaaaggcattttgctattaagggattgacgagggcgtatctgcgcagta
agatgcgccccgcattccggcggtagttcagcagggcagaacggcggactctaaatccgcatggcaggggttcaaatcccctccgc
cggaccaaattcgaaaagcctgctcaacgagcaggctttttgcatgctcgagcagctcagggtcgaatttgctttcgaatttctgccatt
catccgcttattatcacttattcaggcgtagcaaccaggcgtttaagggcaccaataactgccttaaaaaaattacgccccgccctgcc
actcatcgcagtactgttgtaattcattaagcattctgccgacatggaagccatcacaaacggcatgatgaacctgaatcgccagcgg
catcagcaccttgtcgccttgcgtataatatttgcccatggtgaaaacgggggcgaagaagttgtccatattggccacgtttaaatcaaa
actggtgaaactcacccagggattggctgagacgaaaaacatattctcaataaaccctttagggaaataggccaggttttcaccgtaa
cacgccacatcttgcgaatatatgtgtagaaactgccggaaatcgtcgtggtattcactccagagcgatgaaaacgtttcagtttgctcat
ggaaaacggtgtaacaagggtgaacactatcccatatcaccagctcaccgtctttcattgccatacggaattccggatgagcattcatc
aggcgggcaagaatgtgaataaaggccggataaaacttgtgcttatttttctttacggtctttaaaaaggccgtaatatccagctgaacg
gtctggttataggtacattgagcaactgactgaaatgcctcaaaatgttctttacgatgccattgggatatatcaacggtggtatatccagt
gatttttttctccattttagcttccttagctcctgaaaatctcgataactcaaaaaatacgcccggtagtgatcttatttcattatggtgaaagtt
ggaacctcttacgtgccgatcaacgtctcattttcgccaaaagttggcccagggcttcccggtatcaacagggacaccaggatttatttat
tctgcgaagtgatcttccgtcacaggtatttattcggcgcaaagtgcgtcgggtgatgctgccaacttactgatttagtgtatgatggtgttttt
gaggtgctccagtggcttctgtttctatcagctgtccctcctgttcagctactgacggggtggtgcgtaacggcaaaagcaccgccggac
atcagcgctagcggagtgtatactggcttactatgttggcactgatgagggtgtcagtgaagtgcttcatgtggcaggagaaaaaagg
ctgcaccggtgcgtcagcagaatatgtgatacaggatatattccgcttcctcgctcactgactcgctacgctcggtcgttcgactgcggc
gagcggaaatggcttacgaacggggcggagatttcctggaagatgccaggaagatacttaacagggaagtgagagggccgcggc
aaagccgtttttccataggctccgcccccctgacaagcatcacgaaatctgacgctcaaatcagtggtggcgaaacccgacaggact
ataaagataccaggcgtttccccctggcggctccctcgtgcgctctcctgttcctgcctttcggtttaccggtgtcattccgctgttatggccg
cgtttgtctcattccacgcctgacactcagttccgggtaggcagttcgctccaagctggactgtatgcacgaaccccccgttcagtccga
ccgctgcgccttatccggtaactatcgtcttgagtccaacccggaaagacatgcaaaagcaccactggcagcagccactggtaattg
atttagaggagttagtcttgaagtcatgcgccggttaaggctaaactgaaaggacaagttttggtgactgcgctcctccaagccagttac
ctcggttcaaagagttggtagctcagagaaccttcgaaaaaccgccctgcaaggcggttttttcgttttcagagcaagagattacgcgc
agaccaaaacgatctcaagaagatcatcttattaatcagataaaatatttctagatttcagtgcaatttatctcttcaaatgtagcacctga
agtcagccccatacgatataagttgtaattctcatgtttgacagcttatcatcgataagcttggtacccaattatgacaacttgacggctac
atcattcactttttcttcacaaccggcacggaactcgctcgggctggccccggtgcattttttaaatacccgcgagaaatagagttgatcgt
caaaaccaacattgcgaccgacggtggcgataggcatccgggtggtgctcaaaagcagcttcgcctggctgatacgttggtcctcgc
gccagcttaagacgctaatccctaactgctggcggaaaagatgtgacagacgcgacggcgacaagcaaacatgctgtgcgacgct
ggcgatatcaaaattgctgtctgccaggtgatcgctgatgtactgacaagcctcgcgtacccgattatccatcggtggatggagcgact
cgttaatcgcttccatgcgccgcagtaacaattgctcaagcagatttatcgccagcagctccgaatagcgcccttccccttgcccggcgt
taatgatttgcccaaacaggtcgctgaaatgcggctggtgcgcttcatccgggcgaaagaaccccgtattggcaaatattgacggcca
gttaagccattcatgccagtaggcgcgcggacgaaagtaaacccactggtgataccattcgcgagcctccggatgacgaccgtagt
gatgaatctctcctggcgggaacagcaaaatatcactcggtcggcaaacaaattctcgtccctgatttttcaccaccccctgaccgcga
atggtgagattgagaatataacctttcattcccagcggtcggtcgataaaaaaatcgagataaccgttggcctcaatcggcgttaaacc
cgccaccagatgggcattaaacgagtatcccggcagcaggggatcattttgcgcttcagccatacttttcatactcccgccattcagag

REFERENCES:

1. Marmorstein, J. G.; Pagar, V. V.; Hummingbird, E.; Saleh, I. G.; Phan, H. A. T.; Chang, Y.; Shaffer, K. D.; Venkatesh, Y.; Dmochowski, I. J.; Stebe, K. J.; Petersson, E. J., Improved Large-Scale Synthesis of Acridonylalanine for Diverse Peptide and Protein Applications. *Bioconjugate Chemistry* **2024,** *35* (12), 1913-1922.
2. Jang, H. S.; Jana, S.; Blizzard, R. J.; Meeuwsen, J. C.; Mehl, R. A., Access to Faster Eukaryotic Cell Labeling with Encoded Tetrazine Amino Acids. *Journal of the American Chemical Society* **2020,** *142* (16), 7245-7249.
3. Speight, L. C.; Muthusamy, A. K.; Goldberg, J. M.; Warner, J. B.; Wissner, R. F.; Willi, T. S.; Woodman, B. F.; Mehl, R. A.; Petersson, E. J., Efficient Synthesis and In Vivo Incorporation of Acridon-2-ylalanine, a Fluorescent Amino Acid for Lifetime and Förster Resonance Energy Transfer/Luminescence Resonance Energy Transfer Studies. *Journal of the American Chemical Society* **2013,** *135* (50), 18806-18814.
4. Van Der Meer, B. W.; Coker III, G.; Chen, S.-Y. S., *Resonance Energy Transfer*. VCH Publishers, Inc.: New York, NY U.S.A., 1994; p 177.
5. Wu, P. G.; Brand, L., RESONANCE ENERGY-TRANSFER - METHODS AND APPLICATIONS. *Anal. Biochem.* **1994,** *218* (1), 1-13.
6. Szymanska, A.; Wegner, K.; Lankiewicz, L., Synthesis of N-[(tert-butoxy)carbonyl]-3-(9,10-dihydro-9-oxoacridin-2-yl)-L-alanine, a new fluorescent amino acid derivative. *Helv. Chim. Acta* **2003,** *86* (10), 3326-3331.
7. Jones, C. M.; Robkis, D. M.; Blizzard, R. J.; Munari, M.; Venkatesh, Y.; Mihaila, T. S.; Eddins, A. J.; Mehl, R. A.; Zagotta, W. N.; Gordon, S. E.; Petersson, E. J., Genetic encoding of a highly photostable, long lifetime fluorescent amino acid for imaging in mammalian cells. *Chem Sci* **2021,** *12* (36), 11955-11964.
8. Batjargal, S.; Walters, C. R.; Petersson, E. J., Inteins as Traceless Purification Tags for Unnatural Amino Acid Proteins. *Journal of the American Chemical Society* **2015,** *137* (5), 1734-1737.
9. Ferrie, J. J.; Ieda, N.; Haney, C. M.; Walters, C. R.; Sungwienwong, I.; Yoon, J.; Petersson, E. J., Multicolor protein FRET with tryptophan, selective coumarin-cysteine labeling, and genetic acridonylalanine encoding. *Chem Commun (Camb)* **2017,** *53* (80), 11072-11075.
10. Haney, C. M.; Cleveland, C. L.; Wissner, R. F.; Owei, L.; Robustelli, J.; Daniels, M. J.; Canyurt, M.; Rodriguez, P.; Ischiropoulos, H.; Baumgart, T.; Petersson, E. J., Site-Specific Fluorescence Polarization for Studying the Disaggregation of α-Synuclein Fibrils by Small Molecules. *Biochemistry* **2017,** *56* (5), 683-691.
11. Ferrie, J. J.; Haney, C. M.; Yoon, J.; Pan, B.; Lin, Y.-C.; Fakhraai, Z.; Rhoades, E.; Nath, A.; Petersson, E. J., Using a FRET Library with Multiple Probe Pairs To Drive Monte Carlo Simulations of α-Synuclein. *Biophysical Journal* **2018,** *114* (1), 53-64.
12. Hostetler, Z. M.; Ferrie, J. J.; Bornstein, M. R.; Sungwienwong, I.; Petersson, E. J.; Kohli, R. M., Systematic Evaluation of Soluble Protein Expression Using a Fluorescent Unnatural Amino Acid Reveals No Reliable Predictors of Tolerability. *ACS Chem. Biol.* **2018,** *13* (10), 2855-2861.
13. Bednar, R. M.; Jana, S.; Kuppa, S.; Franklin, R.; Beckman, J.; Antony, E.; Cooley, R. B.; Mehl, R. A., Genetic Incorporation of Two Mutually Orthogonal Bioorthogonal Amino Acids That Enable Efficient Protein Dual-Labeling in Cells. *ACS Chem. Biol.* **2021,** *16* (11), 2612-2622.
14. Chin, J. W.; Martin, A. B.; King, D. S.; Wang, L.; Schultz, P. G., Addition of a photocrosslinking amino acid to the genetic code of Escherichia coli. *Proceedings of the National Academy of Sciences* **2002,** *99* (17), 11020-11024.
15. Sungwienwong, I.; Hostetler, Z. M.; Blizzard, R. J.; Porter, J. J.; Driggers, C. M.; Mbengi, L. Z.; Villegas, J. A.; Speight, L. C.; Saven, J. G.; Perona, J. J.; Kohli, R. M.; Mehl, R. A.; Petersson, E. J., Improving target amino acid selectivity in a permissive aminoacyl tRNA synthetase through counter-selection. *Org. Biomol. Chem.* **2017,** *15* (17), 3603-3610.

16. Zhang, Y.; Werling, U.; Edelmann, W., SLiCE: a novel bacterial cell extract-based DNA cloning method. *Nucleic Acids Research* **2012,** *40* (8), e55-e55.
17. Cory, M. B.; Li, A.; Hurley, C. M.; Carman, P. J.; Pumroy, R. A.; Hostetler, Z. M.; Perez, R. M.; Venkatesh, Y.; Li, X.; Gupta, K.; Petersson, E. J.; Kohli, R. M., The LexA–RecA* structure reveals a cryptic lock-and-key mechanism for SOS activation. *Nat. Struct. Mol. Biol.* **2024,** *31* (10), 1522-1531.
18. Blizzard, R. J.; Backus, D. R.; Brown, W.; Bazewicz, C. G.; Li, Y.; Mehl, R. A., Ideal Bioorthogonal Reactions Using A Site-Specifically Encoded Tetrazine Amino Acid. *Journal of the American Chemical Society* **2015,** *137* (32), 10044-10047.
19. Eddins, A. J.; Bednar, R. M.; Jana, S.; Pung, A. H.; Mbengi, L.; Meyer, K.; Perona, J. J.; Cooley, R. B.; Karplus, P. A.; Mehl, R. A., Truncation-Free Genetic Code Expansion with Tetrazine Amino Acids for Quantitative Protein Ligations. *Bioconjugate Chemistry* **2023,** *34* (12), 2243-2254.
